# The diverse genetic origins of a Classical period Greek army

**DOI:** 10.1073/pnas.2205272119

**Published:** 2022-10-03

**Authors:** Laurie J. Reitsema, Alissa Mittnik, Britney Kyle, Giulio Catalano, Pier Francesco Fabbri, Adam C. S. Kazmi, Katherine L. Reinberger, Luca Sineo, Stefano Vassallo, Rebecca Bernardos, Nasreen Broomandkhoshbacht, Kim Callan, Francesca Candilio, Olivia Cheronet, Elizabeth Curtis, Daniel Fernandes, Martina Lari, Ann Marie Lawson, Matthew Mah, Swapan Mallick, Kirsten Mandl, Adam Micco, Alessandra Modi, Jonas Oppenheimer, Kadir Toykan Özdogan, Nadin Rohland, Kristin Stewardson, Stefania Vai, Chiara Vergata, J. Noah Workman, Fatma Zalzala, Valentina Zaro, Alessandro Achilli, Achilles Anagnostopoulos, Cristian Capelli, Varnavas Constantinou, Hovirag Lancioni, Anna Olivieri, Anastasia Papadopoulou, Nikoleta Psatha, Ornella Semino, John Stamatoyannopoulos, Ioanna Valliannou, Evangelia Yannaki, Iosif Lazaridis, Nick Patterson, Harald Ringbauer, David Caramelli, Ron Pinhasi, David Reich

**Affiliations:** ^a^Department of Anthropology, University of Georgia, Athens, GA 30602;; ^b^Department of Human Evolutionary Biology, Harvard University, Cambridge, MA 02138;; ^c^Department of Genetics, Harvard Medical School, Boston, MA 02115;; ^d^Max Planck−Harvard Research Center for the Archaeoscience of the Ancient Mediterranean, 04103 Leipzig, Germany;; ^e^Max Planck−Harvard Research Center for the Archaeoscience of the Ancient Mediterranean, Cambridge, MA 02138;; ^f^Department of Archaeogenetics, Max Planck Institute for Evolutionary Anthropology, 04103 Leipzig, Germany;; ^g^Department of Anthropology, University of Northern Colorado, Greeley, CO 80639;; ^h^Dipartimento di Scienze e Tecnologie Biologiche Chimiche e Farmaceutiche, Università degli Studi di Palermo, Palermo, 90123 Italy;; ^i^Department of Cultural Heritage, Università del Salento, 73100 Lecce, Italy;; ^j^Soprintendenza per i Beni Culturali e Ambientali di Palermo, 90143 Palermo, Italy;; ^k^Howard Hughes Medical Institute (HHMI), Harvard Medical School, Boston, MA 02115;; ^l^Servizio di Bioarcheologia, Museo delle Civiltà, 00144 Rome, Italy;; ^m^Department of Evolutionary Anthropology, Universität Wien, 1090 Vienna, Austria;; ^n^CIAS, Department of Life Sciences, Universidade de Coimbra, 3000-456 Coimbra, Portugal;; ^o^Dipartimento di Biologia, Università degli Studi di Firenze, Florence, 50122 Italy;; ^p^Broad Institute of MIT and Harvard, Cambridge, MA 02142;; ^q^Department of Biology and Biotechnology “L. Spallanzani,” Università di Pavia, Pavia 27100, Italy;; ^r^Gene and Cell Therapy Center, Hematology-HCT Unit, General Hospital of Thessaloniki “George Papanikolaou”, 55710 Thessaloniki, Greece;; ^s^Department of Zoology, University of Oxford, Oxford OX1 3SZ, United Kingdom;; ^t^Department of Chemistry, Life Sciences and Environmental Sustainability, Università di Parma, 43124 Parma, Italy;; ^u^Department of Chemistry, Biology and Biotechnology, Università degli Studi di Perugia, 06123 Perugia, Italy;; ^v^Department of Medicine, University of Washington, Seattle, WA 98195;; ^w^Department of Genome Sciences, University of Washington, Seattle, WA 98195;; ^x^Human Evolution and Archaeological Sciences, Universität Wien, A-1030 Vienna, Austria

**Keywords:** ancient DNA, archaeology, history, Classical world, ancient warfare

## Abstract

Trade and colonization caused an unprecedented increase in Mediterranean human mobility in the first millennium BCE. Often seen as a dividing force, warfare is in fact another catalyst of culture contact. We provide insight into the demographic dynamics of ancient warfare by reporting genome-wide data from fifth-century soldiers who fought for the army of the Greek Sicilian colony of Himera, along with representatives of the civilian population, nearby indigenous settlements, and 96 present-day individuals from Italy and Greece. Unlike the rest of the sample, many soldiers had ancestral origins in northern Europe, the Steppe, and the Caucasus. Integrating genetic, archaeological, isotopic, and historical data, these results illustrate the significant role mercenaries played in ancient Greek armies and highlight how participation in war contributed to continental-scale human mobility in the Classical world.

## Significance

By studying genome-wide data from 54 individuals from eighth- to fifth-century Sicily, we gain insights into the composition of Classical Greek armies (ca. fifth c. BCE) and the populace of a Greek colony. The presence of mercenaries in Greek armies fighting in the Mediterranean, as early as 480 BCE and with origins as far away as northern Europe and the Caucasus, is absent from historical texts and thus so far underappreciated in ancient classical scholarship. Our interdisciplinary study both underlines the value of integrating genetic studies to complement archaeological and historical research and highlights the importance of warfare in facilitating continental-scale human mobility, cultural contact, and cooperation in the Mediterranean of the Classical period.

The classical Mediterranean world was characterized by long-distance interactions mediated not only by trade and colonization (1–4) but also by political and military altercations often precipitated by the need to defend newly established settlements and trade routes that arose due to the expansionist strategies by Greeks, Phoenicians, and others (3, 5). Violent conflict is a common theme of ancient written accounts, and to better understand these ancient societies, it is important to test alternative hypotheses about the role of military activity in their population interactions (6–8).

There is extensive archaeological evidence for links between prehistoric Sicily and the Eastern Mediterranean, as documented by material culture exchange beginning at least in the Early Bronze Age (4, 9–11). This early contact is also reflected in genomic data showing the appearance of an Iranian-related ancestry component from the mid-second millennium BCE onward (12), a type of ancestry that, up to this period of the Mediterranean, had only been detected in regions further to the East (13–15). The establishment of Phoenician trading posts on the western coasts of Sicily, previously entirely inhabited by local Iron Age ethnic groups, began in the ninth century BCE and was followed by a systematic colonization by Greeks in the eighth century BCE, according to archaeological and textual evidence (3, 9, 10, 16–18). Although published ancient DNA data from the Greek world is still sparse, data are available from Late Bronze Age (LBA) people who lived in the Aegean, sometimes referred to as “Mycenaeans”, who are consistent with being the primary source population for present-day people in Greece (13). Inhabitants of the Greek colony of Empúries in Iberia living in the fifth century BCE have clear genetic linkages to LBA Aegean people as well as locals (19), a contribution to people of the island by people with this LBA Aegean genetic signature (20). However, beyond that, little genetic data exist to evaluate diversity of Greek colonies, despite extensive archaeological evidence for diverse identities in Greek settlements throughout the Mediterranean and especially on Sicily (3, 10, 16, 17, 21–24).

**Fig. 1.**
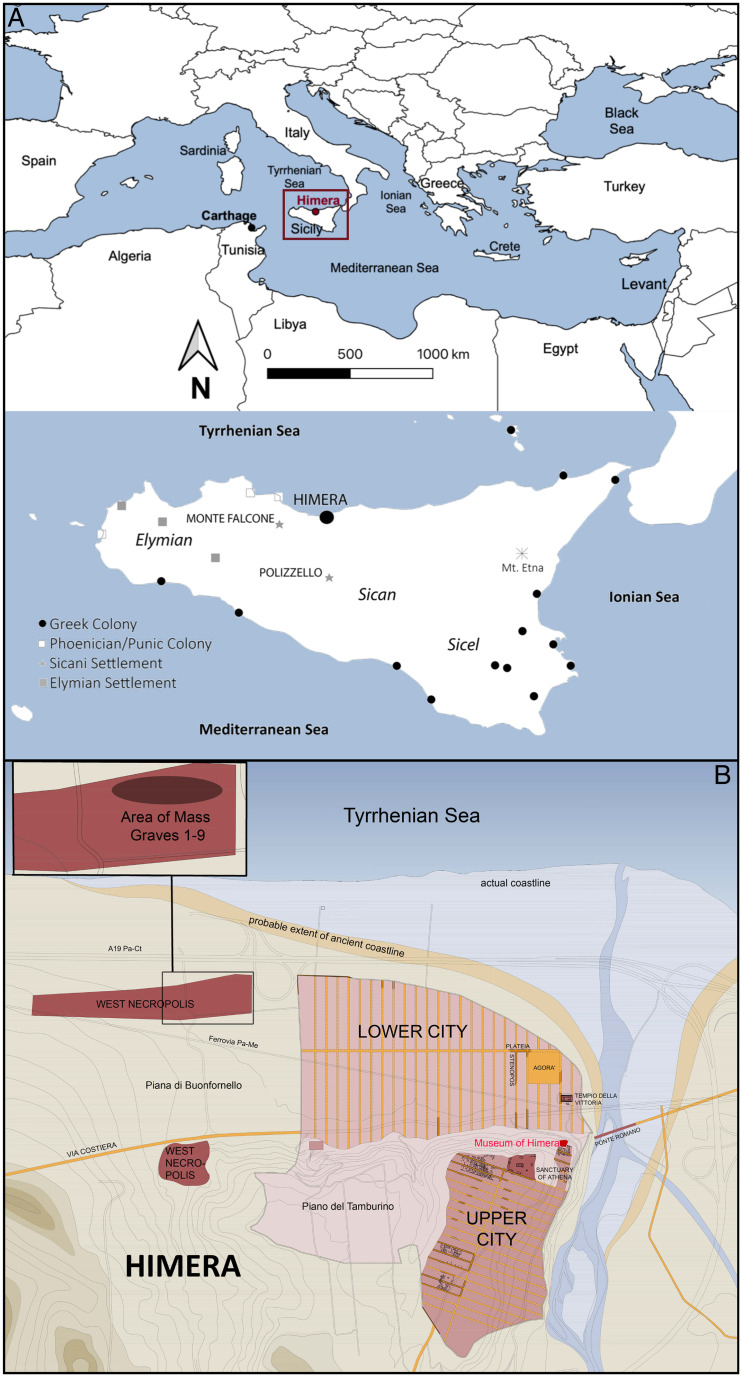
Location of the study area. (*A*) Maps showing Sicily’s location in the Mediterranean and archaeological sites of Greek, Punic, and indigenous settlements in the sixth century BCE, including those of the present study (Himera, Monte Falcone, Polizzello). Regions of influence of three indigenous groups are indicated in italics. (*B*) Schematic of the archaeological site of Himera, indicating the location of mass graves within the West necropolis (the East necropolis is outside the map area).

Himera was a colony founded by Ionian and Dorian Greeks around 648 BCE (25, 26) (Fig. 1). Himera was also likely inhabited by indigenous Sicilians, Punic people, and Etruscans (25, 27), as interactions between these groups have been well documented at Greek settlements, such as Selinunte (28). Himera was the westernmost Greek settlement in northern Sicily (as Selinunte was in the south) and was adjacent to nearby indigenous Sicilian and Punic settlements (18, 29, 30). Ancient authors describe two battles fought at Himera in 480 BCE and 409 BCE between Greeks and Carthaginians, and mass graves containing adult males unearthed in Himera’s West necropolis are believed to contain soldiers who died in the two battles (31–33) (*SI Appendix*, Figs. S1–S7 and Table S1). According to written sources, Himera won the battle of 480 BCE, owing to relief forces sent by its Greek allies in Syracuse and Agrigento (Diod. 11.20–25) (34), but lost the battle of 409 BCE, when Himerans fought unaided (Diod. 13.60–62; Xen. Hel. 1.1.37) (35). Himera was destroyed and ceased to function as an autonomous city-state (polis) after its loss in 409 BCE.

Archaic and Classical Greek armies are often understood as being primarily composed of hoplites, self-funded Greek citizen-soldiers who wore heavy armor to defend their poleis and allied settlements (36–38). The role of mercenaries in Greek armies is frequently downplayed by ancient historians, with mercenaries often being presented as inferior to heroic Greek hoplites (39, 40). Mercenaries’ employment in the Greek world typically came about through relationships between aristocratic families, who established international exchange of goods, services, and military aid when necessary (7, 41). In the Classical period on mainland Greece, these ties were mostly subsumed by international relations between the larger poleis, except in times of political strife, such as the rise of Athenian mercenaries in the fourth centuries (6). Mercenaries were also hired as bodyguards for tyrants in both mainland Greece and Sicily (6, 7). While the role of mercenaries in Greek armies is less clear, Punic armies, such as Carthage, often used mercenary armies (8, 42), and Greek tyrants on Sicily likely did the same (43).

Recently, strontium and oxygen isotope evidence from the mass graves corroborated historical accounts by revealing a large number of nonlocal individuals in mass graves associated with 480 BCE and mostly local individuals in mass graves associated with 409 BCE (37). Significantly, the isotopic evidence suggests that many of the nonlocal soldiers from 480 BCE originated from further afield than Sicily, which contrasts with the ancient historians who write only of Greek allies from elsewhere in Sicily (Diod. 11.20–25; Hdt. 7.165–7) (44) and highlights the possibility of a larger role for mercenaries in Greek armies than is commonly appreciated (6, 7). While isotopic analyses provide information about whether people traveled during their lifetime, genetic data provide complementary evidence by revealing likely geographic origins of people’s ancestors. Here, we analyze genome-wide data from 33 individuals associated with the Battles of Himera and from Himera’s civilian population, as well as 21 individuals from two nearby settlements associated with the indigenous Sicani culture of Sicily, to provide insight into the genetic ancestry of Sicily’s inhabitants in the first millennium BCE and to provide additional data points for evaluating the role of ancient conflict in population interactions in the ancient Mediterranean.

## Results

We generated double- and single-stranded next-generation sequencing libraries (45, 46) both using partial uracil-DNA glycosylase (UDG) treatment and without such treatment for two samples and enriched them for ∼1.2 million single-nucleotide polymorphisms (SNPs) (47, 48) (Dataset S1). Filtering for standard quality criteria and using a cutoff of at least 10,000 covered SNPs, we retain a dataset of 16 individuals buried in mass graves associated with the 480 BCE battle (*SI Appendix*, Figs. S1–S6), 5 associated with the 409 BCE battle (*SI Appendix*, Fig. S7), 11 attributed to the civilian population buried in Himera’s West necropolis, and 1 attributed to the civilian population buried at the East necropolis (Dataset S2). All of the soldiers are genetically sexed as males, while seven civilian individuals are genetically sexed as male and the other five as female. A total of 19 individuals were analyzed from the Iron Age site of Polizzello (ninth and eighth centuries BCE) and 2 from the site of Monte Falcone at Baucina (sixth to fifth centuries BCE), representing both males and females. One pair of second-degree relatives was detected at Polizzello (*SI Appendix*, Fig. S8). Overall, the average coverage on samples from the new dataset ranges from 0.011- to 4.298-fold on targeted SNPs on the autosomes with between 11,393 and 905,741 SNPs covered. Nuclear contamination point estimates on the X chromosome, which can be estimated for males, ranged from 0 to 1.6%. To contextualize the newly reported ancient individuals, we also generated new genotyping data for 96 modern-day Italian, Greek, and Cretan individuals on the Affymetrix Human Origins (HO) SNP set (49) (*SI Appendix*, Fig. S9 and Dataset S3).

**Fig. 2.**
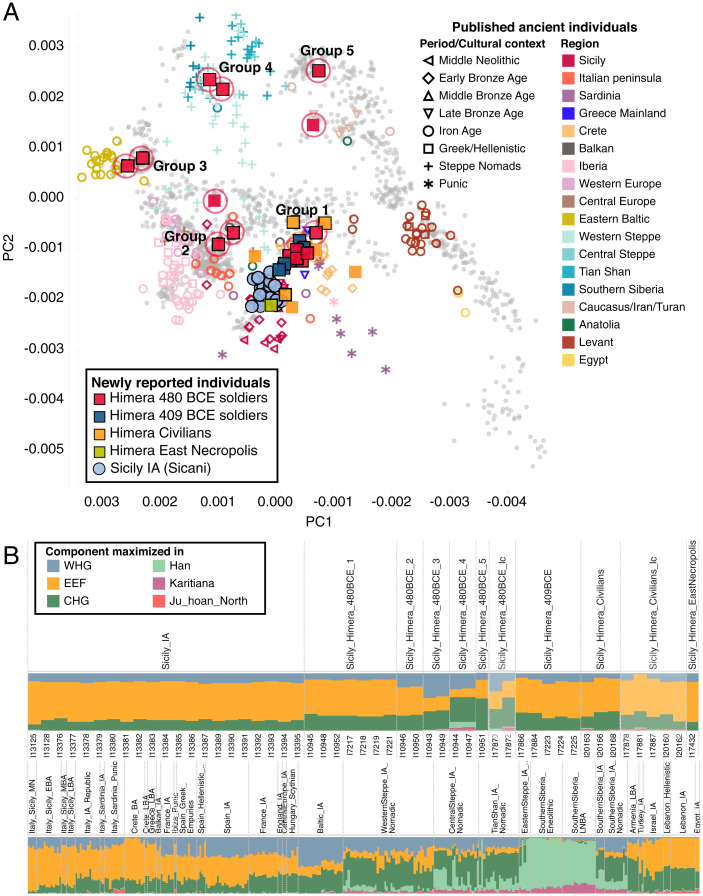
PCA and ADMIXTURE results for Sicilian individuals and relevant ancient samples from the literature. (*A*) Ancient data for individuals with data at more than 10,000 HO SNPs projected onto the first two PCs constructed with 1,081 modern-day west Eurasian and North African individuals, represented by small gray circles (*SI Appendix*, Fig. S8 includes population labels). Color and symbol indicate geographic origin and cultural affiliation of ancient samples, respectively. Circled symbols indicate individuals with strontium isotope signatures inconsistent with an origin in Sicily. Symbols without a black outline denote samples covered on fewer than 50,000 1240K SNPs. (*B*) Results of unsupervised ADMIXTURE, showing k = 6 for new Sicilian data and select ancient samples. Samples covered on fewer than 50,000 1240K SNPs shown at lower opacity. Full results are shown in *SI Appendix*, Fig. S1.

We computed principal components (PCs) on the newly generated HO data, together with 985 previously published present-day individuals from 64 diverse West Eurasian and North African populations (49), and projected new and published ancient individuals (Datasets S3 and S4) onto the first two PCs (Fig. 2*A*). As previously reported (49), modern Western Eurasians form two parallel clines separated on the first PC, which distinguishes European populations from populations of the Near East and Caucasus, while the second PC roughly reflects the north–south geographical distribution of the populations. Modern Aegean and Balkan populations occupy the space between the two parallel clines, forming a genetic and geographic bridge. We ran unsupervised ADMIXTURE (50) on a worldwide set of 1,900 modern-day individuals and 2,453 ancient individuals, including our newly reported data for 2–15 clusters *k* and display the results for *k* = 6, the lowest *k* to differentiate genetic clusters associated with three key ancestry components for Europeans, that is, Western hunter-gatherers (WHGs), early European farmers (EEFs), and Caucasus hunter-gatherers (CHGs) (Fig. 2*B* and *SI Appendix*, Fig. S10). We observe a separation of most of the individuals into distinct clusters in PC space that remain stable considering the 95% CI ellipses of low coverage samples and when constructing PCs on a worldwide and Eurasian set of modern-day individuals (*SI Appendix*, Figs. S11 and S12) and that correlate with the proportion of different components in ADMIXTURE analysis. We confirm these genetic clusters with pairwise *qpWave* analyses (*SI Appendix*, Fig. S13 and Tables S2–S7), group the individuals according to their genetic cluster and chronological and archaeological contexts, and perform subsequent ancestry modeling on a per-group and per-individual basis (*SI Appendix*, Tables S8–S17 and Dataset S5).

### The Civilian Population of Himera and Surrounding Regions.

We find that the IA Sicilians (Sicily_IA), associated with the Sicani culture, form a homogenous cluster distinct from most of the individuals excavated at Himera. They fall in the PC space occupied by earlier Middle and Late Bronze Age Sicilians (Sicily_MBA and Sicily_LBA; Fig. 2*A*) and display similar and homogenous proportions of the genetic components maximized in WHGs, CHGs, and EEFs in the ADMIXTURE analysis (blue, green, and orange in Fig. 2*B*, respectively). Using *qpAdm*, this group can be modeled as an admixture of four sources that distantly contributed to the genetic composition of Europeans (*P* = 0.179): Northwestern Anatolian Neolithic farmers (Turkey_Barcin_N; 76.4 ± 1.2%), WHGs (6.4 ± 1.0%), early farmers from Iran (Iran_GanjDareh_N; 6.3 ± 1.5%), and Early Bronze Age (EBA) Steppe herders associated with the Yamnaya cultural complex (Russia_Samara_EBA_Yamnaya; 10.9 ± 1.6%), which indicates an increase of Iranian-related admixture compared with the preceding LBA Sicily group, which can be best modeled without that component (*SI Appendix*, Table S8). Models using more proximal Bronze Age (BA) to Iron Age (IA) ancestry sources are not rejected for two-way mixtures between a local Sicilian source (Sicily_LBA, Sicily_LBA_I10371, or Sicily_MBA) on the one hand (57.1–98.7%) and Italy_IA_Republic.SG, Spain_BA, Spain_IA, Armenia_LBA.SG, or Balkan_IA (1.3–42.9%) on the other hand (*SI Appendix*, Table S9). This indicates that the population might not have been completely continuous to the BA inhabitants of Sicily sampled to date and instead received some gene flow from other populations that are most plausibly from outside of Sicily. We caution that there are only published data from 15 individuals from BA Sicily, leaving the possibility that some unsampled Sicilian populations were genetically more continuous with later groups. The potential connection to Spain_IA is intriguing, as structural similarities in EBA fortifications have been documented at Castelluccio near Syracuse and artifacts, such as Bell Beakers, have been found at BA sites near Palermo (51). The arrival of amber on Sicily from Iberia has been documented to at least the third century BCE (52), if not earlier (53). Additionally, laterally spiked axes have been discovered on Sicily that likely derive from Iberian metallurgical traditions, suggesting wide Mediterranean trade networks (54). However, Leighton (1999) notes that, although “it has often been supposed that Sicilian beakers derive from Spanish types…there is no sure evidence that they represent actual imports, and since North Italian finds have multiplied in recent years and a central European origin for the form now seems likely, the case for a direct Iberian link is not as compelling as it was” (9). Ancient historians, who plausibly had access to sources that have since been lost, also discussed the possibility of connections to Iberia: Thucydides wrote of the Sicani that they were not indigenous to the island but settlers from Iberia (Thuc. 6.2.2) (55), but Diodorus Siculus disagreed with this claim and instead name them as the original inhabitants of Sicily (Diod. 5.2.4). Of course, the accounts of ancient historians writing hundreds or even thousands of years after the events they describe should not be taken as direct evidence but only as a representation of even older oral history, which is important to consider but may not be based on fact at all. Our results offer tentative support to both scenarios; however, it is unlikely that the Sicani were descended only from Iberian or other non-Sicilian populations. One line of evidence for some local genetic continuity is the almost exclusive presence of Y-chromosomal haplogroup G-Z1903 and its derivates among the males (Dataset S6), a lineage already found among Sicilian inhabitants in the Middle Bronze Age (MBA) and LBA (12) and otherwise unreported from any pre- or early historic contexts, including Iberia. One male belongs to haplogroup R-FT40455, which is a specific subtype of haplogroup R-DF27, which could reflect an Iberian source of ancestry, as it is much more common in Iberia from the BA onward than in any other region (19, 56). R-DF27 has also been observed in individuals of the EBA in Sicily (12) and thus could have that persisted to the time of the Sicani culture. We find overall low levels of runs of homozygosity among the Sicily_IA and preceding BA individuals (*SI Appendix*, Fig. S14 and Table S18), providing no evidence for endogamous practices and pointing toward large effective population sizes (57).

We analyzed 11 individuals, believed to represent civilians, from single graves in Himera’s West necropolis, which was in use during the sixth to fifth centuries BCE and potentially spanning the entire occupation of the site, as analyses of burial inclusions to identify temporal information for individuals at Himera is still ongoing. Only three individuals have sufficient coverage (at least 50,000 SNPs covered) to perform formal statistics, but all individuals are consistent with a broadly Central or Eastern Mediterranean genetic profile in PC analysis (PCA) and ADMIXTURE (Fig. 2 and *SI Appendix*, Figs. S9–S12). Of the three higher-coverage individuals, both I20166/W3182 and I20168/W3702 are cladal with Greek_LBA (*P* = 3.55 × 10^−2^ and 7.80 × 10^−2^, respectively). I20163/W1838 appears distinct, with models of ancestry with the highest *P* values (*P* > 0.1) involving a contribution of 38–55% of his ancestry from a local Sicilian MBA, LBA, or IA source and around 45–62% from a group closely related to Punic individuals from Sardinia that harbor North African ancestry or alternatively around 87.6 ± 3.1% Sicily_IA ancestry with 12.4± 3.1% ancestry deriving from a group represented by genetic outlier from Chalcolithic Sardinia carrying fully North African ancestry (*SI Appendix*, Table S14). It is notable that four of the eight individuals that could be analyzed with ADMIXTURE carry a small proportion (2.2–3.8%) of a genetic component maximized in Africans that is not found to the same extent in our other Sicilian individuals but appears in Punic and Levantine individuals (Fig. 2*B*), and the same individuals are shifted toward Levantine groups on the worldwide PCA (*SI Appendix*, Fig. S11*B*). Considering the low coverage of the civilian individuals, comparisons of within-group variance and centroid distance on the first two PCs nevertheless suggest that the civilian sample overlapped with the soldiers of both battles in the groups Sicily_Himera_480BCE_1 and Sicily_Himera_409BCE, described below (*P* = 0.218, one-way PERMANOVA (58) of centroid distance). The Himera civilian individuals were, however, genetically significantly more diverse (*P* = 2.51–05, *t* test on Euclidean distance to the group centroid) than the soldiers, who derived most of their ancestry from Aegean-related populations and plausibly represented Greek settlers or their descendants. This greater genetic diversity is likely not simply explained by the lower coverage of the civilian individuals (*SI Appendix*, Figs. S12 and S15). A greater sample size ideally from a wider diversity of burial contexts at Himera would be necessary to test whether they could represent a genetically stratified subset of a more-diverse civilian population. Greek colonies were the meeting grounds of culturally and genetically diverse people (16, 17, 59). Those living in Greek poleis and *apoikiai* (independent cities and colonies, respectively) included people with ancestors from the Greek mainland and Greek isles but also people from the colony’s hinterlands and members of other cultural groups, such as Phoenicians and Etruscans, which could explain the signal we observe (2, 59–61). Himera, especially, may have had strong Phoenician ties due to its proximity to neighboring Punic settlements and the Himeran tyrant Terillus’ alliance with Carthage (Hdt. 7.165).

The individual buried at Himera’s East necropolis, which was in use from the seventh to fifth centuries, directly attests to the incorporation of local people into the populace of the colony, as this person is best modeled with *qpAdm* as deriving his ancestry from the contemporaneous Sicilian_IA group (*P* = 0.695). Potential social stratification has been suggested associated with the burial sites, as individuals interred in the East necropolis show lower δ^13^C and δ^15^N values and higher prevalence of skeletal pathology than those at the West necropolis, indicating a different access to resources (62). However, due to the limited number of samples, we cannot test whether such a significant stratification existed with respect to genetic ancestry.

### Diverse Ancestry among Himera’s Soldiers of the 480 BCE Battle.

Most Himerans associated with the battles can be found clustering on the PCA closely with individuals from the Greece_LBA, consistent with a major contribution of individuals of primarily Greek ancestry in the Himeran forces and substantial genetic continuity between the LBA period in Greece and fifth-century-BCE Greek colonies in Sicily. These soldiers with at least some Greek ancestry could have been inhabitants of the colony or supporting armies from other colonies, such as Syracuse. Seven of the 16 soldiers of the 480 BCE battle (Sicily_Himera_480BCE_1) and all 5 of the soldiers of the 409 BCE battle (Sicily_Himera_409BCE) are part of this main genetic cluster. Using the *qpWave/qpAdm* framework, we can model each of the soldiers in these two groups as deriving their ancestry either 100% from a group related to Greece_LBA or from an admixture between a Sicilian LBA or IA source and an Aegean-related source in varying proportions (*SI Appendix*, Tables S16 and S17), suggesting that many soldiers (and all studied from the 409 BCE battle) were plausibly the descendants of the Greek colonizers of Sicily and that intermarriage between Greeks and Sicilian locals was practiced (63). This genetic evidence adds to our understanding of social practices in Greek colonies in Sicily, because it is difficult to detect intermarriage archaeologically due to the fact that the presence of local Sicilian objects at Greek colonies may represent trade rather than intermarriage (59, 64, 65). Genetic data also provide information that may be invisible archaeologically, because individuals would have had the agency to decide which burial traditions and objects to adopt when interring their dead, and these archaeological identity markers, and the way individuals viewed their own identities, may or may not align with genetic ancestry (59, 66).

Beyond the intermarriage between locals and Himera’s Greek settlers, it is important to consider the possibility that the composition of the population might also have been influenced by a large-scale influx of Dorians from Agrigento after a political takeover by the Agrigentine tyrant, Theron, in 476 BCE, during which thousands of Himera’s inhabitants were killed (Diod. 11.49); however, the limited sample size available for analysis at this point and the lack of comparative data from Dorian contexts does not allow us to test this hypothesis. In any case, both groups of soldiers are heterogeneous in their admixture with local Sicilians, indicating a different genetic history for each individual; while we can compellingly document that significant genetic variation existed, a larger sample size than we have available would be necessary to adequately characterize the distribution of genetic variation encompassed by Sicilian Greek ethnicity in this period.

Among the soldiers of the 480 BCE battle, we find nine individuals that carry genetic ancestry not consistent with the first group (Fig. 2 and *SI Appendix*, Figs. S10–S13). We tested ancestry models with *qpAdm* for the higher coverage individuals.

Sicily_Himera_480BCE_2 consists of two outlying individuals (I10946/W1771 and I10950/W814) that fall on the PCA intermediate between the main cluster and central European individuals, a differentiation also indicated by the relatively higher proportion of the WHG genetic cluster in ADMIXTURE. Testing a wide range of possible BA and IA sources with *qpAdm*, valid models of ancestry consist of mixtures of one source related to central or eastern Mediterranean groups (Sicilian, Aegean, or Balkan) and one source related to central or western European groups (France, Spain, Czechia, or Hungary). A genetic origin in the Balkans is suggested by their Y chromosomes, belonging to the E-V13 lineage, which has its highest modern-day frequency in that region (67).

Two individuals (I10943/W0396 and I10949/W0403; Sicily_Himera_480BCE_3) fall with modern northeastern European groups and eastern Baltic populations of the first millennium BCE and can be modeled using exclusively BA individuals from Lithuania as a proxy source (*P* = 0.129).

One low-coverage individual, I17870/W0336, falls intermediate between Sicily_Himera_480BCE_2 and Sicily_Himera_480BCE_3 on both PCA and with respect to the main ancestry clusters inferred from ADMIXTURE (Fig. 2).

Two (I10944/W0461 and I10947/W1774; Sicily_Himera_480BCE_4) fall with individuals from IA nomadic contexts in the Eurasian Steppe and carry in the ADMIXTURE analysis genetic components maximized in Han and Karitiana (Native Americans) that characterize most IA Steppe nomads (light green and purple, respectively, in *SI Appendix*, Fig. S2*B*). In *qpAdm*, their ancestry is consistent, with around 85–89% deriving from IA Central steppe nomads and 11–15% from an Aegean-like source, an admixture that plausibly could have taken place among the genetically diverse populations of the Steppe (68, 69). Their mitochondrial DNA (mtDNA) haplogroups suggest east Eurasian genetic roots: A6a, found so far only in modern-day China (70, 71), and N1a1a1a, restricted to Russia, Kazakhstan, and Mongolia (72).

Finally, one outlier (I10951/W0653; Sicily_Himera_480BCE_5) falls with modern Caucasus populations and intermediate to ancient Steppe and Caucasus individuals on the PCA and carries the highest proportion of the CHG component in ADMIXTURE (Fig. 2). A single one-way model with a group closely related to Armenia_MBA as the source fit the data (*P* = 0.293). Similarly, the second low-coverage individual, I17872/W0428, falls closest to populations from the Caucasus on the PCA (Fig. 2*A*).

Outgroup-*f_3_* statistics with ancient groups reflect the same general patterns of genetic affinities (*SI Appendix*, Fig. S16), as do proportions of distal ancestry estimated with *qpAdm* (*SI Appendix*, Table S8 and Dataset S5). The groups can all be modeled as deriving differing proportions of ancestry from groups related to Turkey_N_Barcin, WHG, Iran_GanjDareh_N/CHG, and Russia_Samara_EBA_Yamnaya, except for group 4, which requires the addition of Siberian ancestry.

### Integration of Isotopic Evidence Supports Mercenary Presence in the 480 BCE Battle.

While the genetic makeup of a person gives insight into the origin of his or her ancestors, whether recently or many generations back in time, it does not provide information about the geographic region where the person lived or grew up. Strontium and oxygen isotope ratios reflect the local geology and hydrology of a person’s place of residence during the period of tooth or bone formation and provide information about individual mobility in a single lifespan (73). Thus, genetic and isotopic information provide complementary information about human mobility and life history.

**Fig. 3.**
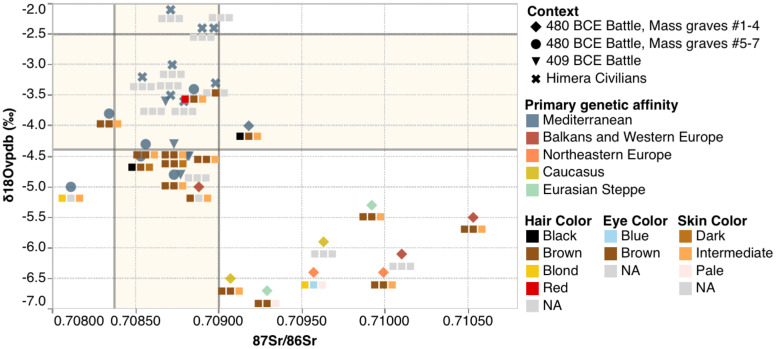
Summary of isotopic ratios, grave context, genetic affinities, and phenotypic information. Strontium and oxygen isotope data of the soldiers and civilians discussed in this paper. Symbols and symbol colors indicate grave context and genetic affinity, respectively. Associated colored squares indicate from left to right most probable hair, eye, and skin pigmentation (NA = not assessable). Shaded regions represent the local baselines of isotope values for Himera, measured from archaeological fauna in the case of strontium, and from *n* = 93 humans from Himera’s civilian population in the case of oxygen; see ref. (37) for details on geochemical data.

We correlated the diversity in genetic histories with strontium and oxygen isotope data gathered from the teeth of Himeran individuals in previous research (37). For all the individuals from the civilian sample, all the individuals from 409 BCE graves, and most of the individuals in the 480 BCE graves determined to be Aegean related (five of seven), the strontium isotopic ratios are consistent with local origins (37). In contrast, all 480 BCE soldiers with primary genetic affinities with central Europe, northeastern Europe, the Eurasian steppe, and Armenia are isotopically nonlocal (Fig. 3). The low oxygen ratios of some individuals are characteristic of higher latitudes, higher altitudes, cooler climates, greater rainfall, and greater distances from coasts than Himera, and the high strontium isotope ratios of some individuals indicate more ancient underlying rocks and soils than at Himera (37, 74). The outlying isotopic data by themselves do not prove non-Sicilian origins for the individuals from Himera: similar outlying ratios for both isotopes can also be found in other parts of Sicily, albeit not in the immediate region of Himera. However, the agreement between genetic and isotopic evidence, showing that all nine individuals with outlying genetic profiles also have nonlocal isotopic profiles, provide compelling evidence that many soldiers who fought in the battle of 480 BCE came from distant regions of the known Greek world.

These findings suggests the possibility that, unlike the genetically homogeneous sampled individuals who died in battle in 409 BCE, soldiers from 480 BCE had more diverse origins. This supports the interpretation that the Greek army in 480 BCE included mercenaries, or paid soldiers, that were of Greek and other cultural backgrounds (38). Both Greek colonies and other polities hired mercenaries to support citizen-based hoplite armies (6, 7, 75–77) (Thuc. 1.60: Greek use of Mercenaries; Hdt. 7.165: Diverse army led by Hamilcar the Carthaginian; Hdt. 8.26: Helping Persia; Hdt.2.152.4–5: Helping Egypt). It is well documented that Punic armies, such as Carthage, often used mercenary armies (8, 42), and it is likely that Greek tyrants on Sicily did the same (43). Although the ancient historians do not specifically mention mercenaries fighting for Himera in 480 BCE, they do mention that the Syracusan tyrant Gelon had gathered a large number of foreign mercenaries to round out his army (Diod. 11.72); these would plausibly have been among the relief forces sent to defend Himera. Sicily was a particularly attractive destination for mercenaries, as many Greek *poleis* on Sicily were governed by tyrants capable of extracting the kind of wealth needed to fund armies of mercenaries and usually in need of bodyguards to maintain their control (43, 77, 78). Some mercenaries reputedly were recruited from areas famed for particular skills, such as archers from Scythia (36) and peltasts from Thrace (79).

Soldiers from 480 BCE, whose genetic diversity exceeds that of the civilian sample at Himera, therefore likely did not reflect a draft exclusively from the Himeran polis, and the combination of genetic and isotope data suggest they were born and raised even further afield than Sicily itself. In contrast, the isotopic signatures that are mostly consistent with a local origin (37), and the genetic similarity of adult male individuals interred in mass graves associated with the battle of 409 BCE, corroborates historical accounts and suggests a fighting force drawn from Himera’s civilian population, without aid from mercenaries or allies. Historical accounts state that Syracusan Greeks initially were sent to aid Himera in 409 BCE but ultimately turned back in order to protect Syracuse, leaving Himera to fend for itself, relying on its own citizens (Diod. 13.59–61). However, as we only have genetic data for five of the soldiers of the 409 BCE battle, additional data would be required to confirm whether the lack of individuals with non-Aegean genetic origins is statistically significant.

### Identity Markers and Burial Treatment.

There are patterns in genetic affinities among the seven mass graves associated with the battle of 480 BCE. All the soldiers who fall outside the Aegean genetic cluster are interred in mass graves Nos. 1–4 (*SI Appendix*, Figs. S1–S3), whereas all individuals from mass graves Nos. 5–7 (*SI Appendix*, Figs. S4–S6) fall within this cluster, a statistically significant difference (*P* = 0.0028 by a χ^2^ test with one degree of freedom; Fig. 3). This result mirrors strontium isotope evidence showing more nonlocals interred in mass graves Nos. 1–4 (*P* = 0.0035 by a χ^2^ test with one degree of freedom; Fig. 3 and Dataset S2) (37). Mass graves Nos. 1–4 and Nos. 5–7 also are spatially segregated and differ in size, with mass graves Nos. 1–4 comprising significantly more interments (80) (*SI Appendix*, Figs. S1–S3). The correlation of burial location of individuals with their genetic ancestries suggests deliberate placement of soldiers with similar ancestral backgrounds in the same mass grave, reflecting the importance of identity in determining burial in this society (40, 59, 81).

These results suggest that individuals in mass graves Nos. 5–7 may have had greater prestige among survivors who buried them, consistent with previous arguments based on mortuary context (80). Individuals interred in mass graves Nos. 5–7 are significantly older than individuals interred in mass graves Nos. 1–4 (mean age, 45.0 versus 29.6 y old) and show no signs of having been dragged, as is the case for mass graves Nos. 1–4, or buried hastily, as is the case for mass graves Nos. 8/9 (80). Furthermore, individuals in mass graves Nos. 5 and 6 include grave goods, unlike the other mass graves from 480 BCE (32). The fact that these individuals also fall within the Aegean genetic cluster suggests a link between Aegean ancestry and prestige, as perceived by the individuals responsible for burying the fallen soldiers.

Constructions of ethnicity in the ancient Greek world were chiefly a matter of common descent and ancestral homelands but also were informed by cultural traits, such as language or material culture (82, 83). Our data show that the survivors who interred the casualties of the battle of 480 BCE incorporated information on the social origins of the deceased as part of their social logic for respectful treatment of the dead. Individuals with “foreign” ancestry, all of whom also are identified as nonlocal on the basis of isotopic evidence (Fig. 3 and Dataset S2), were interred in larger mass graves Nos. 1–4, and individuals with genetic affinities to other Greek populations were interred in the smaller mass graves Nos. 5–7. Nevertheless, it is not clear that nonlocals were considered as a homogeneous group of “others”. It is possible that the more-particular origins of the deceased were acknowledged and acted upon; for example, mass graves Nos. 1/2 contain both of the individuals with northeastern European ancestry. Identification of the fallen might have been done by surviving fellow soldiers who knew them, on the basis of their armor and weapons, or on the basis of phenotypic characteristics (Fig. 3 and *SI Appendix*, Table S19 and Dataset S7). Many other soldiers may have been buried in single interments, rendering them archaeologically invisible as soldiers. At Himera, some single interments do include individuals with perimortem trauma suggestive of being subjects of violence at the time of their deaths. These individuals could have died in battle and been interred in single burials by those who knew them. It is possible that some bodies were repatriated, as is documented in classical antiquity (84–86), with a greater likelihood of repatriation to locations elsewhere in Sicily than to more distant regions. Repatriation would have been achieved by first cremating the body (85, 86), and cremation was commonly practiced at Himera (87).

## Discussion

These results highlight how colonies facilitated migration, as reflected by heterogeneous ancestry deriving from geographically disparate locations. Furthermore, the results show how armed conflict served as a contact mechanism among diverse ancient populations in classical antiquity. In an already highly connected region, Greek warfare facilitated human mobility on a continental scale, providing a new line of evidence that enriches and supports scholarship emphasizing that migration occurred on a variety of scales, including small-scale movements of people (e.g., Horden and Purcell (88)), large-scale movements of groups (e.g., colonists; van Dommelen (89)), and globalization as an ancient process (90). Much of the migration related to warfare in the ancient Greek world may have been routine, small-scale movements of citizen soldiers (i.e., hoplites) (36), as had been documented in other contexts (91). However, our findings support suggestions that warfare, and the involvement of mercenaries, may have played an important role in large-scale movements of individuals in the ancient Greek world (7, 17). Greek mercenary warfare has been a focus of archaeological and historical research (6, 77, 92) but has rarely been discussed as a vehicle for culture change and gene flow in the context of Greek colonies (38), despite the recognized importance of warfare and the involvement of mercenaries as an ethnic unifying force in Phoenician contexts (8, 42, 93, 94). Like merchants, colonists, and slaves, mercenaries played a role in the transmission of culture in the Mediterranean region. While trade and colonization drew together communities around the Mediterranean region in person-to-person interactions (95), exchange of goods across vast geographic space did not necessitate migration and instead could be accomplished through intermediaries (i.e., merchants). Mercenaries were among the most long-distance travelers of the Greek world (rivaled, perhaps, by slaves), bringing persons of widely divergent cultural and genetic backgrounds face to face. Coupling genetic and isotopic evidence for mobility disambiguates the time scale of migration, indicating first generation migrants to Sicily. Mercenaries drawn to the Mediterranean region, and Sicily in particular, from distant regions of Europe, such as the Balkans, Gaul, and Germania, by the economic opportunity to serve Greek tyrants and participate in Greek wars were not merely itinerant “outsiders” in Greek poleis (96). They often lived within local communities, which deeply embedded them in alien cultures, as was the case for merchants and colonists, and rendered them powerful potential actors in cultural exchanges (38, 97). Historical evidence indicates instances of mercenaries becoming enfranchised as citizens and becoming integrated into the political, cultural, and genetic fabric of Greek poleis (75, 78). Diodorus Siculus writes of 7,000 foreign mercenaries in Syracusan tyrant Gelon’s employ (some of whom may have participated in the battle of 480 BCE) who were granted citizenship in 466 BCE (Diod. 11.72.3) (75). Besides Greek settlers and the indigenous peoples they encountered, mercenaries were part of the exchange of culture, ideas, and possibly genes taking place in Greek poleis in the first millennium BCE (38, 97) and might have, in some cases, played an important role in securing Greek military victories, as the divergent outcomes of Himera’s two battles demonstrate.

Historically contextualized genetic and isotopic evidence of foreign soldiers at Himera is another indicator of the heterogeneity and evolving complexity characterizing IA and post-IA societies of Europe, the Middle East, and other world regions (98). Identifying diverse origins of Classical Greek mercenaries provides nuance to our understanding of the long-distance movements of individuals relating to specialized economic or religious activities, apart from well-documented population movements, such as the establishment of colonies. Sites of the ancient Mediterranean for which genetic data have been published vary considerably in terms of settlement type, function, and historical context. The mass graves at Himera are singular in representing armed conflict and providing evidence for warfare as a dispersal mechanism of individuals across vast geographic space. By continuing to evaluate genetic trends as well as unique cases, in archaeological and historical context, research will be able to unravel series of different biographies of localities that, when combined, have the potential to enrich our understanding of trends, such as the intensification of trade, warfare, urbanism, and collapse.

## Materials and Methods

### Laboratory Processing.

We extracted tooth and bone powder from the samples excavated at Himera at the dedicated ancient DNA clean room facilities at the University of Vienna and Harvard Medical School according to the protocols described in refs. (99–101). Sampling for tooth and bone powder for the samples excavated at Polizzello and Baucina was carried out at the University of Florence. The bones and teeth were initially irradiated by ultraviolet light. We collected powder from the densest part of the inner ear as described in Pinhasi et al. (100) and from the tooth dentine using a dentist microdrill with disposable tips. At the University of Florence, we produced DNA extracts from the tooth samples from Baucina (102) and made a double-stranded and dual-indexed library without enzymatic damage repair (103, 104). All other samples were extracted at Harvard Medical School manually (99, 102) or with the assistance of robotic liquid handlers using silica-coated magnetic beads and Buffer D (105). We produced either dual-barcoded, double-stranded, high-throughput sequencing DNA libraries (45, 106, 107) or dual-indexed, single-stranded libraries (108) with custom oligonucleotide sequences as in ref. (109). All these extracts were pretreated prior to library preparation with a mixture of UDG and Endo VIII (USER, New England Biolabs) to reduce the cytosine-to-thymine damage characteristic of ancient DNA sequences in the interior of DNA fragments while retaining damage in both terminal bases (45). For some samples, we built several libraries (Dataset S1). We enriched the amplified libraries using two consecutive rounds of hybridization capture for sequences overlapping about 1.2 million nuclear targets (47, 48, 110) and the mitochondrial genome (111) (“1240K” enrichment) or a custom probe panel synthesized by Twist Biosciences, targeting the same 1.2 million targets and additional SNPs and tiling regions with mitochondrial probed spiked in (“Twist Ancient DNA” enrichment). Twist Ancient DNA enrichment involved one round of enrichment using reagents and buffers provided by Twist Biosciences, rather than two rounds as for 1240K (112). After indexing the enrichment products for double-stranded libraries only in a way that assigned a unique index combination to each library (104), we shotgun-sequenced libraries on an Illumina NextSeq500 instrument using v2 150 cycle kits for 2 × 76 cycles and 2 × 7 cycles (2 × 8 for single-stranded libraries) or a HiSeq X 10 using v2.5 kits for 2 × 100 cycles and 2 × 7 cycles (2 × 8 for single-stranded libraries) and sequenced the enriched products on an Illumina NextSeq500 instrument using v.2 150 cycle kits for 2 × 76 cycles and 2 × 7 cycles (2 × 8 for single-stranded libraries) or a HiSeq X 10 using v2.5 kits for 2 × 100 cycles and 2 × 7 cycles (or 2 × 8 for single-stranded libraries).

### Data Processing.

We processed the sequenced reads by trimming adapters and barcodes with SeqPrep (https://github.com/jstjohn/SeqPrep) or a custom script (https://github.com/DReichLab/ADNA-Tools) and merging paired-end reads that overlap in at least 15 bases (allowing one mismatch), taking the highest quality base in the merged segment to represent the allele. Using the *samse* command of BWA (version 0.6.1) (113), we mapped the resulting sequences to the human reference genome sequence *hg19* [GRCh37], and the sequences resulting from mtDNA capture to the mitochondrial reference genome *RSRS* (114). We filtered out sequences that overlapped known insertion/deletion polymorphisms and that have a mapping quality below 10 and a base quality below 20 and trimmed two nucleotides from the end of each sequence to reduce deamination artifacts. We obtained pseudohaploid SNP calls by randomly selecting a single sequence to represent each individual at each position covered by at least one sequence. We restricted all our analyses to 1,233,013 SNPs in common between 1240K and Twist Ancient DNA, as well as the mitochondrial genome. Individuals covered at 10,000 or more targeted SNPs were included in analyses.

### Analyses.

We assessed ancient DNA authenticity in each library by confirming that the rate of cytosine-to-thymine substitution in the first nucleotide occurred at a level consistent with genuine ancient DNA (115). We used *contamMix* to test for contamination based on polymorphism in the mtDNA (116), applying an mtDNA contamination threshold of 5%, and ANGSD to test for contamination based on polymorphism on the X chromosome in males (117), applying an X-chromosomal contamination threshold of 2%. We tested for pairs of related individuals using the software READ (118). We assigned mitochondrial haplogroups using *haplogrep2* (119). We assigned Y chromosomal haplogroups according to the phylogeny in Yfull 8.09 (https://www.yfull.com/chart/tree/) using all reads mapping to chromosome Y after trimming the read ends according to the UDG treatment. We determined the genetic sex of individuals by computing the ratio of the numbers of sequences mapping to the Y chromosome to the combined number of sequences mapping to the X and Y chromosomes (120).

We merged the dataset above with 3,291 modern-day individuals from 109 worldwide populations genotyped on the HO array, 96 newly reported in this study (Dataset S3). We used the *smartpca* function of EIGENSOFT (121) to perform PCA using default parameters, with the settings *lsqproject:YES* and *numoutlier:0*, using projection for ancient individuals. We performed clustering using unsupervised ADMIXTURE (50) for k = 2 to k = 15. We pruned SNPs in linkage disequilibrium with one another with PLINK using the parameter –indep-pairwise 200 25 0.4. Formal statistical analyses, such as outgroup-*f_3_* statistics, *qpfmv*, and ancestry estimation using the *qpWave/qpAdm* framework, were carried out using ADMIXTOOLS (110). For the latter analyses, we used the outgroups Mbuti.DG, Russia_Ust_Ishim_HG.DG, CHG, EHG, Spain_ElMiron, Czech_Vestonice16, Russia_MA1_HG.SG, Israel_Natufian, Jordan_PPNB, Morocco_EN.SG, and Russia_Shamanka_Eneolithic.SG.

### Data Generation for Newly Reported Modern Individuals.

We genotyped 96 present-day individuals from Greece and Italy on the HO SNP array (122) (Dataset S3) and applied quality control analyses as previously described (49). All individuals that were genotyped provided individual informed consent consistent with studies of population history, following protocols approved by the ethical review committees of the institutions of the researchers who collected the samples. For the newly reported Italian individuals (Dataset S3), biological samples were collected from healthy and unrelated subjects with all four grandparents born in the same region. DNAs were extracted from mouthwash with classical phenol/chloroform protocols, after a cell precipitation step. All participants gave informed consent to study protocols, which were reviewed and approved by the Ethics Committee of the University of Perugia (protocol no. 2017–01; for sample IDs starting with PG or VT) and by the Ethics Committee for Clinical Experimentation of the University of Pavia (board minutes of 11 April 2013; for the remaining samples). See ref. (123) for additional information.

## Supplementary Material

Supplementary File

Supplementary File

Supplementary File

Supplementary File

Supplementary File

Supplementary File

Supplementary File

Supplementary File

## Data Availability

Ancient DNA sequence data have been deposited in the European Nucleotide Archive (ENA, accession no. PRJEB55842, submitted Sept 7th 2022) (124).
