# Supplementary material for: The diverse genetic origins of a Classical period Greek army
Source: Proc Natl Acad Sci U S A. 2022 Oct 3;119(41):e2205272119. doi: 10.1073/pnas.2205272119 (PMC9564095; doi:10.1073/pnas.2205272119)
Supplement: Supplementary File [file pnas.2205272119.sapp.pdf]

PNAS

Supporting Information for

The diverse genetic origins of a Classical period Greek army.

Laurie J. Reitsema*†, Alissa Mittnik,*†, Britney Kyle*†, Giulio Catalano, Pier Francesco Fabbri, Adam C. S. Kazmi, Katherine L. Reinberger, Luca Sineo, Stefano Vassallo, Rebecca Bernardos, Nasreen Broomandskhoshbacht, Kim Callan, Francesca Candilio, Olivia Cheronet, Elizabeth Curtis, Daniel Fernandes, Martina Lari, Ann Marie Lawson, Matthew Mah, Swapan Mallick, Kirsten Mandl, Adam Micco, Alessandra Modi, Jonas Oppenheimer, Kadir Toykan Özdogan, Nadin Rohland, Kristin Stewardson, Stefania Vai, Chiara Vergata, J. Noah Workman, Fatma Zalzala, Valentina Zaro, Alessandro Achilli, Achilles Anagnostopoulos, Cristian Capelli, Varnavas Constantinou, Hovirag Lancioni, Anna Olivieri, Anastasia Papadopoulou, Nikoleta Psatha, Ornella Semino, John Stamatoyannopoulos, Ioanna Valliannou, Evangelia Yannaki, Iosif Lazaridis, Nick Patterson, Harald Ringbauer, David Caramelli*, Ron Pinhasi*, David Reich*

* Corresponding authors: Laurie J. Reitsema, Alissa Mittnik, Britney Kyle, David Caramelli, Ron Pinhasi, and David Reich

Email: reitsema@uga.edu, alissa_mittnik@hms.harvard.edu, britney.kyle@unco.edu, david.caramelli@unifi.it, ron.pinhasi@univie.ac.at, reich@genetics.med.harvard.edu
†These authors contributed equally to this work.

This PDF file includes:

Supporting text
Figures S1 to S16
Tables S1 to S19
Legends for Datasets S1 to S7
SI References

Other supporting materials for this manuscript include the following:

Datasets S1 to S7

Archaeological and Historical Context

Ancient writing on colonies, ethnicity, mercenaries and warfare in archaeological context

English translations of the passages are provided as links below and are derived from the LOEB standard editions. The following ancient authors are included here: Diodorus Siculus (46, 47, 48), Thucydides (49, 50), Herodotus (51, 52, 53), and Xenophon (54). Diodorus Siculus of Agyrium lived in the 1st c. BCE and published his 40-volume universal history in the mid-1st c. BCE (circa 59-56 BCE). Thucydides of Athens is the main primary source available for the Peloponnesian War, publishing his work in the late 5th c. BCE. Herodotus of Halicarnassus is regarded as the founder of history, publishing his work in the mid-5th c. BCE. Xenophon of Athens was born in the 430s BCE and published his works in the first quarter of the 4th c. BCE.

Section I: Ancient Inhabitants of Sicily; Zancle and Himera

Diod. 5.2.4

https://penelope.uchicago.edu/Thayer/E/Roman/Texts/Diodorus_Siculus/5A*.html

Thuc. 6.2.2

http://www.perseus.tufts.edu/hopper/text?doc=Perseus%3Atext%3A1999.01.0200%3Abook%3D6%3Achapter%3D2%3Asection%3D2

Thuc. 6.4.5

http://www.perseus.tufts.edu/hopper/text?doc=Thuc.+6.4.5&fromdoc=Perseus%3Atext%3A1999.01.0200

Thuc. 6.5.1

http://www.perseus.tufts.edu/hopper/text?doc=Thuc.+6.5.1&fromdoc=Perseus%3Atext%3A1999.01.0200

Diod. 11.49

http://www.perseus.tufts.edu/hopper/text?doc=Diod.+11.49&fromdoc=Perseus%3Atext%3A1999.01.0200

Section II: Battles of Himera, 480 and 409 BCE

Diod. 11.20-25

http://www.perseus.tufts.edu/hopper/text?doc=Perseus%3Atext%3A1999.01.0084%3Abook%3D11%3Achapter%3D25

Diod. 13.59-62

http://www.perseus.tufts.edu/hopper/text?doc=Perseus%3Atext%3A1999.01.0084%3Abook%3D13%3Achapter%3D62

Xen. Hell. 1.1.37

http://www.perseus.tufts.edu/hopper/text?doc=Xen.+Hell.+1.1.37&fromdoc=Perseus%3Atext%3A1999.01.0206

Section III: Mercenaries

Hdt. 2.152

http://www.perseus.tufts.edu/hopper/text?doc=Hdt.+2.152&fromdoc=Perseus%3Atext%3A1999.01.0206

Hdt. 7.165-7

http://www.perseus.tufts.edu/hopper/text?doc=Perseus%3Atext%3A1999.01.0126%3Abook%3D7%3Achapter%3D167

Hdt. 8.26, 8.52

http://www.perseus.tufts.edu/hopper/text?doc=Hdt.+8.52&fromdoc=Perseus%3Atext%3A1999.01.0126

Diod. 11.72

http://www.perseus.tufts.edu/hopper/text?doc=Diod.+11.72&fromdoc=Perseus%3Atext%3A1999.01.0126

Thuc. 1.60

http://www.perseus.tufts.edu/hopper/text?doc=Thuc.+1.60&fromdoc=Perseus%3Atext%3A1999.01.0084

Site descriptions

Himera, Sicily, Italy (⨁ 37.972, 13.825)

Laurie Reitsema, Britney Kyle

Greek colonies were established across the Mediterranean region beginning in the 8th century BCE (1). On Sicily, Greek's established colonies (*poleis*) along the eastern and southern coasts, with Himera the only colony established on the northern coast, while Phoenicians established *emporia* on the western side (2-4). Himera was founded in 648 BCE by culturally Greek individuals from Zancle (modern day Messina), which itself was a colony founded by Chalcidians from the island of Euboea in the late 8th century BCE (5-8)(Herodotus 6.24; Strabo 6.2). Settlers from Syracuse, a Corinthian Greek colony, and possibly individuals from a third unknown group referenced by Thucydides, may also have contributed to the establishment of Himera (9). Himera was an important trade port located on the Tyrrhenian Sea coast and formed the northern- and western-most boundary of Greek control in Sicily (5, 10) (7). It is clear from coins and ceramics that Himera was well connected with foreign cities through trade (6, 7, 11-15).

Two historically documented battles took place at Himera, in 480 and 409 BCE (10). In both cases, Carthaginian Phoenicians attacked Himera. Leading into the 5th century BCE, Himerans were generally cooperative with neighboring Phoenicians, with evidence of trade between the two groups and with the neighboring indigenous Sicilians, the Sicani (6, 16). However, by the 5th century BCE,

Greek tyrannies became increasingly prominent on Sicily, and a division emerged between Greek *poleis* opposed to, and allied with, Phoenicians; specifically, Carthage (17, 18). In the early 5th century, the tyrant of Himera, Terillus, opted for alliance with Carthage. When Terillus was ousted by Himerans in 483 BCE (Herodotus 7.165), Carthage agreed to aid him in regaining control, and in 480 BCE, dispatched a large force to retake the city. Faced with the threat of Carthaginian invasion in 480 BCE, Himerans successfully rallied outside forces, with specific mentions of soldiers coming from Syracuse and Agrigento (Herodotus 7.165-166; Diodorus 11.20-21). This Greek alliance was successful in defending Himera in 480 BCE (Diod. 11.22; 10, 19).

An act of revenge, Carthage returned to Himera in 409 BCE with a large mercenary army (Diod. 13.59; (15, 20). Although Syracuse dispatched a relief force, a small number of whom aided Himera early in the battle, most Syracusan soldiers turned back out of concern for their own city's defense (Diod. 13.59-61; Xen. Hel. 1.1). Many citizens of Himera evacuated, and the remainder unsuccessfully attempted to defend their city (10) (Diod. 13.62; Xen. Hel. 1.1) (15, 19). The Greek loss in the Battle of Himera in 409 BCE marked the destruction and abandonment of the *polis* (Diod. 13.62) (10).

Until recently, knowledge of the two Battles of Himera came from historical documents, many of which were written hundreds of years after they occurred (e.g., Diod. 11.20-25, 13.62). In 2009, excavations of a necropolis near the ancient city of Himera revealed a series of mass graves that are likely associated with the Battles of Himera (15, 21, 22).

There were three cemeteries in use at the site of Himera: the East, the West, and the South (which remains unexcavated). The East necropolis was in use for the entire occupation of the colony, whereas the West necropolis was only in use during the 6th and 5th centuries BCE (23). Preliminary analyses of differences between the East and West necropoleis indicate that individuals interred in the East necropolis had lower δ13C and δ15N values and higher prevalence of skeletal pathology, suggesting that individuals in the East necropolis may have had differential access to resources, likely as a result of lower social status or due to changing access to resources through time (24).

Except for one individual excavated at the East necropolis, all skeletal remains included in this study are all from the West necropolis of Himera, including single interments of members of the civilian population, and multiple interments of individuals interpreted as casualties of the ancient battles in 480 BCE and 409 BCE (15)(Table S1). Mass graves #1-7 are associated with the Battle of 480 BCE. Objects from within the graves, and in other burials disturbed by the mass graves, place mass graves #1-7 in the late 6th-early 5th centuries. Mass grave #8/9, a continuous, large interment that represents a single mass burial (22), is associated with the Battle of 409 BCE and contains objects dating to the 5th century. Mass graves #1-7 are consistently orderly with bodies in extended, supine position on an East-West axis. They range in size from 18 bodies (grave #1/2, which came to be recognized as a single interment after excavation had begun), to just two bodies (grave #7). In contrast, mass grave #8/9, which contains 69 bodies associated with the 409 BCE battle, is less orderly, with bodies in multiple layers and positioned to fit the grave rather than vice versa, suggesting hastier burial. Evidence suggesting the mass graves are casualties from the Battles of Himera and not, for example, victims of epidemics, include the fact that they comprise young- to mid-aged adult males, many have signs of perimortem trauma, including weapon wounds, and weapon points are embedded in some of the bodies (22). Their deliberate burial in the Greek city's own necropolis suggests these individuals are Greek, and not enemy, casualties (15). Retrieval and burial of corpses was of paramount social and religious importance in Greek society at this time (25). We know nothing of the manner in which members of the Carthaginian army were buried, but it was typical in Greek warfare for the victor to allow the enemy access to the battlefield to remove its dead (25, 26).

Table S1. Distributions of skeletons across graves in Himera's West necropolis

Grave Number	Number of individuals, total	Number studied in 2016 by BK and LJR	Number in DNA study	% of the total skeletons in grave	% of the total seen by LJR/BK	(See footnote)
1/2	18	18	4	22%	22%	
3	22	19	2	9%	11%	
4	15	8	2	13%	25%	
5	4	4	2	50%	50%	
6	3	3	2	67%	67%	
7	2	2	2	100%	100%	
8/9	69	9	5	7%	56%	6%

*Mass graves 8/9: 69 skeletons recovered in mass grave 8/9 but 88 bodies estimated based on taphonomic analysis: 5/88=6%

Mass graves #1-4 and #5-7 are spatially segregated in Himera's West necropolis, forming two clusters in the western portion. All are along a N-S axis, with bodies oriented E-W. Mass graves #1/2, 3 and 4 each contain more than 10 individuals, whereas mass graves #5-7 each contain fewer than 10 individuals. Individuals in mass graves #1-4 tend to have partially abducted right humeri, evidence of having been dragged, whereas individuals in mass graves #5-7 do not (*36*). The individuals in mass graves #1-4 are also significantly younger than those in mass graves #5-7, and mass graves #5-6 contain grave goods, unlike the other 480 BCE graves (22). Mass grave #8/9 is the largest and densest of the mass graves, containing 69 individuals (27). Unusually for the necropolis, mass grave #8/9 is laid out on an E-W axis, and bodies within it are oriented in numerous positions, of which N-S is most common. An effort was made to sample more than one individual from every mass grave. Individuals were selected for analysis chiefly on the basis of skeletal completeness, prioritizing partial skeletons over highly fragmented remains. In 2016, only petrous pyramids were collected. If a petrous pyramid was identified separated from the rest of the skull, the entire petrous pyramid was collected. In 2017 the sampling strategy expanded to include teeth, which are more numerous than petrous pyramids at Himera. Teeth were chosen based on presence of intact enamel crowns and of roots, and presence of a remaining antimere (Datasets S1 and S2).

Sixty-three individuals from the mass graves were studied by the Bioarchaeology of Mediterranean Colonies Project in 2016 including demographic and paleopathological analyses. The 70 individuals that were not studied osteologically in 2016 almost always were represented by only poorly preserved bones or fragments that were not observable for skeletal stress markers. Of 63 individuals studied in 2016, approximately one-third had less than 15% of cranial bones present and three had no teeth. Therefore, the sample of soldiers included here (n=19) makes up approximately 14% of the total number of individuals documented in the mass graves (n=133), but approximately 30% of the skeletons available for osteological, isotopic and genetic analysis with sufficient cranial or dental remains. Although the number of individuals analyzed from every mass grave associated with 480 BCE is the same – two from each, excepting mass grave #1/2 for which four individuals were sampled – representativeness is higher for mass graves #5-7 by virtue of their smaller sizes (fewer than 10 individuals in each); for example, mass grave #3 contains 22 individuals and only two were genetically analyzed (9%), while mass grave #7 contains two individuals and both were analyzed (100%). The five individuals genetically analyzed from mass grave #8/9, associated with 409 BCE, represent approximately 7% of the total number of individuals (n=69 with identifiable remains and n=88 estimated taphonomically) and 55% of the total number studied osteologically in 2016. Members of the civilian sample were selected prioritizing partial skeletons over highly fragmented remains, and in an effort to include individuals buried in each of

the two most common body positions at Himera: supine and flexed. In Sicily, it is unlikely that the two different body positions reflect ethnic identity and ancestry (see discussion in Shepherd (28)), but genetic evidence is lacking. Approximately 10,000 skeletons have been excavated from the West necropolis at Himera, meaning the 11 individuals from the civilian sample reported here are a preliminary, and not a representative, estimation of the genetic diversity of the population.

The following individuals from Himera provided genome-wide data –

Attributed to the 480 BCE Battle of Himera:

W0396 (I10943). Petrous bone; young adult male

W0461 (I10944). Petrous bone; young adult male

W0494 (I10945). Petrous bone; young adult male

W1771 (I10946). Petrous bone; young adult male

W1774 (I10947). Petrous bone; young adult male

W2587 (I10948). Petrous bone; young adult male

W0403 (I10949). Petrous bone; young adult male with enthesopathy on proximal ulna

W0814 (I10950). Petrous bone; young adult male

W0653 (I10951). Petrous bone; young to mid-aged adult male

W2738 (I10952). Petrous bone; young adult male

W2590 (I7217). Petrous bone; young adult male with antemortem trauma of left distal tibial diaphysis

W2737 (I7218). Petrous bone; young adult male

W2764 (I7219). Petrous bone; young adult male with spear in chest; perimortem humeral trauma

W2825 (I7221). Petrous bone; young to mid-aged adult male with antemortem trauma to left tibia and right clavicle

W0428 (I17872). Tooth; young adult male

W0336 (I17870). Tooth; young adult male with spearhead found in abdomen

Attributed to the 409 BCE Battle of Himera:

W4380 (I17866). Tooth; young adult male

W4666 (I17884). Tooth; adult male

W4378 (I7223). Petrous bone; mid-aged adult male

W4651 (I7224). Petrous bone; young adult male

W4670 (I7225). Petrous bone; young adult male

Attributed to the civilian population of Himera:

W1153 (I17878). Tooth; mid-aged adult male

W2073 (I17879). Tooth; young adult female

W2499 (I17881). Tooth; young adult female

W2309 (I17887). Tooth; young adult female

W1480 (I20160). Tooth; male

W1656 (I20161). Tooth; female

W1788 (I20162). Tooth; male

W1838 (I20163). Tooth; male

W3182 (I20166). Tooth; male

W3612 (I20167). Tooth; female

W3702 (I20168). Tooth; male

Interred at the East necropolis of Himera:

RO1025-B (I17432). Petrous bone; mid-aged adult male interred in a double burial with another mid-aged adult male

Polizzello, Sicily, Italy (⊕ 37.608, 13.834)

Giulio Catalano and Luca Sineo

The sample of natives of Polizzello and Baucina (described below), respectively 6th-5th century BCE (Hellenized) and Iron Age, serve as a comparison to understand, with respect to the population of Himera, the indigenous size and the level of variability of the population. The site of Polizzello is located on a mountain 800 meters high just a short distance from Mussomeli (CL) in the central interior of Sicily. Archaeological excavations, started in the 1920s, have provided a long prehistoric and historic sequence from the Early Bronze Age to Medieval period (29). The human remains analyzed here were recovered in four rock-cut tombs excavated in 2004 by the Soprintendenza ai Beni Culturali e Ambientali of Caltanissetta. Typological analyses of burial style and grave inclusions indicate that the skeletal sample is attributed to the Iron Age period (900-700 BCE) when Polizzello was one of the most important indigenous Sicanian settlement in central-south Sicily(29). According to scholars, the site of Polizzello could be considered a proto-urban center characterized by a strong pastoral component. Archaeological surveys have attested the presence of Aegean trace in handicrafts and funerary architecture supporting the existence of contacts with Aegean-Cyprian cultures. Moreover, elements of Phoenicans derivation were also found. Together with these exotic features, Polizzello also developed a local production that seems to derive from previous Sicanian elements found in Mid-Late Bronze Age cultural phases of Thapsos and North

Pantalica. The peculiar tradition found in Polizzello seems also the result of impermeability of Sicanian settlements of central-south Sicily to the penetration of Siculo-Ausonian and Italic elements (30). With the emergence of extensive contacts with Greek colonies probably started around the early 7th century BCE, a process of acculturation took place in western Sicily and the cultural uniqueness of Sicanian settlements was contaminated by Greek components.

Based on anthropological studies, the presence of at least 224 individuals was estimated, including adults and infants of both sexes. Palaeopathological analyses have revealed high percentages of cribra variants and dental enamel hypoplasia (31).

The osteological collection of Polizzello is housed in the Museum of Mussomeli (Caltanissetta).

Genome-wide data in this study came from 19 petrous bones:

POL1 (I13376). Petrous bone; adult female

POL3 (I13377). Petrous bone; adult female

POL4 (I13378). Petrous bone; adult male

POL5 (I13379). Petrous bone; adult female

POL6 (I13380). Petrous bone; adult female

POL7 (I13381). Petrous bone; adult female

POL10 (I13382). Petrous bone; adult male

POL12 (I13383). Petrous bone; adult male

POL14 (I13384). Petrous bone; adult female

POL15 (I13385). Petrous bone; adult male

POL17 (I13386). Petrous bone; adult male

POL18 (I13387). Petrous bone; adult female

POL22 (I13389). Petrous bone; adult male

POL25 (I13390). Petrous bone; adult female

POL26 (I13391). Petrous bone; adult male

POL27 (I13392). Petrous bone; adult female

POL28 (I13393). Petrous bone; adult female

POL29 (I13394). Petrous bone; adult female

POL30 (I13395). Petrous bone; adult female

Baucina, Sicily, Italy (⨁ 37.925, 13.536)

Giulio Catalano, Luca Sineo and Britney Kyle

Together with Polizzelo, Baucina was chosen as a comparative sample for Himera because these are two of the most significant sites from which quantitative information (number of individuals) of ancient indigenous people was available. The necropolis is located on the Monte Falcone mountain (695 meters) near the modern town of Baucina. The site, excavated by the Soprintendenza and the University of Palermo several times, was a vast indigenous center between the 6th century and above all the 5th century BCE (32). Although dates for the skeletal assemblage at Baucina are not certain, with the exception of a few pieces of prehistoric pottery fragments and pieces of obsidian, the finds from the site all date to the 6th-5th c. BCE (33). Furthermore, the presence of Greek colonial pottery from the end of the 6th century BCE indicates contact with Greek settlements, likely Himera due to its proximity, and the presence of Punic amphora suggest commercial relations with nearby Solunto and Palermo. Thus, "Punic, Greek and indigenous traditions [are thought to have] coexisted" at the site, making it an important location to examine relationships between these groups (32). Simple burials and collective tombs of the type known as a 'grotticella' (chamber tombs) also show significant influences by the culture of the neighbouring Greek colony of Himera. The human skeletal remains found in the necropolis are estimated to derive from at least 50 individuals.

The samples providing genome-wide date are:

BAU_3 (I13125). Tooth; adult male

BAU_6 (I13128). Tooth; adult male

Population genetic analyses

We compiled a reference dataset consisting of whole genome data from 2,673 ancient individuals (Dataset S4) (https://reich.hms.harvard.edu/allen-ancient-dna-resource-aadr-downloadable-genotypes-present-day-and-ancient-dna-data), as well as previously reported whole-genome sequencing data from 346 worldwide modern-day individuals (34-37) (Dataset S4), and merged the pseudo-haploid genotypes from the Himera samples with this dataset.

We use individuals with fewer than 50,000 1240K SNPs covered in exploratory analyses and in group-based analyses, but do not individually formally model them.

Exploratory analyses

We tested for pairs of related individuals using the software READ (38), and found that in our dataset only two IA individuals from Polizzello (I13378 and I13394) share a likely 2nd degree relationship (Fig. S8). ANOVA between the different groups show significant differences in the variances of P0 (p=3.22E-8), and multiple one-tailed T-tests confirm this is due to the significantly lower mean P0 in the Sicily_IA group (Fig. S8B). This might suggest greater genetic homogeneity in this group compared to the inhabitants and combatants of Himera.

We merged the dataset above with 3291 modern-day individuals from 109 worldwide populations genotyped on the Affymentrix Human Origins (HO) array, 96 newly reported in this study (Dataset S3). We used the *smartpca* function of EIGENSOFT (39) to perform principal component analysis (PCA) using default parameters, with the settings *lsqproject:YES* and *numoutlier:0*. We projected the ancient individuals onto a PCA plot of 1196 modern-day West Eurasian individuals (40), 1900

modern-day worldwide individuals, and 1390 Eurasian individuals, restricting to the HO dataset of 597,573 SNPs (Figs. 2A and S9).

Most samples plot in PCA space together with Aegean and Sicilian LBA individuals with the remarkable exception of seven soldiers of the 480 BCE battle that plot together with Central Europeans, Northeastern Europeans, IA Steppe nomads and Caucasian populations, respectively. For the 10 low-coverage samples whose data intersects with fewer than 25,000 HO SNPs, 8 attributed to the civilian population of Himera and 2 480 BCE soldiers, we additionally plot ellipses representing the 95% jackknife confidence region (Fig. S12). The civilian samples overlap in their confidence distribution with individuals from the BA Aegean, Sicily_IA and Sardinian Punic individuals, and are inconsistent with populations from Western and Northern Europe, the Eurasian Steppes, the Caucasus, Near East or North Africa, suggesting that the genetic heterogeneity seen in the soldiers from the 480 BCE battle is due to their specific military context and not an effect of them being drafted from an already extremely heterogeneous local Himeran population. More subtle genetic heterogeneity in the Himeran civilian sample is nevertheless hinted at by the two higher coverage samples I20163, I20168 and I20166 whose 95% CI regions do not overlap, with the former two clustering more closely with Sicily_IA and the latter more closely with the BA Aegean. The 95% CI region of the low-coverage individuals I17870 and I17872 from the 480 BCE battle is consistent with modern-day individuals from Central Europe and the Caucasus, respectively.

We performed clustering using unsupervised ADMIXTURE (41), using all samples used in the worldwide PCA and ancient reference samples that overlap more than 50,000 HO SNPs. We pruned SNPs in linkage disequilibrium with one another with PLINK using the parameter --indep-pairwise 200 25 0.4, which left us with 282,184 SNPs. We performed an ADMIXTURE analysis for values of k between 2 and 15, and carried out 5 replicates at each value of *k*. We retained the highest likelihood replicate at each *k* and mainly discuss results for $k = 6$, as it serves to visually discriminate the relevant ancestral components (Figs. 2B and S10).

Most individuals exhibit at k=6 three major components, maximized in WHG, EEF and CHG, respectively, with the proportions qualitatively similar to that seen in the PCA. Notably, the two individuals that cluster with IA Steppe nomads have two additional components, maximized in Han and Karitiana; these components also appear in almost all Steppe nomads in highly variable proportions. Some of the lower coverage civilian genomes exhibit a small proportion of the component maximized in the southern African Ju|'hoan which is also found as a minor component in the Punic individuals from Sardinia and Ibiza as well as Eastern Mediterranean and North African individuals. This might point towards some genetic influence through contact with the Phoenician/Punic sphere, although in principle it could also reflect statistical noise due to the lower coverage of these individuals. In the higher coverage civilian individuals this component also appears in I20163 (although not I20166 or I20168), increasing confidence that this is a true signal and not just an artefact of low coverage.

Genetic clustering and outlier detection

To determine suitable analysis groupings and assess genetic homogeneity within and between groups of individuals from the same chronological and archaeological contexts, we used the *qpWave* tool in ADMIXTOOLS *(42)*, with default parameters and *allsnps:*YES (which calls upon the tool *qpfstats* which precomputes the expected value of *f*-statistics based on the overlap of all covered SNPs in a test population with the base population, (https://github.com/DReichLab/AdmixTools/blob/master/qpfs.pdf), testing individuals pairwise against each other to determine whether they were consistent with being a clade relative to 17 'Right' outgroups (Mbuti.DG, Russia_Ust_Ishim_HG.DG, CHG, EHG, Spain_ElMiron, Czech_Vestonice16, Russia_MA1_HG.SG, Israel_Natufian, Jordan_PPNB, Turkey_N_Barcin, WHG, Iran_GanjDareh_N, Russia_Samara_EBA_Yamnaya, Morocco_EN.SG, Greece_Crete_BA, Greece_LBA and Russia_Shamanka_Eneolithic.SG). The results are shown in Fig. S13; we

consider models with a p-value <0.01 as rejected. We confirm that cladality is not rejected for most pairs of individuals within a chronological and archaeological grouping, with the notable exception of 5 subgroups that can be identified within the soldiers of the 480 BCE battle. These subgroupings mirror genetic differentiation also seen in PCA and ADMIXTURE analyses. Genetic heterogeneity is furthermore evident within groups, as some pairwise models are rejected.

We then tested each individual for which pairwise *qpWave* tests with the others was rejected against the rest of the individuals grouped together, and treat the individual as a genetic outlier if this model is rejected, an approach to outlier detection previously used by Fernandes*, et al.* (43). We use 16 reference populations (P16) Mbuti.DG, Russia_Ust_Ishim_HG.DG, CHG, EHG, Spain_ElMiron, Czech_Vestonice16, Russia_MA1_HG.SG, Israel_Natufian, Jordan_PPNB, Turkey_N_Barcin, WHG, Iran_GanjDareh_N, Russia_Samara_EBA_Yamnaya, Morocco_EN.SG, Greece_Crete_BA and Russia_Shamanka_Eneolithic.SG, adding Greece_LBA in tests of groupings that chronologically post-date them. As *f*-statistics computed by *qpfstats* are expected to differ somewhat from the direct computation and we chose a different set of outgroup populations, we obtain in some cases different results here from those that have previously been published.

We additionally use the program *qpfmv*, which is a specialized implementation of *qpWave* that tests if every pair of samples I_i, I_j within an chronologically and archaeologically defined grouping is a clade with respect to the set of populations P_k, asking if all the pairwise f_4 statistics $f_4(I_i, I_j; O, P_k)$ are 0 up to noise, where O is an outgroup. In our tests we used Mbuti.DG as the outgroup and as reference populations the rest of the populations used with *qpWave*. *qpfmv* tests for rejection of the null hypothesis of homogeneity using a Hotelling T^2 statistic. This test indicates whether there remains any subtle genetic inhomogeneity in the grouping, even after outlier removal.

For the purpose of defining source populations in subsequent analyses, we reanalyze 17 previously published individuals (43): 11 individuals dated to the EBA, 2 from the MBA and 4 from the LBA:

Sicily_EBA

The *qpfmv* test (P16) rejects genetic homogeneity among all samples with a p-value <<0.01. In pairwise *qpWave* models based on the *qpfstats* output, using the same outgroups as in the original *qpWave* test without the chronologically later Greece_LBA, we identify 3 potential outliers for which cladality is rejected with other individuals (I3124, I8561, I11443; Table S2). We reran the test, grouping the other eight samples that formed a clade. In this test, cladality to the main grouping was not rejected for I3124 (p=0.058). Cladality was strongly rejected for I8561 and I11443 (p=14.3E-14 and 1.5E-42, respectively). We therefore treat these two individuals as genetic outliers from the Sicily_EBA group. Repeating the *qpfmv* analysis on the remaining group, homogeneity is rejected (p=6.4E-04), suggesting an inhomogeneous population overall which is consistent with the variation seen on PCA and ADMIXTURE and recent admixture.

Table S2. P-values for the *qpWave* models between Sicily_EBA individuals. Rejected models with p<0.01 marked in red.

	I11442	I11443	I3122	I3123	I3124	I7774_d	I7796	I7800	I7805	I7807	I8561	number of rejected tests
**I11442**		4.04E-29	5.63E-01	6.14E-02	1.32E-01	7.12E-01	6.32E-01	5.41E-01	4.50E-01	2.43E-02	1.90E-11	2
**I11443**	4.04E-29		4.28E-38	8.98E-15	6.99E-20	8.04E-09	2.11E-29	2.76E-11	9.66E-18	2.22E-33	4.73E-08	10
**I3122**	5.63E-01	4.28E-38		7.91E-02	4.05E-03	6.64E-01	8.04E-01	6.71E-01	6.23E-01	4.45E-01	9.46E-13	3
**I3123**	6.14E-02	8.98E-15	7.91E-02		7.08E-01	3.67E-01	4.67E-02	8.12E-01	4.65E-01	2.31E-02	8.32E-05	2
**I3124**	1.32E-01	6.99E-20	4.05E-03	7.08E-01		1.71E-01	6.72E-02	8.76E-01	4.41E-01	2.42E-03	1.70E-06	4
**I7774_d**	7.12E-01	8.04E-09	6.64E-01	3.67E-01	1.71E-01		5.95E-01	7.26E-01	6.32E-01	6.83E-01	8.25E-03	2
**I7796**	6.32E-01	2.11E-29	8.04E-01	4.67E-02	6.72E-02	5.95E-01		5.54E-01	6.35E-01	6.26E-01	2.29E-12	2
**I7800**	5.41E-01	2.76E-11	6.71E-01	8.12E-01	8.76E-01	7.26E-01	5.54E-01		9.64E-01	5.78E-01	3.97E-03	2
**I7805**	4.50E-01	9.66E-18	6.23E-01	4.65E-01	4.41E-01	6.32E-01	6.35E-01	9.64E-01		7.26E-01	1.62E-04	2
**I7807**	2.43E-02	2.22E-33	4.45E-01	2.31E-02	2.42E-03	6.83E-01	6.26E-01	5.78E-01	7.26E-01		2.06E-12	3
**I8561**	1.90E-11	4.73E-08	9.46E-13	8.32E-05	1.70E-06	8.25E-03	2.29E-12	3.97E-03	1.62E-04	2.06E-12		10

Sicily_MBA

In pairwise *qpWave* models (P16), we cannot reject cladality between the two samples (p=0.868). The *qpfmv* test cannot reject genetic homogeneity (p=0.8) and we group both individuals in further analyses.

Sicily_LBA

In the *qpWave* test (P16+Greece_LBA), we detect one sample I10371 for which pairwise *qpWave* models result in rejection (Table S3). Comparing this sample against the group of other three samples that form a clade, we find that cladality is still rejected and this individual represents a genetic outlier (p=1.85E-03). Before outlier removal, the *qpfmv* test rejects genetic homogeneity with p=1.01E-04. After removing I10371, we cannot reject homogeneity for the remaining 3 samples (p=0.077). We therefore treat I10371 as an outlier.

Table S3. P-values for the *qpWave* models between Sicily_LBA individuals. Rejected models with p<0.01 marked in red.

	I10371	I10372	I3876	I3878	number of rejected tests
**I10371**		4.02E-04	7.89E-04	4.87E-03	3
**I10372**	4.02E-04		2.50E-02	2.66E-01	1
**I3876**	7.89E-04	2.50E-02		4.98E-01	1
**I3878**	4.87E-03	2.66E-01	4.98E-01		1

For the following groups we report new data:

Sicily_IA

This group comprises 21 individuals from two Sicilian IA sites. They cluster closely together on PCA space also occupied by most Sicilian MBA and LBA individuals (Figs. 2A and S11), and are similar in their proportions of genetic components determined by ADMIXTURE (Fig. 2B). Two individuals (I13125 and I13382) have evidence of genetic heterogeneity in that pairwise *qpWave* models (P16+Greece_LBA) with respectively two or more other individuals in this cluster are rejected (p-values <0.007; Table S4). When we model these individuals as clades with the combined groups of the other individuals, the models for I13125 and I13382 are not rejected (p=0.0325 and 0.0165, respectively) and so we subsequently group all Sicily_IA individuals. However, the *qpfmv* test rejects genetic homogeneity among all samples with a p-value <<0.01, and the result remains after removing the two potential outliers. This suggests the presence of

subtle genetic differences between the samples, that *qpfmv* is able to pick up due to joint analysis of all the individuals.

Table S4. P-values for the *qpWave* models for pairwise combinations of Sicily_IA individuals. Rejected models with p<0.01 marked in red.

	I13395	I13394	I13393	I13392	I13391	I13390	I13389	I13387	I13386	I13385	I13384	I13383	I13382	I13381	I13380	I13379	I13378	I13377	I13376	I13128	I13125	number of rejected tests
**I13395**		8.47E-01	9.76E-01	8.65E-01	8.94E-01	2.92E-01	9.83E-01	4.63E-01	9.46E-01	6.58E-01	4.60E-01	7.86E-01	3.54E-01	1.02E-01	8.34E-01	8.20E-01	8.37E-01	5.32E-01	3.06E-01	7.46E-01	2.76E-02	0
**I13394**	8.47E-01		5.18E-01	4.83E-01	1.60E-01	8.38E-02	5.94E-01	5.18E-02	6.39E-01	7.67E-01	6.85E-01	7.39E-01	9.36E-02	2.84E-01	7.44E-01	1.91E-01	6.07E-01	9.76E-01	1.22E-01	6.37E-01	3.12E-01	0
**I13393**	9.76E-01	5.18E-01		2.88E-01	6.44E-01	1.83E-01	9.97E-01	1.80E-02	6.85E-01	4.73E-01	4.44E-01	4.13E-01	1.19E-02	4.63E-02	6.29E-01	2.17E-01	5.83E-01	2.85E-01	6.74E-02	5.46E-01	3.31E-02	0
**I13392**	8.65E-01	4.83E-01	2.88E-01		1.78E-01	1.20E-02	3.03E-01	4.02E-02	9.98E-01	6.47E-01	4.08E-02	6.41E-01	6.99E-03	1.04E-02	6.38E-02	4.22E-02	4.93E-01	6.29E-01	4.39E-02	1.96E-01	5.01E-02	1
**I13391**	8.94E-01	1.60E-01	6.44E-01	1.78E-01		4.31E-02	8.69E-01	1.69E-01	9.19E-02	4.48E-02	4.10E-02	8.87E-02	2.50E-02	1.69E-02	8.64E-01	1.01E-01	2.84E-01	2.55E-02	2.57E-01	7.06E-01	1.63E-03	1
**I13390**	2.92E-01	8.38E-02	1.83E-01	1.20E-02	4.31E-02		1.12E-01	3.35E-01	2.28E-01	4.09E-02	3.46E-01	3.14E-01	1.32E-01	3.69E-02	3.08E-01	7.36E-01	6.40E-02	1.44E-01	5.29E-01	8.91E-01	5.53E-03	1
**I13389**	9.83E-01	5.94E-01	9.97E-01	3.03E-01	8.69E-01	1.12E-01		5.65E-02	6.65E-01	5.26E-01	6.13E-01	1.81E-01	1.70E-01	2.84E-02	6.77E-01	1.11E-01	2.61E-01	3.62E-01	2.10E-01	7.01E-01	4.91E-02	0
**I13387**	4.63E-01	5.18E-02	1.80E-02	4.02E-02	1.69E-01	3.35E-01	5.65E-02		1.20E-01	2.18E-02	5.79E-02	1.15E-01	1.37E-01	2.97E-02	1.24E-01	2.91E-01	3.11E-01	4.68E-02	1.89E-01	5.99E-01	1.15E-03	1
**I13386**	9.46E-01	6.39E-01	6.85E-01	9.98E-01	9.19E-02	2.28E-01	6.65E-01	1.20E-01		7.20E-01	1.84E-01	9.19E-01	7.21E-02	2.57E-02	8.45E-02	2.35E-01	5.98E-01	9.03E-01	1.84E-01	2.82E-01	5.62E-02	0
**I13385**	6.58E-01	7.67E-01	4.73E-01	6.47E-01	4.48E-02	4.09E-02	5.26E-01	2.18E-02	7.20E-01		9.04E-01	5.94E-01	1.01E-01	7.95E-02	2.69E-01	2.84E-02	4.15E-01	9.80E-01	1.81E-02	4.84E-01	1.07E-01	0
**I13384**	4.60E-01	6.85E-01	4.44E-01	4.08E-02	4.10E-02	3.46E-01	6.13E-01	5.79E-02	1.84E-01	9.04E-01		4.64E-01	2.87E-01	1.07E-01	7.10E-01	2.62E-01	2.00E-01	4.34E-01	1.03E-01	9.10E-01	2.07E-01	0
**I13383**	7.86E-01	7.39E-01	4.13E-01	6.41E-01	8.87E-02	3.14E-01	1.81E-01	1.15E-01	9.19E-01	5.94E-01	4.64E-01		8.93E-02	3.85E-01	3.42E-01	4.26E-01	9.38E-01	7.88E-01	5.26E-01	4.48E-01	4.39E-02	0
**I13382**	3.54E-01	9.36E-02	1.19E-02	6.99E-03	2.50E-02	1.32E-01	1.70E-01	1.37E-01	7.21E-02	1.01E-01	2.87E-01	8.93E-02		1.48E-03	4.93E-02	1.55E-01	1.42E-02	5.08E-02	1.02E-01	9.58E-01	3.19E-02	2
**I13381**	1.02E-01	2.84E-01	4.63E-02	1.04E-02	1.69E-02	3.69E-02	2.84E-02	2.97E-02	2.57E-02	7.95E-02	1.07E-01	3.85E-01	1.48E-03		5.20E-01	4.32E-02	2.47E-01	5.49E-01	6.11E-02	3.29E-01	6.78E-02	1
**I13380**	8.34E-01	7.44E-01	6.29E-01	6.38E-02	8.64E-01	3.08E-01	6.77E-01	1.24E-01	8.45E-02	2.69E-01	7.10E-01	3.42E-01	4.93E-02	5.20E-01		4.09E-01	4.52E-01	1.82E-01	5.02E-01	9.43E-01	4.50E-02	0
**I13379**	8.20E-01	1.91E-01	2.17E-01	4.22E-02	1.01E-01	7.36E-01	1.11E-01	2.91E-01	2.35E-01	2.84E-02	2.62E-01	4.26E-01	1.55E-01	4.32E-02	4.09E-01		5.21E-01	7.63E-02	5.48E-01	7.31E-01	3.22E-02	0
**I13378**	8.37E-01	6.07E-01	5.83E-01	4.93E-01	2.84E-01	6.40E-02	2.61E-01	3.11E-01	5.98E-01	4.15E-01	2.00E-01	9.38E-01	1.42E-02	2.47E-01	4.52E-01	5.21E-01		7.43E-01	2.72E-02	3.86E-01	9.64E-03	1
**I13377**	5.32E-01	9.76E-01	2.85E-01	6.29E-01	2.55E-02	1.44E-01	3.62E-01	4.68E-02	9.03E-01	9.80E-01	4.34E-01	7.88E-01	5.08E-02	5.49E-01	1.82E-01	7.63E-02	7.43E-01		9.35E-02	3.23E-01	1.97E-01	0
**I13376**	3.06E-01	1.22E-01	6.74E-02	4.39E-02	2.57E-01	5.29E-01	2.10E-01	1.89E-01	1.84E-01	1.81E-02	1.03E-01	5.26E-01	1.02E-01	6.11E-02	5.02E-01	5.48E-01	2.72E-02	9.35E-02		8.58E-01	1.66E-02	0
**I13128**	7.46E-01	6.37E-01	5.46E-01	1.96E-01	7.06E-01	8.91E-01	7.01E-01	5.99E-01	2.82E-01	4.84E-01	9.10E-01	4.48E-01	9.58E-01	3.29E-01	9.43E-01	7.31E-01	3.86E-01	3.23E-01	8.58E-01		2.04E-01	0
**I13125**	2.76E-02	3.12E-01	3.31E-02	5.01E-02	1.63E-03	5.53E-03	4.91E-02	1.15E-03	5.62E-02	1.07E-01	2.07E-01	4.39E-02	3.19E-02	6.78E-02	4.50E-02	3.22E-02	9.64E-03	1.97E-01	1.66E-02	2.04E-01		4

Sicily_Himera_480BCE_1

Pairwise *qpWave* tests (P16+Greece_LBA) confirm the extreme genetic variation in the soldiers of the 480 BCE battle (Table S5), already shown in PCA and ADMIXTURE (Figs. 2, S2 and S11) and as expected genetic homogeneity is rejected by *qpfmv* (p<<0.01). One pair of individuals (I10945/W0494 and I10948/W2587) that falls within the "Aegean-like" cluster appears inconsistent with forming a clade in *qpWave* with a p-value of 6.13E-04. However, these individuals are each cladal with the combined groups of the other individuals (p=0.0253 for I10945/ W0494 and p=0.936 for I10948/W2587) and so we include them in the genetic analysis grouping of "Aegean-like" individuals which we refer to as Siciliy_Himera_480BCE_1. However, the presence of subtle inhomogeneity within this group is suggested by *qpfmv*, with a p-value of 1.14E-3.

Table S5. P-values for the *qpWave* models for pairwise combinations of individuals associated with the 480 BCE battle of Himera. Rejected models with p<0.01 marked in red.

	I10945	I10948	I10952	I7217	I7218	I7219	I7221	number of rejected tests
**I10945**		6.13E-04	7.98E-02	7.00E-02	9.79E-02	2.95E-02	1.37E-01	1
**I10948**	6.13E-04		9.94E-02	3.82E-01	8.52E-01	2.60E-01	3.21E-02	1
**I10952**	7.98E-02	9.94E-02		3.69E-01	1.66E-01	3.50E-01	6.29E-01	0
**I7217**	7.00E-02	3.82E-01	3.69E-01		6.55E-01	5.27E-02	4.26E-01	0
**I7218**	9.79E-02	8.52E-01	1.66E-01	6.55E-01		6.67E-01	3.62E-01	0
**I7219**	2.95E-02	2.60E-01	3.50E-01	5.27E-02	6.67E-01		6.02E-01	0
**I7221**	1.37E-01	3.21E-02	6.29E-01	4.26E-01	3.62E-01	6.02E-01		0

We define the following groups based on their cladality in *qpWave*, as well as PCA and ADMIXTURE results:

Sicily_Himera_480BCE_2

This group comprises two individuals archaeologically identified as soldiers of the 480 BCE battle that, compared to Himera Group 1, cluster on PCA shifted towards Central Europeans populations (Figs. 2A and S11), and have a higher proportion of the component maximized in WHG in ADMIXTURE (Fig. 2B). This pair of individuals (I10946/W1771 and I10950/W0814) forms a clade in the *qpWave* model with a p-value of 0.869 to the exclusion of any other pairwise models formed with other 480 BCE soldiers (Fig. S13).

Sicily_Himera_480BCE_3

This group comprises two individuals archaeologically identified as soldiers of the 480 BCE battle that cluster on PCA closest to ancient Eastern Baltic populations (Figs. 2A and S11), and have like these the highest proportion of the component maximized in WHG in ADMIXTURE compared to other contemporaneous groups (Fig. 2B). This pair of individuals (I10943/W0396 and I10949/W0403) forms a clade in the *qpWave* model with a p-value of 0.256, to the exclusion of any other pairwise models formed with other 480 BCE soldiers.

Sicily_Himera_480BCE_4

This group comprises two individuals archaeologically identified as soldiers of the 480 BCE battle that cluster on PCA closest to ancient nomadic populations of the Western Steppe (Figs. 2A and S11), and also carry the component maximized in modern-day Han in ADMIXTURE that is found in varying proportions in most ancient Steppe nomads (Fig. 2B). This pair of individuals (I10944/W0461 and I10947/W1774) forms a clade in the *qpWave* model with a p-value of 0.572, to the exclusion of any other pairwise models formed with other 480 BCE soldiers.

Sicily_Himera_480BCE_5

A single individual archaeologically identified as a soldier of the 480 BCE battle clusters on PCA closest to ancient and modern populations from the Caucasus and Iran (Figs. 2A and S11), and also carries the highest proportion of the genetic component maximized in CHG in ADMIXTURE compared to the other ancient Sicilians (Fig. 2B). This individual (I10951/W0653) is not cladal with any of the other 480 BCE soldiers according to the *qpWave* models and thus is treated as his own group.

Sicily_Himera_409BCE

This group comprises five individuals archaeologically identified as soldiers of the 409 BCE battle that cluster on together with LBA Aegeans and the soldiers of Himera Group 1 (Figs. 2A and S11), and are similar in their proportions of genetic components determined by ADMIXTURE (Fig. 2B). All pairs of individuals appear cladal in our *qpWave* models (p>0.05; Table S6) and homogeneity is not rejected by *qpfmv* (p=0.120) and we group them together for further analyses.

Table S6. P-values for the *qpWave* models for pairwise combinations of individuals associated with the 409 BCE battle of Himera.

	**I17866**	**I17884**	**I7223**	**I7224**	**I7225**	**number of rejected tests**
**I17866**		3.48E-01	1.06E-01	2.69E-01	5.89E-02	0
**I17884**	3.48E-01		9.48E-02	3.15E-01	5.46E-02	0
**I7223**	1.06E-01	9.48E-02		7.95E-01	3.71E-01	0
**I7224**	2.69E-01	3.15E-01	7.95E-01		5.92E-02	0
**I7225**	5.89E-02	5.46E-02	3.71E-01	5.92E-02		0

Sicily_Himera_Civilians

Eleven individuals are archaeologically attributed to the civilian population of the colony of Himera who died not in association with either of the two battles. In the PCA and ADMIXTURE the individuals appear similar to Sicily_IA, Aegean and Himera_480BCE_1 and the soldiers of the 409 BCE battle (Figs. 2A, S11 and S12), as well as to Punic individuals. The similarity to Punic individuals is also supported by the presence of a small proportion of the component maximized in Ju|'hoan in some individuals. This, as well as the slight genetic heterogeneity they show, might alternatively be an artefact of the low coverage of the samples. Only three of these individuals are covered on more than 50,000 SNPs – I20163/W1838, I20166/W3182 and I20168/W3702, covered at 109,255, 136,921 and 147,935 SNPs, respectively. Accordingly, our *qpWave* tests are not able to reject most models involving these low coverage samples (Table S7): cladality between the group of low coverage samples and I20163/W1838 (p=0.649), and I20168/W3702 (p=0.252), respectively, are not rejected, while it is for I20166/W3182 (p=3.13E-3).

I20163/W1838 and I20166/W3182 do not form a clade (p=1.75E-04), I20163/W1838 with I20168/W3702 only with a marginal p-value of 1.11E-2, which agrees with their position on the PCA plot with I20166/W3182 and I20168/W3702 clustering more closely to BA Aegeans and Sicily_Himera_480BCE_1, and I20163/W1838 clustering more closely to Sicily_IA. I20166/W3182 and I20168/W3702 are cladal (p=0.205). Using *qpfmv*, homogeneity of the remaining individuals, including low coverage samples, is rejected after removing I20163/W1838 (p<<0.01), I20166/W3182 (p=5.4E-5), and I20168/W3702 (p<<0.01). In subsequent analyses we only use the three higher coverage individuals and do not group them together.

Table S7. P-values for the *qpWave* models for pairwise combinations of individuals associated with the general populace of Himera. Rejected models with p<0.01 marked in red.

	I20163	I20166	I20168	I17878	I17879	I17881	I17887	I20160	I20161	I20162	I20167	number of rejected tests
**I20163**		1.75E-04	1.11E-02	4.92E-01	4.61E-01	3.65E-01	6.43E-01	4.30E-02	5.72E-01	4.27E-02	1.66E-01	1
**I20166**	1.75E-04		2.05E-01	7.90E-03	1.32E-01	2.37E-01	4.85E-01	3.49E-01	6.38E-02	1.83E-03	5.88E-02	2
**I20168**	1.11E-02	2.05E-01		3.55E-02	9.10E-02	7.86E-01	7.71E-01	2.40E-01	6.01E-01	1.45E-01	1.72E-01	0
**I17878**	4.92E-01	7.90E-03	3.55E-02		4.45E-01	4.35E-01	2.83E-01	2.47E-01	2.44E-01	7.50E-02	1.80E-02	1
**I17879**	4.61E-01	1.32E-01	9.10E-02	4.45E-01		9.57E-01	4.94E-01	4.84E-02	1.14E-01	1.81E-01	3.60E-02	0
**I17881**	3.65E-01	2.37E-01	7.86E-01	4.35E-01	9.57E-01		5.87E-01	5.24E-02	2.35E-01	7.05E-01	8.86E-02	0
**I17887**	6.43E-01	4.85E-01	7.71E-01	2.83E-01	4.94E-01	5.87E-01		7.69E-01	6.31E-01	6.94E-01	8.10E-01	0
**I20160**	4.30E-02	3.49E-01	2.40E-01	2.47E-01	4.84E-02	5.24E-02	7.69E-01		7.91E-01	1.22E-01	3.90E-01	0
**I20161**	5.72E-01	6.38E-02	6.01E-01	2.44E-01	1.14E-01	2.35E-01	6.31E-01	7.91E-01		6.74E-01	5.56E-01	0
**I20162**	4.27E-02	1.83E-03	1.45E-01	7.50E-02	1.81E-01	7.05E-01	6.94E-01	1.22E-01	6.74E-01		4.78E-02	1
**I20167**	1.66E-01	5.88E-02	1.72E-01	1.80E-02	3.60E-02	8.86E-02	8.10E-01	3.90E-01	5.56E-01	4.78E-02		0

After we established genetic groups and outliers, we investigated their genetic affinities and ancestry with outgroup f_3-statistics of the form f_3(Sicilian group, *X*; Mbuti.DG) to measure shared genetic drift between Sicilian groups and ancient populations *X* relative to the outgroup Mbuti.DG. We show the results in Fig. S16.

Ancestry modeling with qpAdm

We used *qpAdm (42)* with the parameter *allsnps: YES* with precomputed *f*-statistics from *qpfstats* to estimate proportions of ancestry in each individual (Dataset S5) as well as in the established analysis groupings (Table S8) using as source proxies distantly related populations. We modeled the individuals/groups as combinations of 1 to 4 distal sources from the set Turkey_N_Barcin, WHG, Iran_GanjDareh_N, Russia_Samara_EBA_Yamnaya with the outgroups Mbuti.DG, Russia_Ust_Ishim_HG.DG, CHG, EHG, Spain_ElMiron, Czech_Vestonice16, Russia_MA1_HG.SG, Israel_Natufian, Jordan_PPNB, Morocco_EN.SG and Russia_Shamanka_Eneolithic.SG (P11). We added the unused sources in each model to the outgroups, as this allows us to better distinguish possible models and decreases the standard errors. Only when this approach led to no feasible model did we apply a less stringent approach and report the feasible model using only the base outgroup set P11.

We confirm the appearance of an Iran-related ancestry component in the MBA (43) that is not present to the same extent or at all in the previous EBA and makes up 9-13% of the group's ancestry (p=5.13E-02; Table S8). The model of this group involving the least sources does not require input from Russia_Samara_EBA_Yamnaya, although EBA requires 7-10% of this ancestry. The later Sicily_LBA can be modeled (p=8.36E-02) using only the three sources Turkey_N_Barcin (83.9 ± 1.6%), WHG (5.4 ± 1.6%) and Russia_Samara_EBA_Yamnaya (10.6 ± 2.1%).

Table S8. Admixture proportions estimated by *qpAdm* for the most parsimonious models with distal sources. The sources used are Turkey_N_Barcin (1), WHG (2), Iran_GanjDareh_N/CHG (marked with †) (3), Russia_Samara_EBA_Yamnaya (4), and Russia_Shamanka_Eneolithic.SG (5).

		Ancestry coefficients					Ancestry coefficient standard errors				
Group	p-value	1	2	3	4	5	1	2	3	4	5
Sicily_EBA	8.69E-02	0.787	0.118	0.095			0.018	0.011	0.017		
Sicily_EBA	4.21E-01	0.822	0.072		0.107		0.014	0.014		0.018	
Sicily_MBA	1.04-01	0.793	0.07	0.137			0.024	0.016	0.023		
Sicily_LBA	1.44E-02	0.797	0.113	0.09			0.021	0.014	0.020		
Sicily_LBA	3.7E-01	0.823	0.047		0.13		0.016	0.016		0.020	
Sicily_IA	1.79E-01	0.764	0.064	0.063	0.109		0.012	0.010	0.015	0.016	
Sicily_Himera_480BCE_1	5.71E-02	0.764		0.058	0.179		0.013		0.017	0.015	
Sicily_Himera_480BCE_2	7.39E-01	0.621	0.084		0.296		0.02	0.020		0.025	
Sicily_Himera_480BCE_3	1.77E-02	0.319	0.182		0.499		0.019	0.020		0.025	
Sicily_Himera_480BCE_4	8.54E-01	0.212		0.062†	0.624	0.102	0.024		0.036	0.032	0.024
Sicily_Himera_480BCE_5	1.73E-02	0.334	0.013	0.452†	0.201		0.037	0.035	0.058	0.064	
Sicily_Himera_409BCE	1.30E-02	0.766			0.234		0.015			0.015	
Sicily_Himera_Civilians_I20163	3.09E-02	0.936	0.064				0.036	0.036			
Sicily_Himera_Civilians_I20166	5.83E-02	0.667		0.215	0.119		0.048		0.058	0.049	
Sicily_Himera_Civilians_I20168	6.10E-02	0.749		0.251			0.046		0.046		
Sicily_Himera_Civilians_I20168	5.43E-02	0.786			0.214		0.043			0.043	
Sicily_Himera_EastNecropolis	3.29E-02	0.899	0.101				0.02	0.020			

As more proximal ancestry sources in the *qpAdm* analysis, we used diverse Eneolithic to IA populations that appear to have affinity to the analysis groups informed by the results of PCA, ADMIXTURE and outgroup f_3-statistics, using the outgroup set P11 and adding additional outgroups Turkey_N_Barcin, WHG, Iran_GanjDareh_N, Russia_Samara_EBA_Yamnaya and Sicily_EBA. We examine all possible 1-, 2- and 3-way models and consider models valid if their fit was p>0.01and report the most parsimonious model that uses the least sources. In cases where several models from the most parsimonious rank are not rejected, we apply a "model competition" approach in which we add all unused sources to the outgroup set P16.

Sicily_IA

As individuals (Dataset S5), Sicily_IA could be modeled with the predominant ancestry related to Turkey_N_Barcin (~68-85%, with two low coverage individuals having a feasible model of 100% Turkey_N_Barcin), with additional ancestry coming from Russia_Samara_EBA_Yamnaya in 22 of 25 individuals (~4-23%), as well as WHG in 19 individuals (~2-11%), and 12 individuals alternatively modeled with Iran_GanjDareh_N instead of Russia_Samara_EBA_Yamnaya (~10-21%). The complete group of Sicily_IA can be modeled as a mixture of four distal sources (p=1.79E-01; Table S13) Turkey_N_Barcin (76.4 ± 1.2%), WHG (6.4 ± 0.9%), Iran_GanjDareh_N (6.3 ± 1.5%) and Russia_Samara_EBA_Yamnaya 10.9 ± 1.6%). Thus, for Sicily we have evidence of Iran-related gene flow not mediated by the Bronze Age Steppe expansion for which we use Yamnaya as proxy. This additional Iran-related ancestry was reported to be already present in Sicily in MBA and LBA individuals (43). Our models confirm the Iranian-related ancestry in Sicily_MBA (13.7 ± 2.3%), and cannot reject one of the alternative models that uses it as a source (9.0 ± 2%; p=1.44E-02).

As proxies for more proximal sources of ancestry, we tested 1-, 2-, and 3-way models of the sources Greece_Crete_BA, Greece_LBA, Armenia_LBA.SG, Sicily_LBA, Sicily_LBA_I10371, Sicily_MBA, Sicily_Himera_480BCE_1, Balkan_IA, Italy_IA_Republic.SG, Israel_MLBA_Canaanite, Spain_BA, Spain_IA and Italy_Sardinia_IA_Punic_1 (Table S9). All working models include either Sicily_MBA

or Sicily_LBA_I10371 as a local source contributing to the at least 60% of the ancestry and either Italy_IA_Republic.SG, Spain_BA, Spain_IA or Armenia_LBA.SG as a non-Sicilian source. Several models involving the most temporally proximal source Sicily_LBA are consistent with 0% ancestry deriving from an additional source.

Table S9. Admixture proportions estimated by *qpAdm* for the most parsimonious valid models for Sicily_IA with proximal sources.

Admixture Sources		p-value	Ancestry coefficients		Standard errors		Model comp.?
1	2		Anc_1	Anc_2	1	2	
Sicily_LBA	Balkan_IA	2.11E-02	0.875	0.125	0.066	0.066	no
Sicily_Himera_480BCE_1	Sicily_LBA	1.45E-02	0.154	0.846	0.089	0.089	no
Sicily_LBA	Italy_IA_Republic.SG	1.62E-02	0.912	0.088	0.047	0.047	no
Sicily_LBA	Armenia_LBA.SG	1.60E-02	0.962	0.038	0.02	0.02	no
Sicily_LBA_I10371	Italy_IA_Republic.SG	4.18E-01	0.542	0.458	0.05	0.05	no
Sicily_LBA_I10371	Spain_IA	3.63E-01	0.595	0.405	0.05	0.05	no
Sicily_LBA_I10371	Spain_BA	1.83E-01	0.572	0.428	0.051	0.051	no
Sicily_MBA	Balkan_IA	3.84E-02	0.786	0.214	0.072	0.072	no
Sicily_MBA	Italy_IA_Republic.SG	2.85E-02	0.859	0.141	0.044	0.044	no
Sicily_LBA_I10371	Balkan_IA	1.29E-02	0.571	0.429	0.105	0.105	yes
Sicily_LBA_I10371	Italy_IA_Republic.SG	2.34E-01	0.604	0.396	0.056	0.056	yes
Sicily_LBA	Sicily_Himera_480BCE_1	1.29E-02	0.917	0.083	0.088	0.088	yes
Sicily_LBA_I10371	Spain_IA	4.29E-01	0.602	0.398	0.047	0.047	yes
Sicily_LBA_I10371	Spain_BA	1.79E-01	0.588	0.412	0.051	0.051	yes
Sicily_MBA	Balkan_IA	1.71E-02	0.818	0.182	0.077	0.077	yes
Sicily_MBA	Italy_IA_Republic.SG	5.22E-02	0.875	0.125	0.041	0.041	yes
Sicily_MBA	Spain_IA	1.10E-02	0.886	0.114	0.038	0.038	yes
Sicily_LBA	Sicily_MBA	3.69E-02	0.782	0.218	27.498	27.498	yes
Sicily_LBA	Balkan_IA	3.21E-02	0.905	0.095	0.064	0.064	yes
Sicily_LBA	Italy_IA_Republic.SG	5.33E-02	0.91	0.09	0.04	0.04	yes
Sicily_LBA	Armenia_LBA.SG	1.89E-02	0.966	0.034	0.019	0.019	yes
Sicily_LBA	Spain_IA	1.55E-02	0.987	0.013	0.04	0.04	yes

Sicily_Himera_480BCE_1

The individuals of this group could all be modeled as a 2-way mixture of ~76-83% Turkey_N_Barcin and ~17-24% Russia_Samara_EBA_Yamnaya, requiring no additional WHG ancestry (Dataset S5). As a group, the feasible model (p=1.21E-02; Table S13) is a 3-way mixture of Turkey_N_Barcin (76.4± 1.3%), Iran_GanjDareh_N (5.8 ± 1.5%) and Russia_Samara_EBA_Yamnaya (17.9 ± 1.3%).

We tested 1-, 2-, and 3-way models of the proximal sources Greece_Crete_BA, Greece_LBA, Sicily_LBA, Sicily_LBA_I10371, Sicily_MBA, Sicily_IA, Sicily_Himera_Civilians_I20163, Sicily_Himera_Civilians_I20166, Sicily_Himera_Civilians_I20168, Balkan_IA, Italy_IA_Republic.SG, Israel_MLBA_Canaanite, Spain_IA and Italy_Sardinia_IA_Punic_1 (Table S10). After model competition, four 2-way models had a p-value>0.05, involving at least one source that accounts for an Iranian related component (Greece_Crete_BA, Greece_LBA, Armenia_LBA.SG, Sicily_LBA_I10371, Sicily_Himera_Civilians_I20166, Sicily_Himera_Civilians_I20168). Some working 2-way models involve one with a lower proportion of Iranian-related ancestry: Italy_IA_Republic.SG, Balkan_IA, Sicily_MBA, Sicily_IA. In the model-competition approach, two 2-way models are not rejected that involve sources comprised of more than a single individual (meaning there is more statistical power to reject these models): 1) 46.5 ± 4.7% from Greece_Crete_BA and 53.5 ± 4.7% from Balkans_IA (p=3.83E-01), and 2) 79.2 ± 11.5% from Greece_LBA and 20.8 ± 11.5% from Balkans_IA (p=1.20E-02). According to these results, the soldiers in the 480 BCE battle carry a large part of ancestry that can be traced back to the BA inhabitants of the Aegean ("Mycenaeans" and "Minoans") which could represent both the descendants of Greek settlers in Sicily or other colonies or possibly direct migrants from the Aegean.

Table S10. Admixture proportions estimated by *qpAdm* for the most parsimonious valid models for Sicily_Himera_480BCE_1 with proximal sources.

Admixture Sources		p-value	Ancestry coefficients		Standard errors		Model comp.?
1	2		Anc_1	Anc_2	SE_1	SE_2	
Sicily_IA	Sicily_Himera_Civilians_I20166	1.28E-02	0.482	0.518	0.09	0.09	no
Sicily_IA	Sicily_Himera_Civilians_I20168	1.61E-01	0.488	0.512	0.082	0.082	no
Greece_Crete_BA	Balkan_IA	4.87E-01	0.474	0.526	0.043	0.043	no
Greece_Crete_BA	Sicily_Himera_Civilians_I20168	4.90E-02	0.367	0.633	0.1	0.1	no
Greece_LBA	Sicily_LBA_I10371	1.45E-02	0.837	0.163	0.366	0.366	no
Sicily_LBA	Sicily_Himera_Civilians_I20168	7.20E-02	0.401	0.599	0.092	0.092	no
Sicily_LBA_I10371	Balkan_IA	3.73E-02	0.381	0.619	0.092	0.092	no
Sicily_LBA_I10371	Italy_IA_Republic.SG	1.10E-02	0.567	0.433	0.058	0.058	no
Sicily_LBA_I10371	Sicily_Himera_Civilians_I20168	4.11E-01	0.337	0.663	0.098	0.098	no
Sicily_LBA_I10371	Armenia_LBA.SG	2.71E-01	0.675	0.325	0.039	0.039	no
Sicily_MBA	Sicily_Himera_Civilians_I20166	2.78E-02	0.51	0.49	0.896	0.896	no
Sicily_MBA	Sicily_Himera_Civilians_I20168	1.10E-01	0.437	0.563	0.314	0.314	no
Greece_Crete_BA	Sicily_Himera_Civilians_I20168	2.22E-02	0.302	0.698	0.127	0.127	yes
**Greece_Crete_BA**	**Balkan_IA**	**3.83E-01**	**0.465**	**0.535**	**0.047**	**0.047**	**yes**
Greece_LBA	Sicily_LBA_I10371	2.12E-02	0.869	0.131	0.224	0.224	yes
**Greece_LBA**	**Sicily_MBA**	**1.20E-02**	**0.792**	**0.208**	**0.115**	**0.115**	**yes**
Sicily_LBA	Sicily_Himera_Civilians_I20166	2.23E-02	0.394	0.606	0.086	0.086	yes
Sicily_LBA	Sicily_Himera_Civilians_I20168	1.04E-01	0.343	0.657	0.102	0.102	yes
Sicily_LBA_I10371	Sicily_Himera_Civilians_I20166	5.08E-02	0.328	0.672	0.78	0.78	yes
Sicily_LBA_I10371	Sicily_Himera_Civilians_I20168	1.83E-01	0.302	0.698	0.187	0.187	yes
Sicily_LBA_I10371	Balkan_IA	4.44E-02	0.361	0.639	0.128	0.128	yes
Sicily_LBA_I10371	Italy_IA_Republic.SG	6.78E-02	0.58	0.42	0.059	0.059	yes
Sicily_LBA_I10371	Armenia_LBA.SG	1.86E-01	0.692	0.308	0.04	0.04	yes
Sicily_MBA	Sicily_Himera_Civilians_I20166	1.01E-01	0.454	0.546	0.093	0.093	yes

Sicily_Himera_480BCE_2

These two individuals could be modeled as 3-way models of ~60-62% Turkey_N_Barcin, ~7-10% WHG and ~29-30% Russia_Samara_EBA_Yamnaya (Dataset S5). As a group, the ancestry coefficients were estimated as 62.1 ± 2% Turkey_N_Barcin, 8.4 ± 2% WHG and 29.6 ± 2.5% Russia_Samara_EBA_Yamnaya (p=7.39E-01; Table S13).

We tested 1-, 2-, and 3-way models of the proximal sources Greece_Crete_BA, Greece_LBA, Sicily_LBA, Sicily_MBA, Balkan_IA, Italy_IA_Republic.SG, Spain_IA, Italy_IA_Republic_o.SG, Hungary_IA_Scythian.SG, Czech_HallstattBylany.SG, Sicily_IA, France_GrandEst_IA1.SG, France_GrandEst_IA2.SG and France_HautsDeFrance_IA2.SG (Table S11). Many 2-way models are feasible, even using the model competition approach, and in all of them Sicily_Himera_480BCE_2 derives at least half of its ancestry from a western European, central European or Balkan IA group.

Table S11. Admixture proportions estimated by *qpAdm* for the most parsimonious valid models for Sicily_Himera_480BCE_2 with proximal sources.

Admixture Sources		p-value	Ancestry coefficients		Standard errors		Model
1	2		Anc_1	Anc_2	SE_1	SE_2	comp.?
Greece_Crete_BA	France_GrandEst_IA1.SG	9.06E-01	0.266	0.734	0.037	0.037	no
Greece_Crete_BA	France_GrandEst_IA2.SG	6.78E-01	0.32	0.68	0.034	0.034	no
Greece_Crete_BA	France_HautsDeFrance_IA2.SG	9.47E-01	0.367	0.633	0.036	0.036	no
Greece_LBA	Spain_IA	1.96E-02	0.429	0.571	0.058	0.058	no
Greece_LBA	Czech_HallstattBylany.SG	2.29E-02	0.516	0.484	0.048	0.048	no
Greece_LBA	France_GrandEst_IA1.SG	7.96E-01	0.376	0.624	0.053	0.053	no
Greece_LBA	France_GrandEst_IA2.SG	3.74E-01	0.443	0.557	0.048	0.048	no
Greece_LBA	France_HautsDeFrance_IA2.SG	6.48E-01	0.489	0.511	0.048	0.048	no
Sicily_LBA	France_GrandEst_IA1.SG	4.71E-01	0.341	0.659	0.051	0.051	no
Sicily_LBA	France_GrandEst_IA2.SG	6.52E-01	0.411	0.589	0.044	0.044	no
Sicily_LBA	France_HautsDeFrance_IA2.SG	7.28E-01	0.455	0.545	0.046	0.046	no
Sicily_MBA	France_GrandEst_IA1.SG	3.28E-01	0.302	0.698	0.047	0.047	no
Sicily_MBA	France_GrandEst_IA2.SG	3.50E-01	0.37	0.63	0.041	0.041	no
Sicily_MBA	France_HautsDeFrance_IA2.SG	8.41E-01	0.422	0.578	0.042	0.042	no
Balkan_IA	Italy_IA_Republic.SG	6.25E-02	0.485	0.515	0.144	0.144	no
Balkan_IA	Spain_IA	4.34E-01	0.606	0.394	0.079	0.079	no
Balkan_IA	Hungary_Scythian.SG	2.76E-01	0.684	0.316	0.062	0.062	no
Balkan_IA	Czech_HallstattBylany.SG	3.75E-01	0.683	0.317	0.064	0.064	no
Balkan_IA	France_GrandEst_IA1.SG	8.50E-01	0.523	0.477	0.081	0.081	no
Balkan_IA	France_GrandEst_IA2.SG	3.34E-01	0.622	0.378	0.075	0.075	no
Balkan_IA	France_HautsDeFrance_IA2.SG	5.54E-01	0.655	0.345	0.07	0.07	no
Spain_IA	Italy_IA_Republic_o.SG	3.83E-02	0.45	0.55	0.094	0.094	no
Sicily_IA	France_GrandEst_IA1.SG	6.28E-01	0.365	0.635	0.05	0.05	no
Sicily_IA	France_GrandEst_IA2.SG	6.11E-01	0.431	0.569	0.044	0.044	no
Sicily_IA	France_HautsDeFrance_IA2.SG	9.42E-01	0.48	0.52	0.043	0.043	no
Greece_Crete_BA	Czech_HallstattBylany.SG	1.39E-02	0.37	0.63	0.035	0.035	no
Greece_LBA	Spain_IA	6.15E-02	0.431	0.569	0.06	0.06	yes
Greece_LBA	France_GrandEst_IA1.SG	4.03E-02	0.425	0.575	0.056	0.056	yes
Greece_LBA	France_GrandEst_IA2.SG	1.23E-01	0.458	0.542	0.051	0.051	yes
Greece_LBA	France_HautsDeFrance_IA2.SG	4.49E-01	0.498	0.502	0.05	0.05	yes
Sicily_LBA	France_GrandEst_IA1.SG	2.11E-02	0.387	0.613	0.05	0.05	yes
Sicily_LBA	France_GrandEst_IA2.SG	4.41E-01	0.417	0.583	0.043	0.043	yes
Sicily_LBA	France_HautsDeFrance_IA2.SG	4.93E-01	0.44	0.56	0.046	0.046	yes
Sicily_MBA	France_GrandEst_IA1.SG	3.66E-02	0.362	0.638	0.046	0.046	yes
Sicily_MBA	France_GrandEst_IA2.SG	4.24E-01	0.386	0.614	0.041	0.041	yes
Sicily_MBA	France_HautsDeFrance_IA2.SG	7.11E-01	0.423	0.577	0.042	0.042	yes
Balkan_IA	Spain_IA	4.35E-01	0.614	0.386	0.092	0.092	yes
Balkan_IA	France_GrandEst_IA1.SG	3.00E-01	0.644	0.356	0.093	0.093	yes
Balkan_IA	France_GrandEst_IA2.SG	4.10E-01	0.654	0.346	0.078	0.078	yes
Balkan_IA	France_HautsDeFrance_IA2.SG	4.81E-01	0.676	0.324	0.08	0.08	yes
Balkan_IA	Czech_HallstattBylany.SG	1.00E-01	0.793	0.207	0.057	0.057	yes
Balkan_IA	Italy_IA_Republic.SG	5.43E-02	0.65	0.35	0.118	0.118	yes
Balkan_IA	Hungary_Scythian.SG	7.66E-02	0.797	0.203	0.059	0.059	yes
Spain_IA	Italy_IA_Republic_o.SG	2.20E-02	0.562	0.438	0.079	0.079	yes
Sicily_IA	France_GrandEst_IA1.SG	1.67E-02	0.399	0.601	0.048	0.048	yes
Sicily_IA	France_GrandEst_IA2.SG	2.70E-01	0.428	0.572	0.043	0.043	yes
Sicily_IA	France_HautsDeFrance_IA2.SG	5.06E-01	0.463	0.537	0.043	0.043	yes
Greece_Crete_BA	France_GrandEst_IA2.SG	1.38E-01	0.325	0.675	0.035	0.035	yes
Greece_Crete_BA	France_HautsDeFrance_IA2.SG	4.17E-01	0.366	0.634	0.036	0.036	yes

Sicily_Himera_480BCE_3

As individuals they could be modeled as 3-way models of ~31-32% Turkey_N_Barcin, ~16-20% WHG and ~48-52% Russia_Samara_EBA_Yamnaya (Dataset S5). As a group (p=3.16E-02; Table S8); with 31.9 ± 1.9% Turkey_N_Barcin, 18.2 ± 2 % WHG and 49.9 ± 2.5 % Russia_Samara_EBA_Yamnaya.

As combinations of more proximal sources of ancestry, we tested 1-, 2-, and 3-way models of Sicily_IA, Sicily_Himera_480BCE_1, Estonia_IA.SG, Latvia_BA, Lithuania_BA, WesternSteppe_IA_Nomadic_NE, WesternSteppe_IA_Nomadic_Steppe and England_IA.SG.

The only model that was not rejected was a 1-way model with Lithuania_BA as the source (p=0.129). This group, comprising 4 Eastern Baltic individuals dated to the 13th to 7th century BCE,

carries a greater proportion of WHG and EHG ancestry than other contemporary European populations, but lacks the Siberian-related ancestry found in this region in later times and roughly contemporaneously further north (44, 45).

Sicily_Himera_480BCE_4

This group yields no feasible models when using only the common 4 distal sources for European populations. Due to the position on the PCA clustering with IA steppe nomads and the additional ancestry component maximized in East Asians (Fig. 2B), we moved Russia_Shamanka_Eneolithic.SG to the sources, accounting for any East Asian-related ancestry also seen in varying proportions in contemporaneous Steppe nomads (46, 47), resulting in models that are not rejected. The individuals can be modeled as 20-28% Turkey_N_Barcin, 59-63% Russia_Samara_EBA_Yamnaya and 12-16% Russia_Shamanka_Eneolithic.SG (Dataset S5). As a group, the best-fitting model (p=6.19E-01; Table S8) is Turkey_N_Barcin (21.2 ± 2.4%), Russia_Samara_EBA_Yamnaya (62.4 ± 3.2%), Russia_Shamanka_Eneolithic.SG (10.2 ± 2.4%) and CHG (6.2 ± 3.6%).

Using more proximal sources of ancestry, we tested 1-, 2-, and 3-way models of, CentralSteppe_IA_Nomadic_EA1, CentralSteppe_IA_Nomadic_EA2, CentralSteppe_IA_Nomadic_o, CentralSteppe_IA_Nomadic_Steppe, WesternSteppe_IA_Nomadic_EA, WesternSteppe_IA_Nomadic_intermediate, WesternSteppe_IA_Nomadic_NE, WesternSteppe_IA_Nomadic_o, WesternSteppe_IA_Nomadic_Steppe, TianShan_IA_Nomadic, TianShan_IA_Nomadic_o1, TianShan_IA_Nomadic_o2, TianShan_IA_Nomadic_o3, SouthernSiberia_IA_Nomadic, WesternSteppe_IA_Nomadic_SE, Hungary_IA_Prescythian.SG and Hungary_IA_Scythian.SG (Table S12). We find a working 1-way model in TianShan_IA_Nomadic_o2 (p=1.53E-01; Table S12), a genetic outlier who appears to carry more Western Eurasian ancestry than the main cluster of contemporaneous nomads sampled from the TianShan region. Since this group is only represented by a single individual, we also report working 2-way models, which include the Mediterranean sources or groups known to have more western Eurasian affinity. We investigate the possible source of western Eurasian admixture further by running the competitive modeling approach. While none of the models return a p-value>0.01, the 2-way models with the highest p-value (p>3.86E-04), derive ~88% of ancestry from CentralSteppe_IA_Nomadic_Steppe (i.e. individuals associated with nomadic IA cultures excavated in the Central region of the Eurasian steppes, who genetically resemble BA Steppe pastoralists with added East Asian/Siberian gene flow), and ~12% from an Aegean source (represented by Greece_LBA or Sicily_Himera_480BCE_1), consistent with two 3-way models with the highest p-values (1.19E-03 and 1.27E-03, respectively) that require, in addition to ~79% CentralSteppe_IA_Nomadic_Steppe and ~13-15% of an Aegean source, around 6-7% ancestry deriving from CentralSteppe_IA_Nomadic_EA1 (Table S12). The rejection of these models suggests that while a western Eurasian, and possibly Mediterranean source is plausible, we have not used the exact contributing source in our models. This admixture between groups from the steppe and western Eurasia could have taken place in the steppe, since IA Steppe nomads such as the Scythians were genetically very homogeneous and also included individuals of mostly Aegean ancestry (47, 48); furthermore, Aegean migrants living in Greek colonies on the shores of the Black Sea are likely to have been in contact with the neighboring nomadic populations.

Table S12. Admixture proportions estimated by *qpAdm* for the most parsimonious valid models for Sicily_Himera_480BCE_4 with proximal sources.

Admixture Sources			p-value	Ancestry coefficients			Standard errors			Model comp.?
1	2	3		Anc_1	Anc_2	Anc_3	SE_1	SE_2	SE_3	
TianShan_IA_Nomadic_o2			1.53E-02	1			0			no
CentralSteppe_IA_Nomadic_EA1	TianShan_IA_Nomadic_o2		1.18E-02	0.03	0.97		0.045	0.045		no
WesternSteppe_IA_Nomadic_Steppe	WesternSteppe_IA_Nomadic_SE		3.59E-02	0.875	0.125		0.032	0.032		no
WesternSteppe_IA_Nomadic_Steppe	Sicily_Himera_480BCE_1		7.34E-02	0.907	0.093		0.022	0.022		no
WesternSteppe_IA_Nomadic_Steppe	Greece_LBA		1.30E-01	0.893	0.107		0.024	0.024		no
TianShan_IA_Nomadic_o1	TianShan_IA_Nomadic_o2		1.44E-02	0.016	0.984		0.015	0.015		no
TianShan_IA_Nomadic_o2	WesternSteppe_IA_Nomadic_SE		2.56E-02	0.914	0.086		0.049	0.049		no
TianShan_IA_Nomadic_o2	Hungary_Scythian.SG		1.05E-02	0.981	0.019		0.059	0.059		no
TianShan_IA_Nomadic_o2	Sicily_Himera_480BCE_1		4.31E-02	0.926	0.074		0.033	0.033		no
TianShan_IA_Nomadic_o2	Greece_LBA		5.66E-02	0.912	0.088		0.036	0.036		no
CentralSteppe_IA_Nomadic_Steppe	Sicily_Himera_480BCE_1		3.86E-04	0.892	0.108		0.021	0.021		yes
CentralSteppe_IA_Nomadic_Steppe	Greece_LBA		5.55E-04	0.872	0.128		0.023	0.023		yes
CentralSteppe_IA_Nomadic_EA1	CentralSteppe_IA_Nomadic_Steppe	Sicily_Himera_480BCE_1	1.19E-03	0.07	0.794	0.136	0.038	0.054	0.024	yes
CentralSteppe_IA_Nomadic_EA1	CentralSteppe_IA_Nomadic_Steppe	Greece_LBA	1.27E-03	0.061	0.788	0.151	0.038	0.054	0.026	yes

Sicily_Himera_480BCE_5

This individual could be modeled as a 4-way model (p=1.73E-2; Dataset S5), as a 4-way model of Turkey_N_Barcin (33.4 ± 3.7%), WHG (1.3 ± 3.5%), Russia_Samara_EBA_Yamnaya (20.1 ± 6.4%) and CHG (45.2 ± 5.8%). This is consistent with no WHG contribution; however, the corresponding 3-way model is rejected (p=4.02E-3).

Using more proximal sources of ancestry, we tested 1-, 2-, and 3-way models of Sicily_IA, Greece_Crete_BA, Greece_LBA, Turkmenistan_IA.SG, Armenia_EBA, Armenia_MBA.SG, Armenia_MBA, Armenia_LBA.SG, Iran_Hasanlu_IA.SG, CentralSteppe_IA_Nomadic_Steppe, TianShan_IA_Nomadic, TianShan_IA_Nomadic_o3, CentralSteppe_IA_Nomadic_o, Turkey_IA_o3.SG, WesternSteppe_IA_Nomadic_intermediate, Tajikistan_Ksirov_Kushan and Iran_IA_HajjiFiruz. A single 1-way model with Armenia_MBA as the source was valid (p=0.293).

Sicily_Himera_409BCE

Three of the individuals worked with 2 sources ~74-80% Turkey_N_Barcin and ~20-26% Russia_Samara_EBA_Yamnaya. I17866/W4380 required 3 sources for alternative models involving Turkey_N_Barcin, Iran_GanjDareh_N and either WHG or Russia_Samara_EBA_Yamnaya. and alternative models involving Turkey_N_Barcin, and either Iran_GanjDareh_N or Russia_Samara_EBA_Yamnaya worked for 17884/W4666 (Dataset S5). The latter two samples also show a slight shift towards Near Eastern populations with respect to the other three, and a slightly higher proportion of the ADMIXTURE component maximized in CHG (Fig. 2B). As a group, they were modeled as 76.6 ± 1.5% Turkey_N_Barcin and 23.4 ± 1.5% Russia_Samara_EBA_Yamnaya (p=1.30E-02; Table S8).

We tested 1-, 2-, and 3-way models of the proximal sources Greece_Crete_BA, Greece_LBA, Armenia_LBA.SG, Sicily_LBA, Sicily_LBA_I10371, Sicily_MBA, Sicily_IA,

Sicily_Himera_480BCE_1, Sicily_Himera_Civilians_I20163, Sicily_Himera_Civilians_I20166, Balkan_IA, Italy_IA_Republic.SG, Israel_MLBA_Canaanite, Spain_IA and Italy_Sardinia_IA_Punic_1 (Table S13).

After model competition, the two valid 1-way suggest cladality between the soldiers of the 409n BCE battle and the preceding Aegean-like cluster from the 480 BCE battle, Sicily_Himera_480BCE_1 (p=2.28E-02) and Greece_LBA (p=3.11E-02).

Table S13. Admixture proportions estimated by *qpAdm* for the most parsimonious valid models for Sicily_Himera_409BCE with proximal sources.

Admixture Sources	p-value	Ancestry coefficients	Standard errors	Model comp. ?
1		Anc_1	SE_1	
Sicily_Himera_480BCE_1	3.29E-02	1	0	no
Greece_LBA	2.43E-02	1	0	no
Sicily_Himera_480BCE_1	2.28E-02	1	0	yes
Greece_LBA	3.11E-02	1	0	yes

<u>Sicily_Himera_Civilians</u>

The three higher coverage individuals in this group were established above to be genetically inhomogeneous.

For I20163/W1838 we obtained the working model 93.6 ± 3.6% Turkey_N_Barcin and 6.4 ± 3.6% WHG (p=3.09E-02; Table S8).

I20166/W3182 requires a 3-way model (p=5.83E-02; Table S12) of Turkey_N_Barcin (66.7± 4.8%), Iran_GanjDareh_N (21.5 ± 5.8%) and Russia_Samara_EBA_Yamnaya (11.9 ± 4.9%).

I20168/W3702 can be modeled alternatively as 74.9 ± 4.6% Turkey_N_Barcin and 25.1 ± 4.6% Iran_GanjDareh_N, or as 78.6 ± 4.3% Turkey_N_Barcin and 21.4 ± 4.3% Russia_Samara_EBA_Yamnaya

For these three individuals, we tested 1-, 2-, and 3-way models of the proximal sources Greece_Crete_BA, Greece_LBA, Armenia_LBA.SG, Sicily_LBA, Sicily_MBA, Sicily_IA, Sicily_Himera_480BCE_1, Sicily_Himera_409BCE, Balkan_IA, Italy_IA_Republic.SG, Israel_MLBA_Canaanite, Spain_IA and Italy_Sardinia_C_o Italy_Sardinia_IA_Punic_1 (Table S14).

I20163/W1838 can be fit by four valid 2-way models, most involving a Sicilian source. The second source is either Spain_IA, Israel_MLBA_Canaanite, Italy_Sardinia_IA_Punic_1 or Italy_Sardinia_C_o. The inclusion of the latter three, a Levantine group, Punic individuals with variable North African and European ancestry, and a Chalcolithic Sardinian with North African ancestry, indicate that this individual is the descendant of local Sicilians who also harbors Levantine or North African ancestry – around 9-15%, if Italy_Sardinia_C_o is representative of unadmixed ancestry of this type. The model with the highest p-value gives 61.6 ± 8.7% Sicily_IA and 38.4 % ± 8.7% Italy_Sardinia_IA_Punic_1 (p=0.295).

Table S14. Admixture proportions estimated by *qpAdm* for the most parsimonious valid models for Sicily_Himera_Civilians_I20163 with proximal sources.

Admixture Sources		p-value	Ancestry coefficients		Standard errors		Model comp.?
1	2		Anc_1	Anc_2	SE_1	SE_2	
Israel_MLBA_Canaanite	Spain_IA	2.48E-02	0.453	0.547	0.061	0.061	no
Sicily_LBA	Armenia_LBA.SG	2.74E-02	0.848	0.152	0.052	0.052	no
Sicily_MBA	Armenia_LBA.SG	2.52E-02	0.847	0.153	0.055	0.055	no
Sicily_LBA	Israel_MLBA_Canaanite	1.56E-02	0.779	0.221	0.086	0.086	no
Sicily_LBA	Italy_IA_Republic.SG	1.18E-02	0.734	0.266	0.123	0.123	no
Sicily_MBA	Italy_IA_Republic.SG	2.48E-02	0.692	0.308	0.118	0.118	no
Sicily_IA	Italy_Sardinia_C_o	3.39E-02	0.87	0.13	0.032	0.032	no
Sicily_LBA	Italy_Sardinia_C_o	1.26E-01	0.877	0.123	0.034	0.034	no
Sicily_MBA	Italy_Sardinia_C_o	3.07E-02	0.9	0.1	0.036	0.036	no
Spain_IA	Italy_Sardinia_IA_Punic_1	1.30E-02	0.438	0.562	0.081	0.081	no
Sicily_IA	Italy_Sardinia_IA_Punic_1	9.85E-02	0.586	0.414	0.094	0.094	no
Sicily_LBA	Italy_Sardinia_IA_Punic_1	2.39E-01	0.616	0.384	0.098	0.098	no
Sicily_MBA	Italy_Sardinia_IA_Punic_1	9.48E-02	0.632	0.368	0.111	0.111	no
Greece_LBA	Spain_IA	1.66E-02	0.673	0.327	0.1	0.1	no
Sicily_LBA	Spain_IA	1.57E-02	0.743	0.257	0.112	0.112	no
Sicily_MBA	Spain_IA	9.35E-02	0.673	0.327	0.096	0.096	no
Sicily_LBA	Italy_Sardinia_C_o	1.06E-02	0.869	0.131	0.033	0.033	yes
Israel_MLBA_Canaanite	Sicily_LBA	2.98E-02	0.276	0.724	0.075	0.075	yes
Sicily_LBA	Italy_Sardinia_IA_Punic_1	1.22E-01	0.552	0.448	0.088	0.088	yes
Sicily_MBA	Spain_IA	9.31E-02	0.649	0.351	0.098	0.098	yes
Sicily_MBA	Armenia_LBA.SG	2.86E-02	0.841	0.159	0.054	0.054	yes
Sicily_MBA	Italy_IA_Republic.SG	5.42E-02	0.675	0.325	0.107	0.107	yes
Sicily_MBA	Italy_Sardinia_C_o	2.57E-02	0.894	0.106	0.035	0.035	yes
Sicily_MBA	Italy_Sardinia_IA_Punic_1	2.06E-01	0.589	0.411	0.104	0.104	yes
Israel_MLBA_Canaanite	Sicily_MBA	2.99E-02	0.225	0.775	0.09	0.09	yes
Sicily_IA	Spain_IA	1.78E-02	0.727	0.273	0.106	0.106	yes
Sicily_IA	Italy_Sardinia_C_o	1.25E-01	0.876	0.124	0.031	0.031	yes
Sicily_IA	Italy_Sardinia_IA_Punic_1	2.95E-01	0.616	0.384	0.087	0.087	yes
Spain_IA	Greece_LBA	1.32E-02	0.317	0.683	0.105	0.105	yes
Israel_MLBA_Canaanite	Sicily_IA	3.55E-02	0.252	0.748	0.078	0.078	yes
Greece_LBA	Italy_Sardinia_IA_Punic_1	1.06E-02	0.629	0.371	0.138	0.138	yes
Israel_MLBA_Canaanite	Spain_IA	2.34E-02	0.44	0.56	0.063	0.063	yes

Both I20166/W3182 and I20168/W3702 can be modeled as cladal with Greece_LBA (p-values=3.55E-02 and 7.80E-02, respectively), likely confirming descendants of Greek settlers among the civilians of Himera.

Sicily_Himera_EastNecropolis

This individual can be modeled distally as 89.9 ± 2% Turkey_N_Barcin and 10.1 ± 2% WHG (p=3.29E-02; Dataset S5).

In proximal 1-way models the individual is cladal with preceding Sicilian groups Sicily_MBA, Sicily_LBA and Sicily_IA (Table S15), consistent with its position on the PCA and ancestry components inferred by ADMIXTURE (Fig. 2).

Table S15. Admixture proportions estimated by *qpAdm* for the most parsimonious valid models for Sicily_Himera_EastNecropolis with proximal sources.

Admixture Sources	p-value	Ancestry coefficients	Standard errors	Model comp.?
1		Anc_1	SE_1	
Sicily_IA	6.69E-02	1	0	no
Sicily_LBA	2.18E-01	1	0	no
Sicily_MBA	5.77E-01	1	0	no
Sicily_IA	6.95E-02	1	0	yes
Sicily_LBA	2.61E-01	1	0	yes
Sicily_MBA	6.25E-01	1	0	yes

The relationship between the civilians and soldiers of Himera

In most Greek cities the army was composed of citizens who could furnish themselves with armor. The results of the *qpAdm* analysis, as well as the PCA and ADMIXTURE analyses, which involve low coverage individuals, indicate a higher genetic diversity among the civilians than among Aegean-related soldiers (the groups Sicily_Himera_480BCE_1 and Sicily_Himera_409BCE), although not as high as among the genetic outliers of the 480 BCE battle, who likely included foreign mercenaries. To compare the genetic diversity between the groups of Aegean-related soldiers (including both battles) and the civilians of Himera, we used the software PAST 4.04 (49) to conduct a one-way PERMANOVA (50) on the coordinates of the first two PCs, and show that the centroids of the two groups are not significantly different in their position (p=0.218; 9999 permutations, Euclidean similarity index; Fig. S15). However, the variance within groups, represented by the Euclidean distances of points to the centroid of their respective group, is significantly higher in the civilian sample (p=2.51-05; t-test, Exact permutation; Fig. S15). On face value, this suggests that the soldiers might be a less diverse subset of the civilian population, in accordance with the historical hypothesis. However, as the scatter in the civilian sample could be due to noise created by the low coverage of the majority of these individuals, we repeated the tests with 5 iterations of randomly downsampling each soldier to the coverage of one of the civilian individuals (and dropping one soldier to match the number of civilian samples available). After downsampling, the centroids of the soldiers remain not significantly different from the centroid of the civilian group (p>0.140; 9999 permutations, Euclidean similarity index), while the difference in variance becomes non-significant in 4 of 5 cases (p>0.209 in 4 cases, and p=0.024 in one case; t-test, Exact permutation; Fig. S15). This demonstrates how high missingness can make a population appear more genetically heterogeneous than it really is, when only taking PCA into account. However, *qpAdm* results (see above) show that at least one of the civilian individuals, I20163/W1838, has an ancestry composition distinct from what we see in any of the soldiers. Overall, the results do not contradict the hypothesis that the general populace comprised a more diverse ancestry than that represented by Aegean-related soldiers who best represent the descendants of Greek settlers. The possibility that the citizen-soldiers who defended Himera against attack in 409 BCE represent a subset of the population more closely related to “Greek” founders than other citizens warrants further investigation.

Local Sicilian ancestry in the Aegean-related soldiers

We find that for each of the battles the largest groups of soldiers (Sicily_Himera_480BCE_1 and Sicily_Himera_409BCE) are primarily related to the Aegean BA. This inference is based on according to their clustering with Greece_BA on the PCA, similar proportions of genetic components in ADMIXTURE analysis, and valid *qpAdm* models involving Aegean sources. However, these groups do not seem completely genetically homogeneous in the mentioned analyses, as well as in the *qpfmv* result for Sicily_Himera_480BCE_1, and valid *qpAdm* models involve also non-Aegean sources (Table S10). It is possible that the heterogeneous nature of the groups can be explained by genetic substructure in their ancestral Aegean populations, as well as admixture of their ancestors with non-Aegean groups such as local Sicilians. This could be indicated by the position of three of the Sicily_Himera_409BCE individuals shifted more closely to indigenous Sicilian groups on the PCA. Indeed, we do not expect both groups to share the same recent population history, as, according to historical sources, the 480 BCE battle involved supporting armies from other Greek *poleis*, while in the 409 BCE battle Himera was defended by her own inhabitants with no outside support.

Grouped modeling with proximal sources as performed previously might obscure slight differences in individual ancestry. As a simple model, we might interpret the position of the Himeran soldiers on the PCA as a genetic cline between an Aegean-related and an indigenous Sicilian-related source, in which each individual has slightly differing proportions of the two different ancestries. Local admixture between the communities that inhabited the same island and undoubtedly

interacted with each other for decades or even centuries is expected. We therefore first model each individual in Sicily_Himera_480BCE_1 and Sicily_Himera_409BCE as a 2-way mixture between Greece_LBA and a local preceding or contemporaneous indigenous Sicilian group; here we use Sicily_LBA and Sicily_IA. We first confirm that we can distinguish the source proxies with our chosen outgroups (P16 + Greece_Crete_BA, Armenia_LBA.SG, Israel_MLBA_Canaanite, Italy_Sardinia_C_o, Sicily_Himera_Civilians_I20163, Sicily_Himera_Civilians_I20166, Sicily_Himera_Civilians_I20168) using *qpWave* (Table S16). All models of cladality are rejected with p-values <<0.01.

Table S16. *qpWave* results confirming that our outgroups can distinguish between the proposed source populations at a significance level of p < 0.01.

Pop_A	Pop_B	p-value
Greece_LBA	Sicily_IA	4.79E-29
Greece_LBA	Sicily_LBA	1.33E-15
Sicily_LBA	Sicily_IA	5.60E-11

We ran *qpAdm* by adding all unused sources in a given model to the outgroups. In Table S17, we report the results for all 2-way models using Greece_LBA and Sicily LBA or IA as sources. When these are all rejected for a given individual or result in coefficients and standard errors consistent with no ancestry deriving from one of the sources, we additionally test feasible 1-way models. We find among the soldiers of the 480 BCE battle three (I10945/W0494, I10952/W2738 and I7221/W2825) which are best modeled as deriving all of their ancestry from Greece_LBA without added genetic input. For 4 soldiers (I10948/, I7217/W2590 and I7218/W2737 and I7219) the most parsimonious model is an admixture of around 52-86% Greece_LBA and 21-48% Sicily_LBA or Sicily_IA.

Among the soldiers of the 409 BCE battle, one (I17884/W4666) is consistent with descending solely from Greece_LBA. The other 4 are estimated to derive additional 18-64% of their ancestry from Sicily_LBA or Sicily_IA. The results demonstrate a local Sicilian contribution to the ancestry of the majority of Aegean-related soldiers fighting at Himera, suggesting that their ancestors inhabited Sicily for some generations.

Table S17. Admixture proportions estimated by *qpAdm* for 1- and 2-way models for individual Himeran soldiers using Sicily_LBA/IA and Greece_LBA as the proximal sources. Marked in grey are the most parsimonious models for each individual; in cases where two models are not rejected, we chose the one with lower standard errors.

Group	Individual	Admixture Sources		p-value	Ancestry coefficients		Standard errors		Result
		1	2		Anc_1	Anc_2	SE_1	SE_2	
Sicily_Himera_480BCE_1	I10945	Sicily_LBA	Greece_LBA	5.63E-01	-0.12	1.12	0.155	0.155	infeasible, consistent with no Sicily contribution
	I10945	Sicily_IA	Greece_LBA	4.66E-01	-0.063	1.063	0.17	0.17	infeasible, consistent with no Sicily contribution
	I10945	Greece_LBA		5.37E-01	1		0		not rejected
	I10948	Sicily_LBA	Greece_LBA	6.52E-03	0.162	0.838	0.151	0.151	rejected
	I10948	Sicily_IA	Greece_LBA	1.35E-01	0.482	0.518	0.145	0.145	not rejected
	I10952	Sicily_LBA	Greece_LBA	3.22E-01	0.069	0.931	0.161	0.161	consistent with no Sicily contribution
	I10952	Sicily_IA	Greece_LBA	3.44E-01	-0.021	1.021	0.157	0.157	infeasible, consistent with no Sicily contribution
	I10952	Greece_LBA		3.96E-01	1		0		not rejected
	I7217	Sicily_LBA	Greece_LBA	2.03E-01	0.212	0.788	0.152	0.152	not rejected
	I7217	Sicily_IA	Greece_LBA	2.75E-01	0.317	0.683	0.159	0.159	not rejected
	I7218	Sicily_LBA	Greece_LBA	2.82E-01	0.127	0.873	0.129	0.129	consistent with no Sicily contribution
	I7218	Sicily_IA	Greece_LBA	6.52E-01	0.383	0.617	0.143	0.143	not rejected
	I7219	Sicily_LBA	Greece_LBA	1.34E-02	0.179	0.821	0.184	0.184	consistent with no Sicily contribution
	I7219	Sicily_IA	Greece_LBA	5.87E-02	0.415	0.585	0.151	0.151	not rejected
	I7221	Sicily_LBA	Greece_LBA	4.97E-01	-0.103	1.103	0.14	0.14	infeasible, consistent with no Sicily contribution
	I7221	Sicily_IA	Greece_LBA	4.63E-01	-0.036	1.036	0.151	0.151	infeasible, consistent with no Sicily contribution
	I7221	Greece_LBA		5.72E-01	1		0		not rejected
Sicily_Himera_409BCE	I17866	Sicily_IA	Greece_LBA	5.39E-01	0.182	0.818	0.168	0.168	not rejected
	I17884	Sicily_LBA	Greece_LBA	1.97E-01	-0.124	1.124	0.357	0.357	infeasible, consistent with no Sicily contribution
	I17884	Sicily_IA	Greece_LBA	1.72E-01	-0.058	1.058	0.358	0.358	infeasible, consistent with no Sicily contribution
	I17884	Greece_LBA		2.03E-01	1		0		not rejected
	I7223	Sicily_LBA	Greece_LBA	8.32E-02	0.243	0.757	0.121	0.121	not rejected
	I7223	Sicily_IA	Greece_LBA	4.53E-01	0.458	0.542	0.139	0.139	not rejected
	I7224	Sicily_LBA	Greece_LBA	4.45E-01	0.129	0.871	0.128	0.128	not rejected
	I7224	Sicily_IA	Greece_LBA	3.92E-01	0.153	0.847	0.145	0.145	not rejected
	I7225	Sicily_LBA	Greece_LBA	2.42E-01	0.644	0.356	0.122	0.122	not rejected
	I7225	Sicily_IA	Greece_LBA	3.04E-01	0.509	0.491	0.129	0.129	not rejected

Runs of homozygosity and population size

We used the hidden Markov model algorithm implemented in the software package hapROH (51) to detect ROH, which is possible for samples covered at more than 400,000 SNPs (Fig. S14 and Table S18).

Fewer than half of the individuals exhibit any ROH, indicating a large effective population size (51). Endogamous practices are unlikely, as we find no evidence of recent inbreeding between close relatives, and most individuals do not carry ROH over 4 cM in length. However, in all groups except Sicily_Himera_480BCE_2 and Sicily_Himera_409BCE some of the individuals carry a low amount of ROH in the length range of 4-20 cM. This could signify consanguinity between very distantly related parents which can happen by chance, especially if no cultural institutions are in place to prohibit unions between even distant relatives, or if genetic substructure existed among the population of Sicily.

Table S18. Distribution of Runs of Homozygosity >4cM

Lab ID	Group Label	Sum total of ROH segments >4cM	Sum total of ROH segments >20cM
**I10371**	Italy_Sicily_LBA	n/a (too few SNPs)	n/a (too few SNPs)
**I10372**	Italy_Sicily_LBA	15.487098	0
**I3876**	Italy_Sicily_LBA	5.845797	0
**I3878**	Italy_Sicily_LBA	0	0
**I10373**	Italy_Sicily_LBA_son.I3878	5.362302	0
**I17866**	Sicily_Himera_409BCE	0	0
**I17884**	Sicily_Himera_409BCE	n/a (too few SNPs)	n/a (too few SNPs)
**I7223**	Sicily_Himera_409BCE	0	0
**I7225**	Sicily_Himera_409BCE	0	0
**I7224**	Sicily_Himera_409BCE	0	0
**I7217**	Sicily_Himera_480BCE_1	20.644401	0
**I7219**	Sicily_Himera_480BCE_1	18.95811	0
**I7218**	Sicily_Himera_480BCE_1	17.690196	0
**I7221**	Sicily_Himera_480BCE_1	0	0
**I10945**	Sicily_Himera_480BCE_1	0	0
**I10952**	Sicily_Himera_480BCE_1	0	0
**I10948**	Sicily_Himera_480BCE_1	0	0
**I10946**	Sicily_Himera_480BCE_2	0	0
**I10950**	Sicily_Himera_480BCE_2	0	0
**I10943**	Sicily_Himera_480BCE_3	5.178202	0
**I10949**	Sicily_Himera_480BCE_3	4.426599	0
**I10947**	Sicily_Himera_480BCE_4	4.1877	0
**I10944**	Sicily_Himera_480BCE_4	0	0
**I10951**	Sicily_Himera_480BCE_5	7.792598	0
**I17878**	Sicily_Himera_Civilians	n/a (too few SNPs)	n/a (too few SNPs)
**I17879**	Sicily_Himera_Civilians	n/a (too few SNPs)	n/a (too few SNPs)
**I17887**	Sicily_Himera_Civilians	n/a (too few SNPs)	n/a (too few SNPs)
**I17881**	Sicily_Himera_Civilians	n/a (too few SNPs)	n/a (too few SNPs)
**I17432**	Sicily_Himera_EastNecropolis	5.397784	0
**I13133**	Sicily_IA	n/a (too few SNPs)	n/a (too few SNPs)
**I13140**	Sicily_IA	n/a (too few SNPs)	n/a (too few SNPs)
**I13142**	Sicily_IA	n/a (too few SNPs)	n/a (too few SNPs)
**I13125**	Sicily_IA	n/a (too few SNPs)	n/a (too few SNPs)
**I13128**	Sicily_IA	n/a (too few SNPs)	n/a (too few SNPs)
**I13390**	Sicily_IA	16.812196	0
**I13376**	Sicily_IA	9.60151	0
**I13393**	Sicily_IA	7.635796	0
**I13384**	Sicily_IA	6.7605	0
**I13378**	Sicily_IA	5.289501	0
**I13389**	Sicily_IA	4.4148	0
**I13387**	Sicily_IA	0	0
**I13392**	Sicily_IA	0	0
**I13394**	Sicily_IA	0	0
**I13377**	Sicily_IA	0	0
**I13395**	Sicily_IA	0	0
**I13379**	Sicily_IA	0	0
**I13380**	Sicily_IA	0	0
**I13381**	Sicily_IA	0	0
**I13391**	Sicily_IA	0	0
**I13382**	Sicily_IA	0	0
**I13383**	Sicily_IA	0	0
**I13385**	Sicily_IA	0	0
**I13386**	Sicily_IA	0	0

Phenotypic analysis

We used the HIrisPlex-S system (52-54) to predict the pigmentation of individuals included in our study. Individuals with at least one SNP of the system with data could be submitted to HIrisPlex-S for phenotype prediction, and discussion is limited to the individuals for which the system returned inference probabilities for the different phenotypes (n/a was returned if the SNPs were insufficient for a prediction to be made).

We followed the procedure of a previous publication (55) in simulating genotypes (10 times per individual) from genotype likelihoods at each SNP locus and the prior probability of the allele frequency of each SNP (Dataset S7). The simulated genotypes were submitted to the HIrisPlex-S website (https://hirisplex.erasmusmc.nl/) on Dec. 7, 2020. Results across the 10 replicates were averaged and phenotype predictions were made for the hair, eye, and skin color phenotypes based on these averages (HairSimple: “light” or “dark”, HairDetailed: “red”, “blond”, “brown”, “black”, Eye: “blue”, “intermediate”, “brown”, and Skin color: “very pale”, “pale”, “intermediate”, “dark”, “dark-to-black”). We find a prevalence of brown hair, brown eyes and intermediate complexion among the studied individuals (Table S19).

We also screened the data for the derived alleles of *MCM6* at locus rs4988235 and *EDAR* at locus rs3827760. We find the presence of the derived alleles in two individuals.

We find 4 in 6 of the sequences covering the *MCM6* locus in the individual I10943/W0396 carry the derived allele, indicating that the individual is heterozygous. *MCM6* is a regulatory gene that affects the lactase *LCT* gene and rs3827760 (A>G) is responsible for lactase persistence, the ability to digest milk into adulthood, in the majority of Europeans with this trait (56, 57). It is hypothesized that this allele originated among Steppe pastoralists and spread in to Europe with their migration at the transition from Neolithic to Bronze Age (58). The lactase persistence trait was under positive selection and the frequency of the allele started to increase rapidly towards the end of the Bronze Age (59). In the eastern Baltic, a sharp increase is already seen at the end of the Late Neolithic with the variant reaching a frequency over 50% in the 1st millennium BCE (44, 45), while the variant remained less common further south in Europe and its distribution still follows a decreasing north to south cline to this day (60). This observation of the derived allele in I10943/W0396 agrees with his genetic ancestry, with the greatest affinity to BA and IA Eastern Baltic groups. Notably, this individual also has an uncommon physical appearance among the studied samples, with Hirisplex-S estimating highest probabilities for a combination of blond hair, blue eyes and pale skin. This phenotype also rose in frequency in the Eastern Baltic in the 1st millennium BCE (44, 45).

The derived *EDAR* allele is found in 1 of 4 of the reads covering the locus in the individual I10944/W0461. The variant affects tooth morphology and hair texture and thickness and is found in high frequencies only in modern-day East Asians and Native Americans (61-63). This result corresponds with the findings that I10944/W0461 shares the highest genetic affinity with IA steppe nomads who for the most part carry East Asian ancestry (46-48), and that this individual’s ancestry can only be modeled when including a South Siberian source (Table S12).

Table S19. Phenotypic information on Sicilian individuals. Hirsiplex-S predictions are based on the highest probability of ten replicates. Samples with evidence of carrying the derived *EDAR* or *MCM6* allele are marked in grey.

		**HIrisplex-S prediction**				**Read depth (derived allele proportion)**	
**Group**	**Sample**	**Eye**	**HairDetailed**	**HairSimple**	**Skin**	***EDAR* s3827760 (A>G)**	***MCM6* rs4988235 (G>A)**
Sicily_IA	I13125	Brown	Brown	Dark	NA	7 (0%)	3 (0%)
	I13128	NA	NA	NA	Intermediate	2 (0%)	2 (0%)
	I13376	Brown	Brown	Dark	Intermediate	9 (0%)	13 (0%)
	I13377	Brown	Brown	Dark	Dark	8 (0%)	13 (0%)
	I13378	Brown	Black	Dark	Dark	8 (0%)	13 (0%)
	I13379	NA	Brown	Dark	Intermediate	NA	1 (0%)
	I13380	Brown	Black	Dark	Dark	7 (0%)	7 (0%)
	I13381	Brown	Brown	Dark	Intermediate	6 (0%)	11 (0%)
	I13383	Brown	Black	Dark	Intermediate	11 (0%)	15 (0%)
	I13384	Brown	Brown	Dark	Intermediate	7 (0%)	3 (0%)
	I13385	Brown	Brown	Dark	Intermediate	19 (0%)	19 (0%)
	I13386	Brown	Black	Dark	Intermediate	8 (0%)	8 (0%)
	I13387	Brown	Black	Dark	Intermediate	3 (0%)	7 (0%)
	I13389	Brown	Brown	Light	Intermediate	9 (0%)	7 (0%)
	I13390	Brown	Brown	Dark	Dark	9 (0%)	6 (0%)
	I13391	Brown	Brown	Dark	Intermediate	8 (0%)	15 (0%)
	I13392	Brown	Black	Dark	Dark	12 (0%)	9 (0%)
	I13393	Blue	Blond	Light	Intermediate	3 (0%)	8 (0%)
	I13394	Brown	Brown	Dark	Intermediate	8 (0%)	15 (0%)
	I13395	Brown	Brown	Dark	Intermediate	3 (0%)	5 (0%)
Sicily_Himera_480BCE_1	I10945	Brown	Black	Dark	Intermediate	14 (0%)	15 (0%)
	I10948	Brown	Black	Dark	Dark	8 (0%)	10 (0%)
	I10952	Brown	Red	Light	Intermediate	10 (0%)	10 (0%)
	I7217	Brown	Brown	Light	Intermediate	1 (0%)	4 (0%)
	I7218	Brown	Brown	Dark	Intermediate	6 (0%)	7 (0%)
	I7219	Brown	Brown	Dark	Intermediate	14 (0%)	11 (0%)
	I7221	NA	Blond	Light	Intermediate	15 (0%)	17 (0%)
Sicily_Himera_480BCE_2	I10946	NA	Brown	Light	Intermediate	5 (0%)	5 (0%)
	I10950	Brown	Brown	Light	Intermediate	22 (0%)	13 (0%)
Sicily_Himera_480BCE_3	I10943	Blue	Blond	Light	Pale	14 (0%)	6 (67%)
	I10949	Brown	Brown	Dark	Intermediate	1 (0%)	2 (0%)
Sicily_Himera_480BCE_4	I10944	Brown	Brown	Dark	Pale	4 (25%)	8 (0%)
	I10947	Brown	Brown	Dark	Intermediate	15 (0%)	8 (0%)
Sicily_Himera_480BCE_5	I10951	Brown	Brown	Dark	Intermediate	9 (0%)	3 (0%)
Sicily_Himera_409BCE	I17866	NA	NA	NA	Intermediate	NA	1 (0%)
	I7223	Brown	Brown	Dark	Dark	14 (0%)	15 (0%)
	I7224	Brown	Brown	Dark	Intermediate	7 (0%)	14 (0%)
	I7225	Brown	Brown	Dark	Intermediate	10 (0%)	8 (0%)
Sicily_Himera_Civilians	I17881	NA	NA	NA	NA	1 (0%)	NA
	I20166	Brown	NA	Light	NA	1 (0%)	4 (0%)
Sicily_Himera_EastNecropolis	I17432	NA	Black	Dark	Intermediate	5 (0%)	2 (0%)

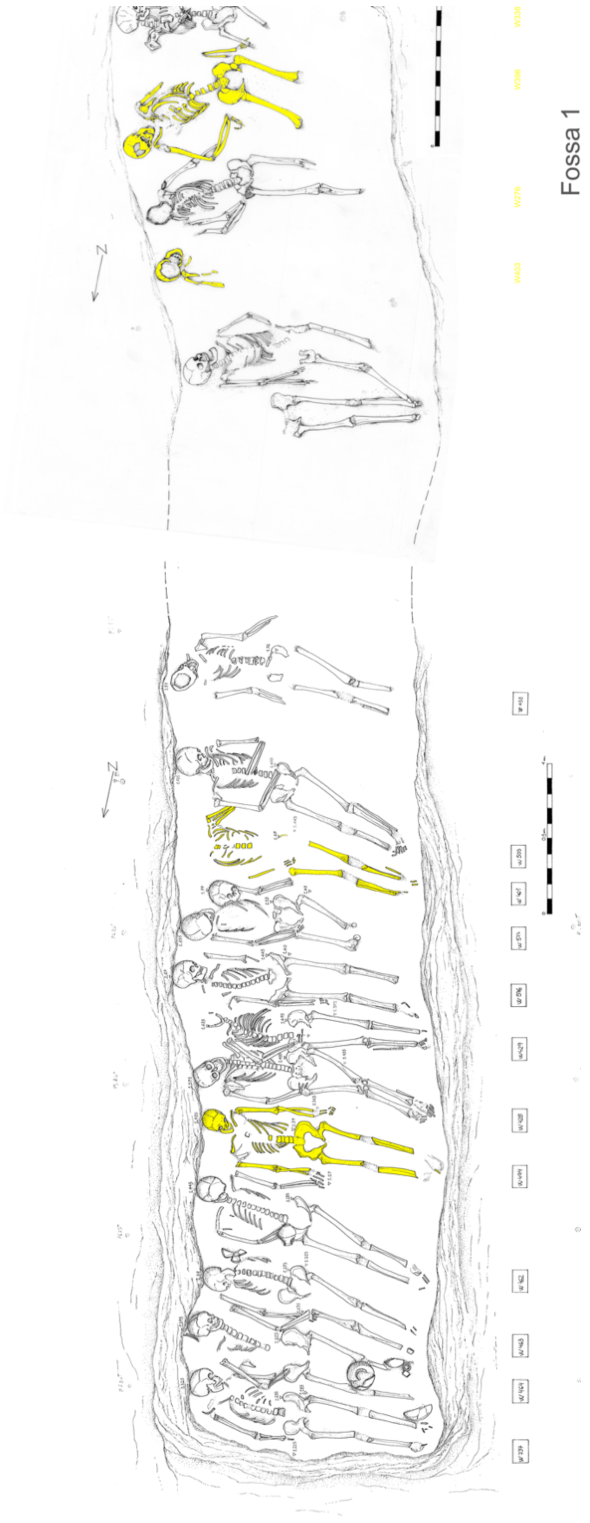

Figure S1. Mass graves #1/2 in the West necropolis of Himera, indicating individuals genetically analyzed in yellow.

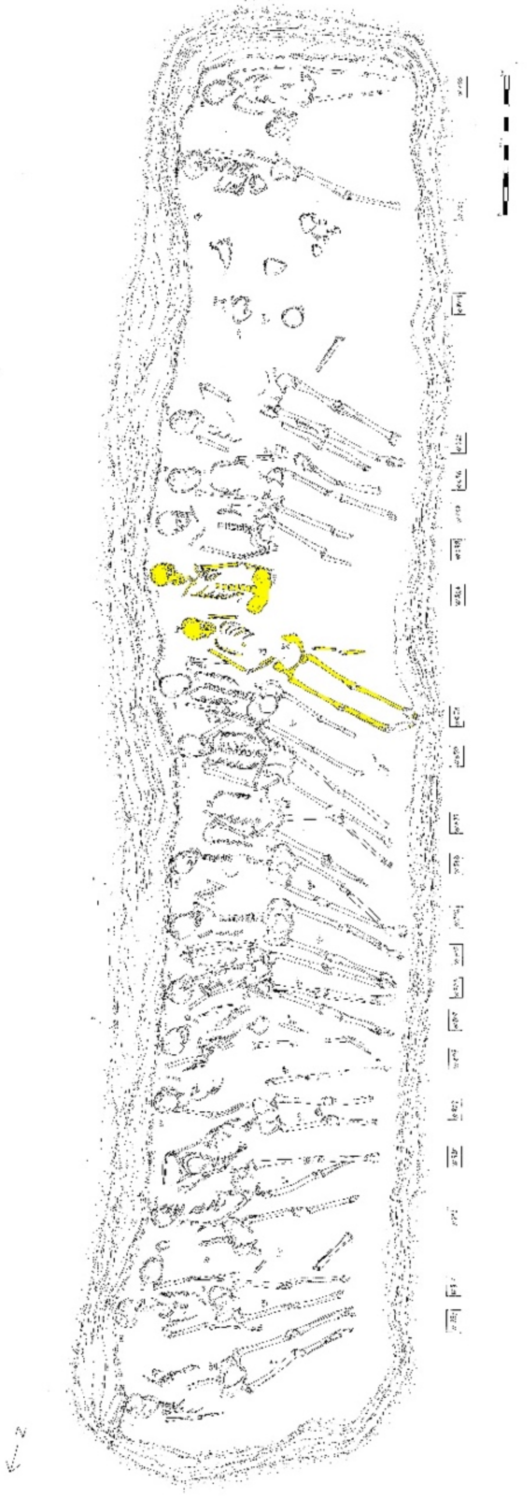

Figure S2. Mass grave #3 in the West necropolis of Himera, indicating individuals genetically analyzed in yellow.

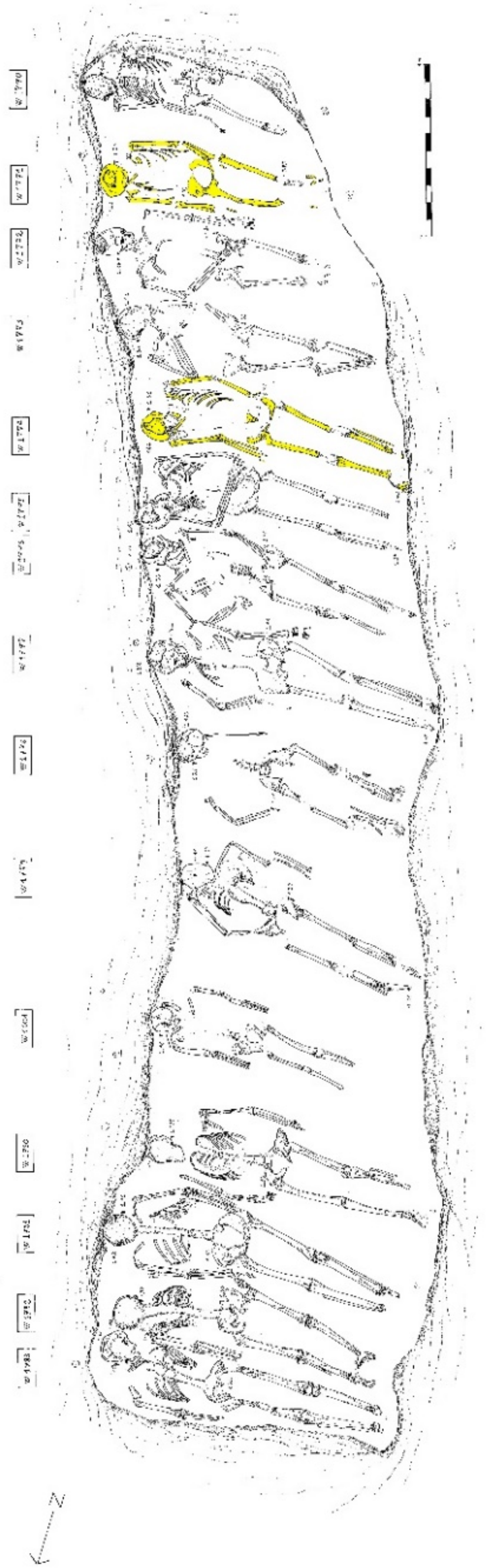

Figure S3. Mass grave #4 in the West necropolis of Himera, indicating individuals genetically analyzed in yellow.

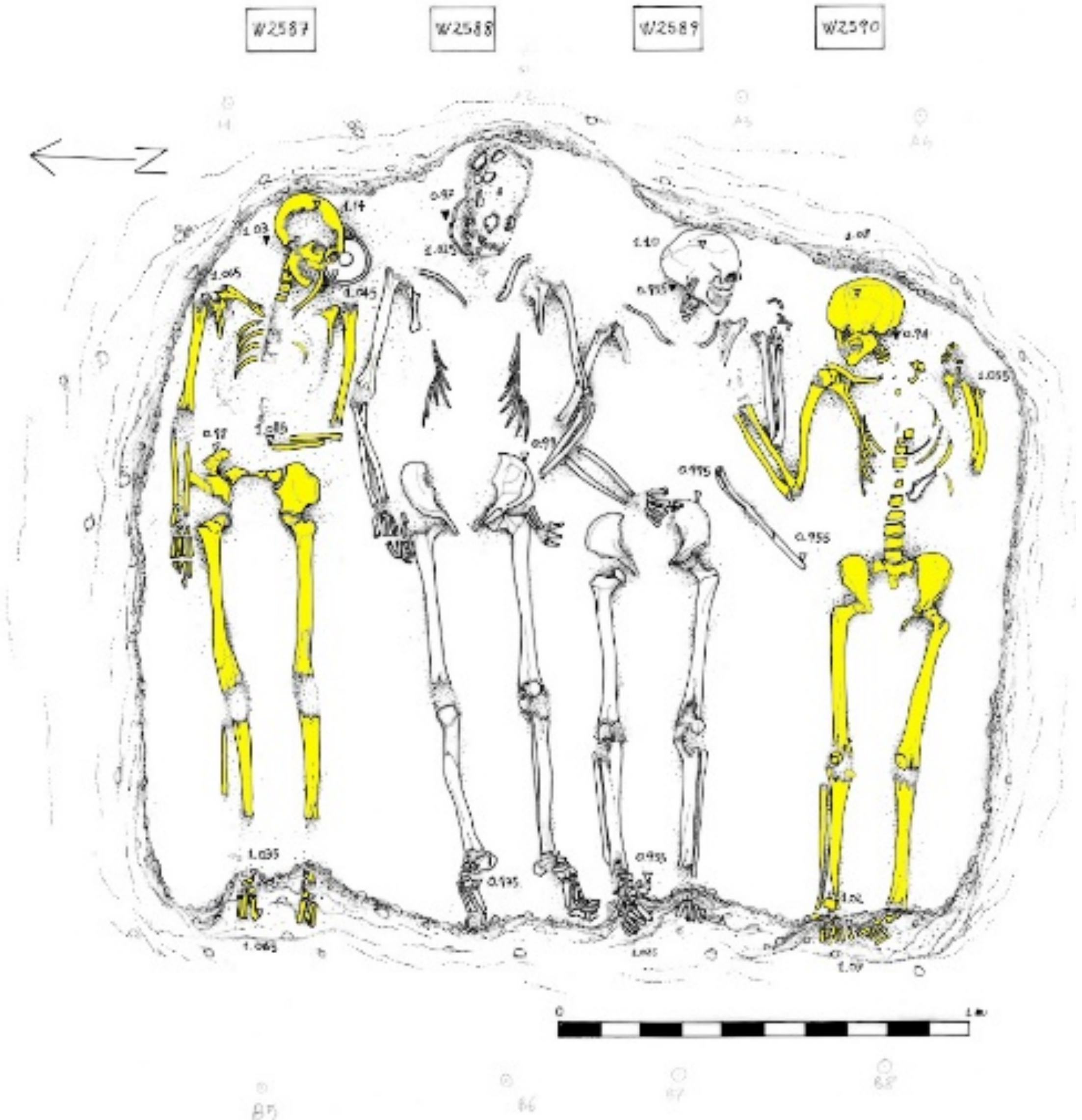

Figure S4. Mass grave #5 in the West necropolis of Himera, indicating individuals genetically analyzed in yellow.

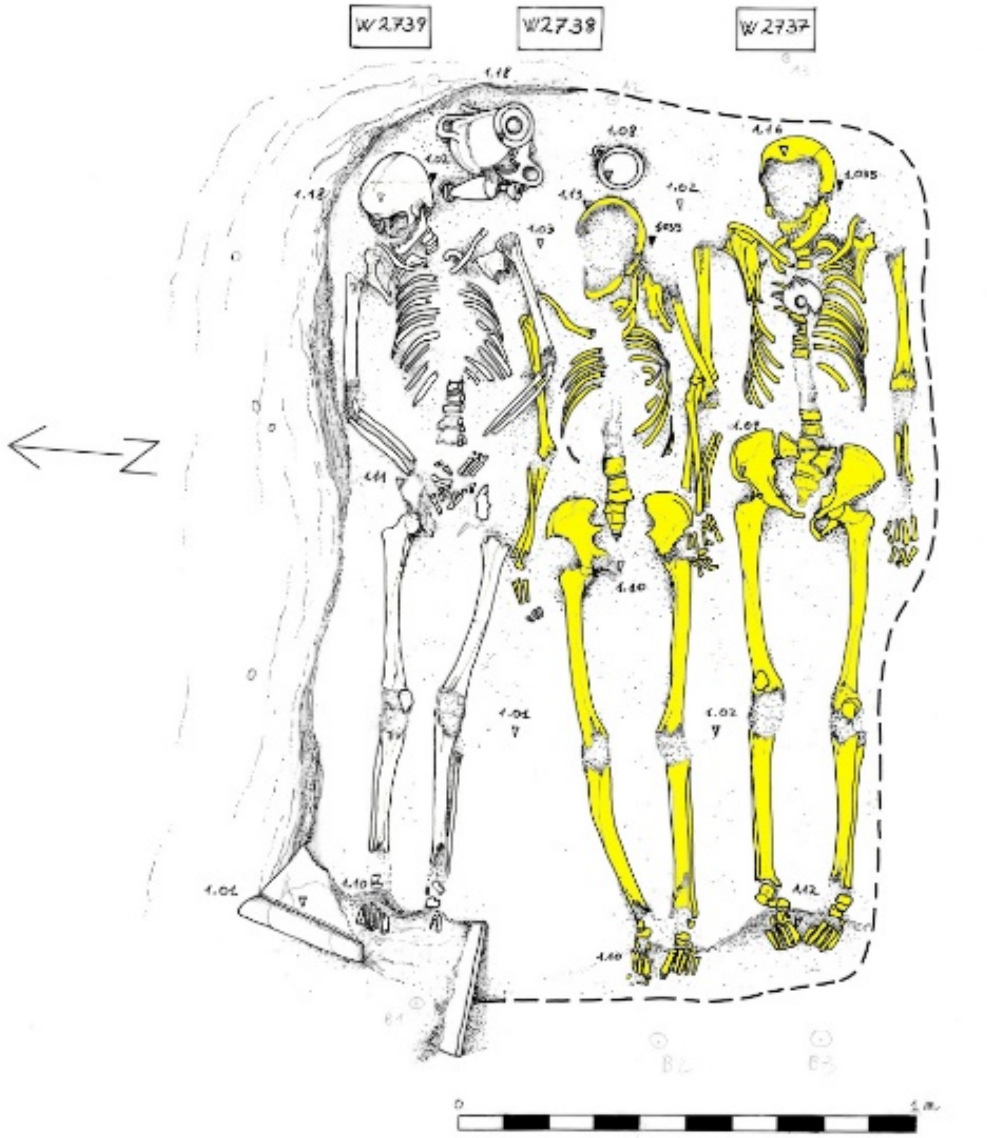

Figure S5. Mass grave #6 in the West necropolis of Himera, indicating individuals genetically analyzed in yellow.

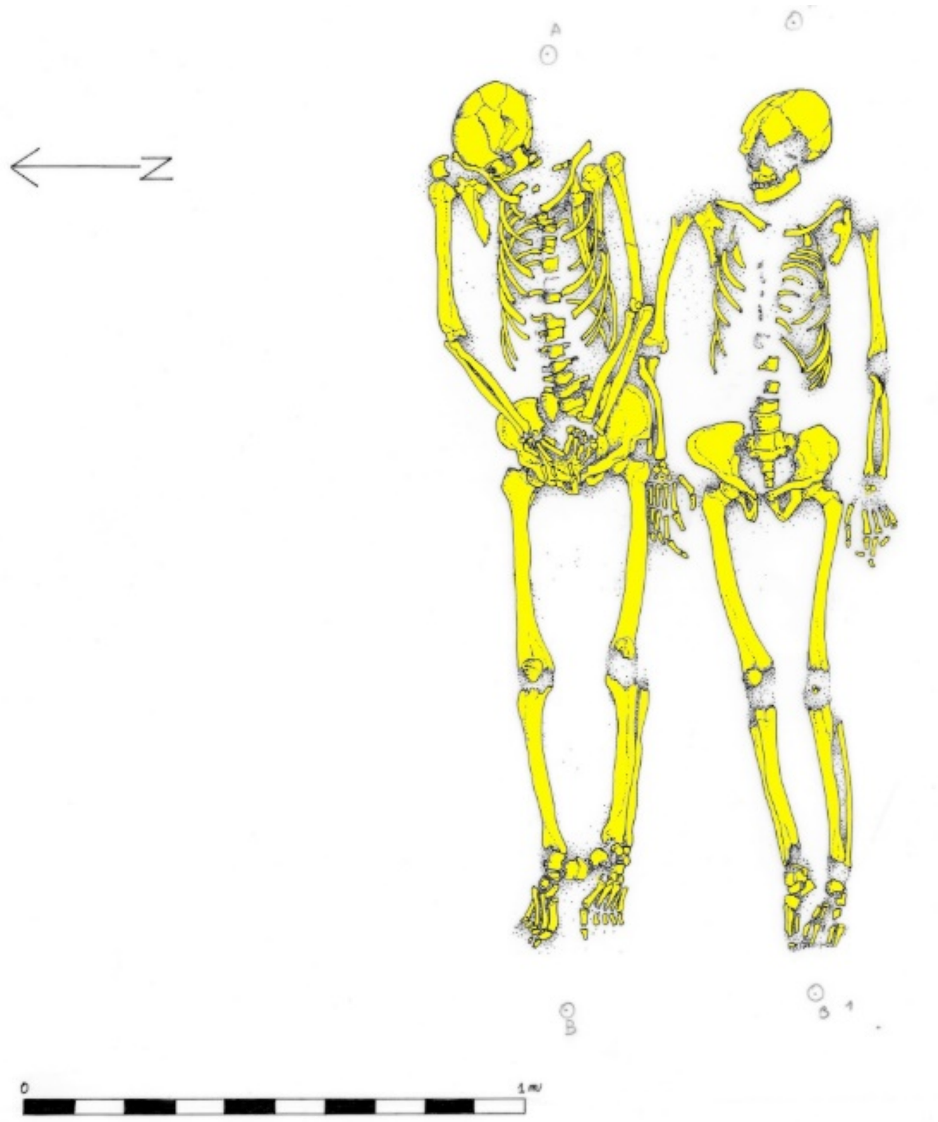

Figure S6. Mass grave #7 in the West necropolis of Himera, indicating individuals genetically analyzed in yellow.

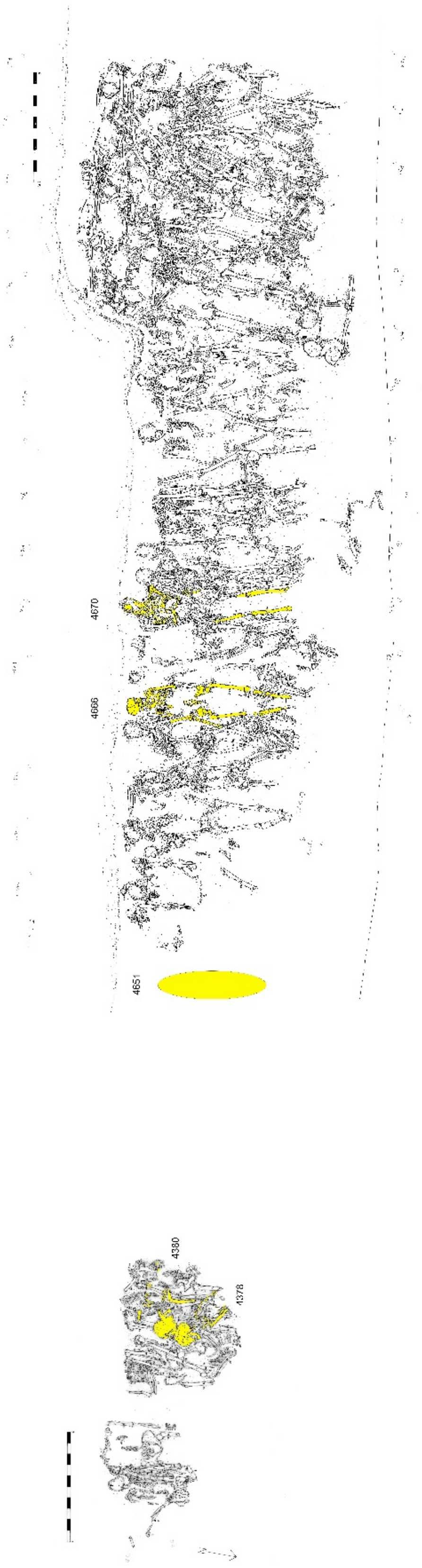

Figure S7. Mass graves #8/9 in the West necropolis of Himera, indicating individuals genetically analyzed in yellow. Individual 4651 was disinterred prior to mapping; its approximate location is indicated in yellow.

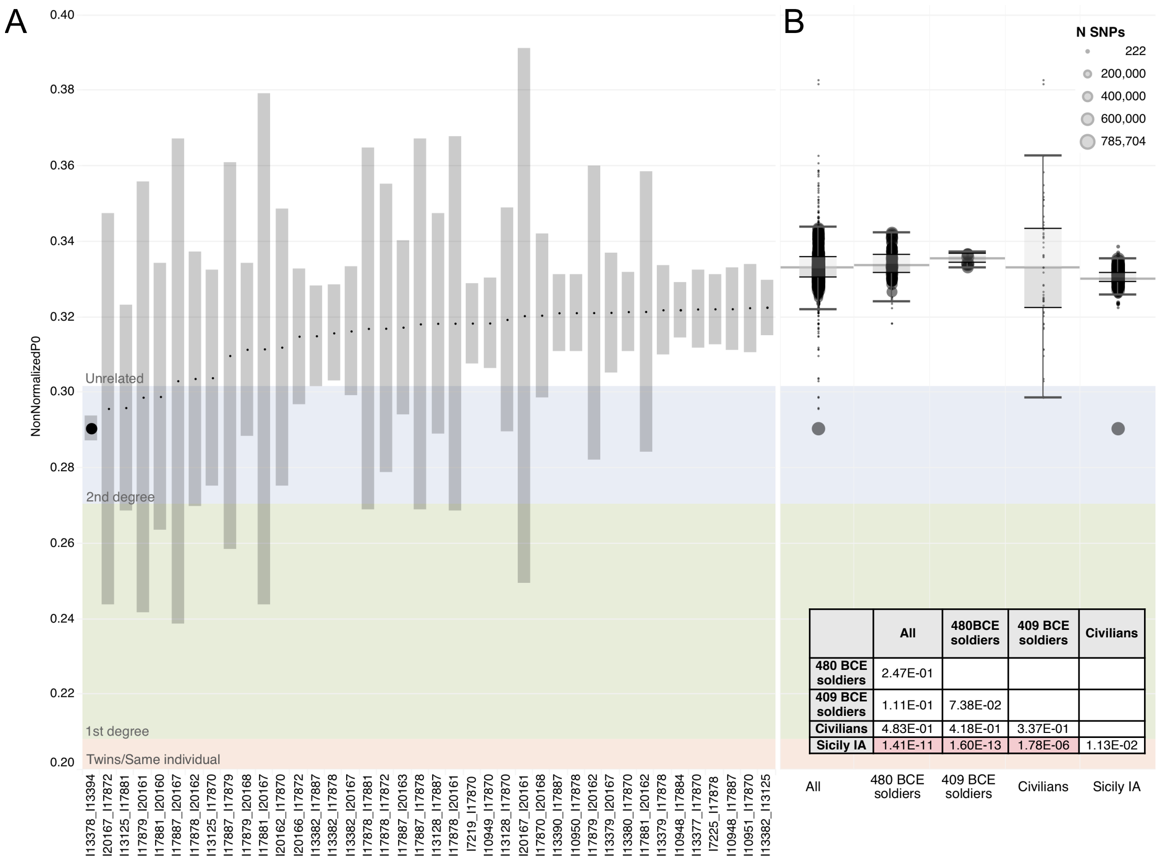

	All	480BCE soldiers	409 BCE soldiers	Civilians
480 BCE soldiers	2.47E-01			
409 BCE soldiers	1.11E-01	7.38E-02		
Civilians	4.83E-01	4.18E-01	3.37E-01	
Sicily IA	1.41E-11	1.60E-13	1.78E-06	1.13E-02

Figure S8. READ results. *(A)* 40 pairs of individuals with the lowest P0 values, gray bars represent 2SE and red, green and blue reference bands represent expected values for twins, 1st degree and 2nd degree relatives, respectively. *(B)* Variance of PO within population groups, whisker plots showing mean and IQR, whiskers extending to 1.5 IQR. Table inset shows p-values for pairwise one-sided T-tests with significant values after Bonferroni correction shaded red.

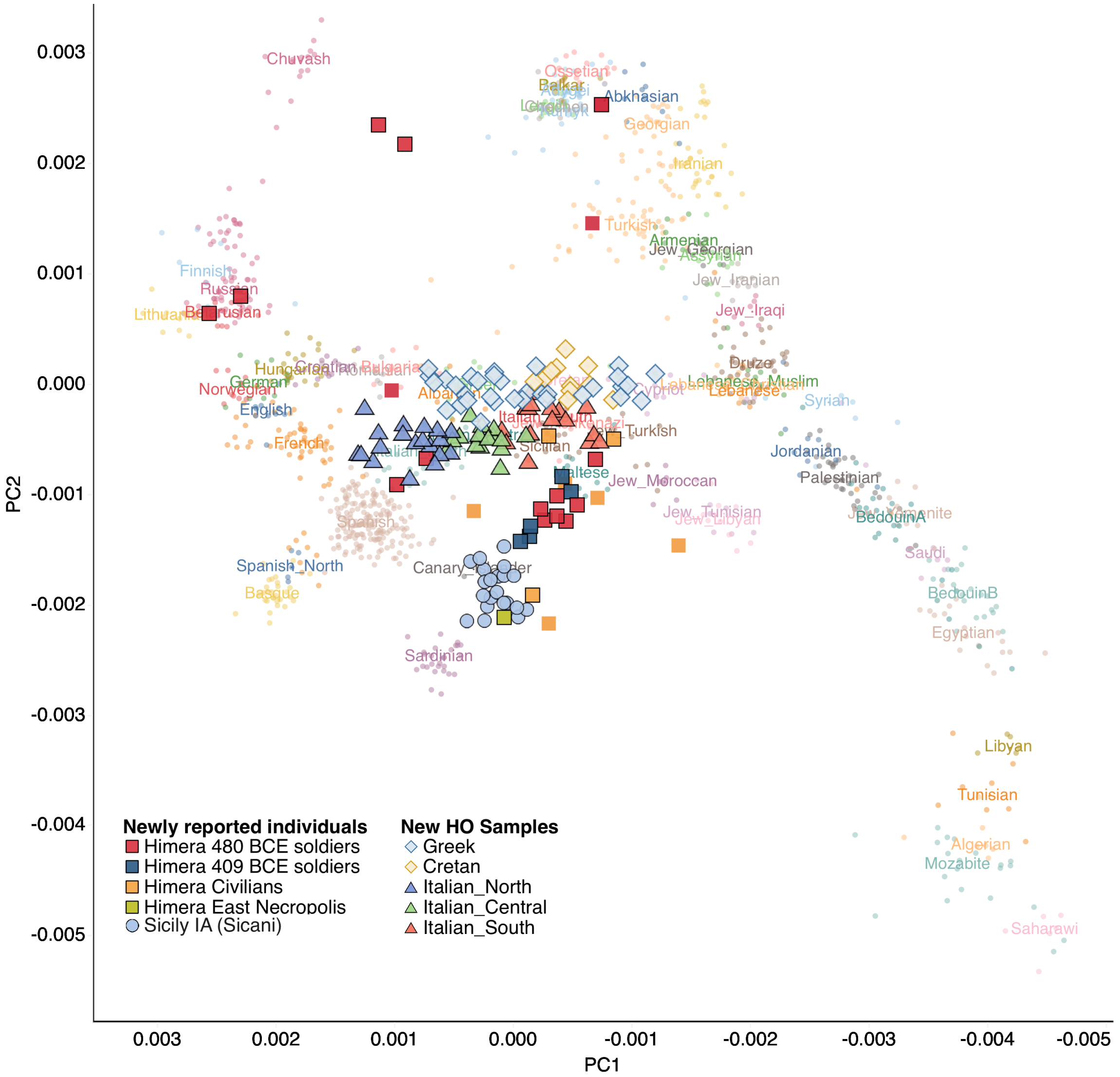

Figure S9. PCA showing modern-day West Eurasian and North African individuals and projected new ancient individuals. Individual points and population labels centered on the population median in corresponding colors.

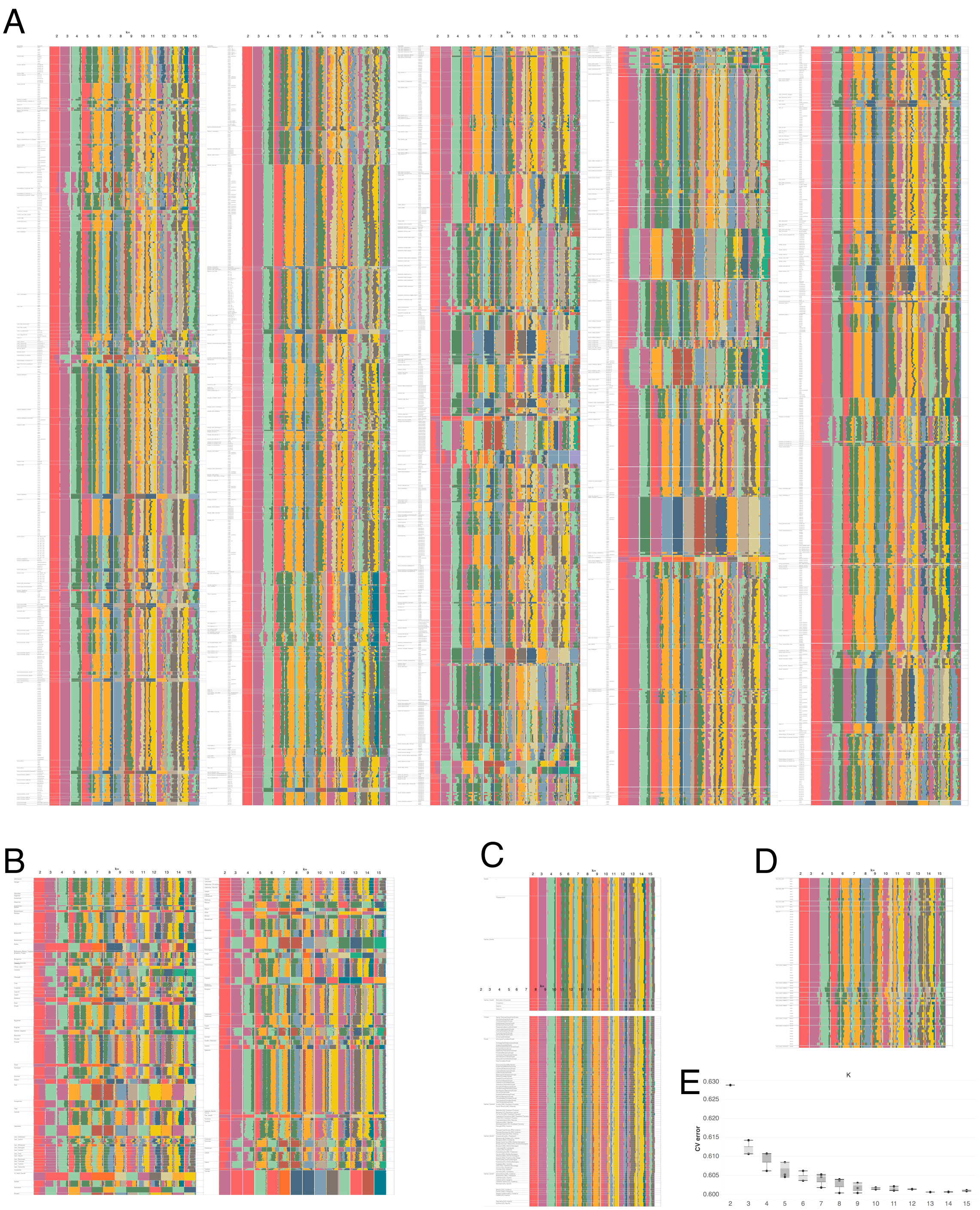

Figure S10. Full results of unsupervised ADMIXTURE for k=2 to k=15. Ancestry components k as bar plots for published ancient individuals (A), present-day individuals (B), present-day published (top) and newly reported individuals from Greece and Italy (C) and ancient Sicilian genomes (D). Cross validation errors for 5 runs per number of k (E).

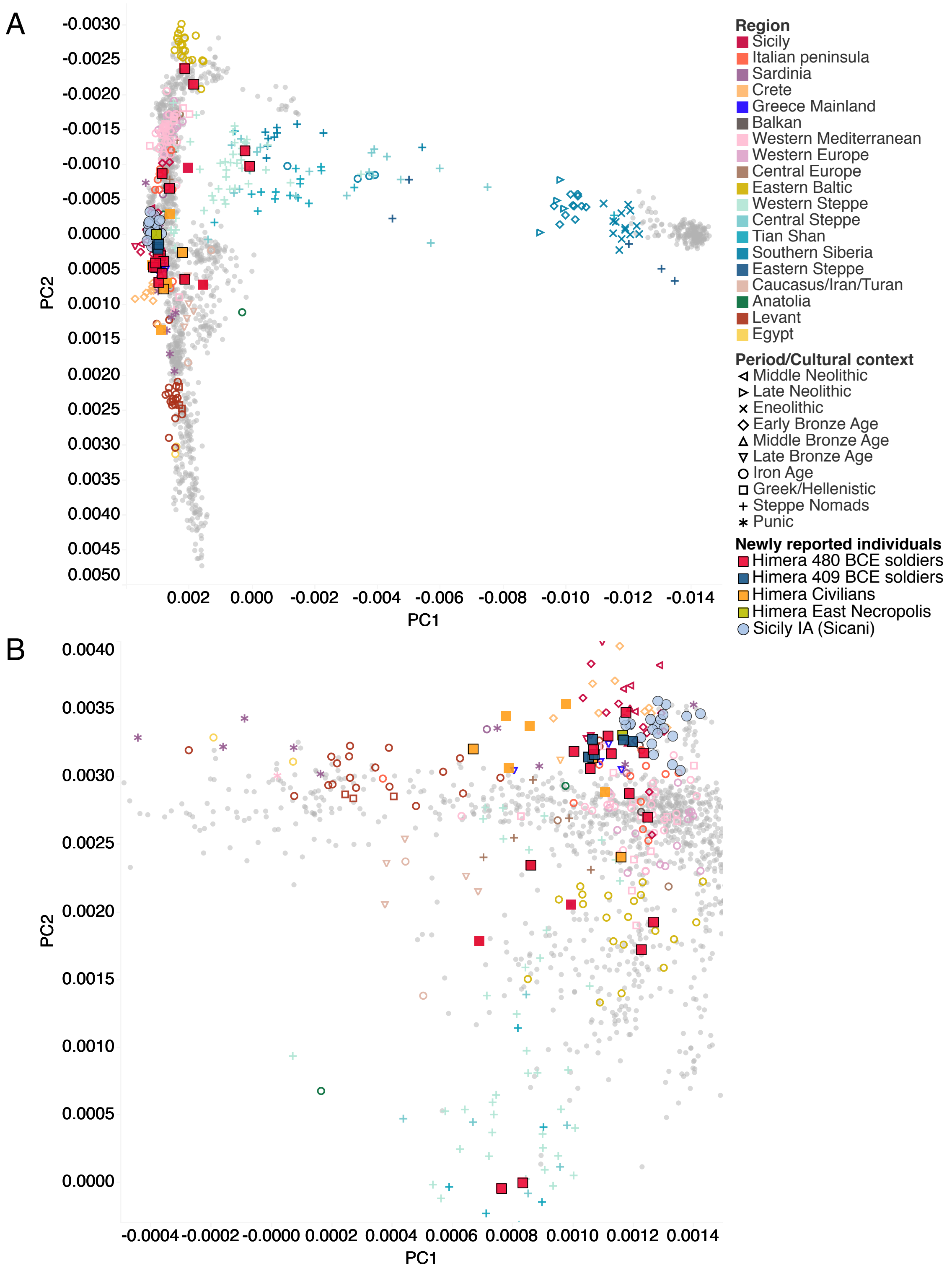

Figure S11. Eurasian and worldwide PCA. PCA constructed using 1390 Eurasian *(A)* and 1900 modern-day worldwide *(B)* individuals, with projected new ancient individuals. Worldwide PCA is zoomed in on the West Eurasian populations.

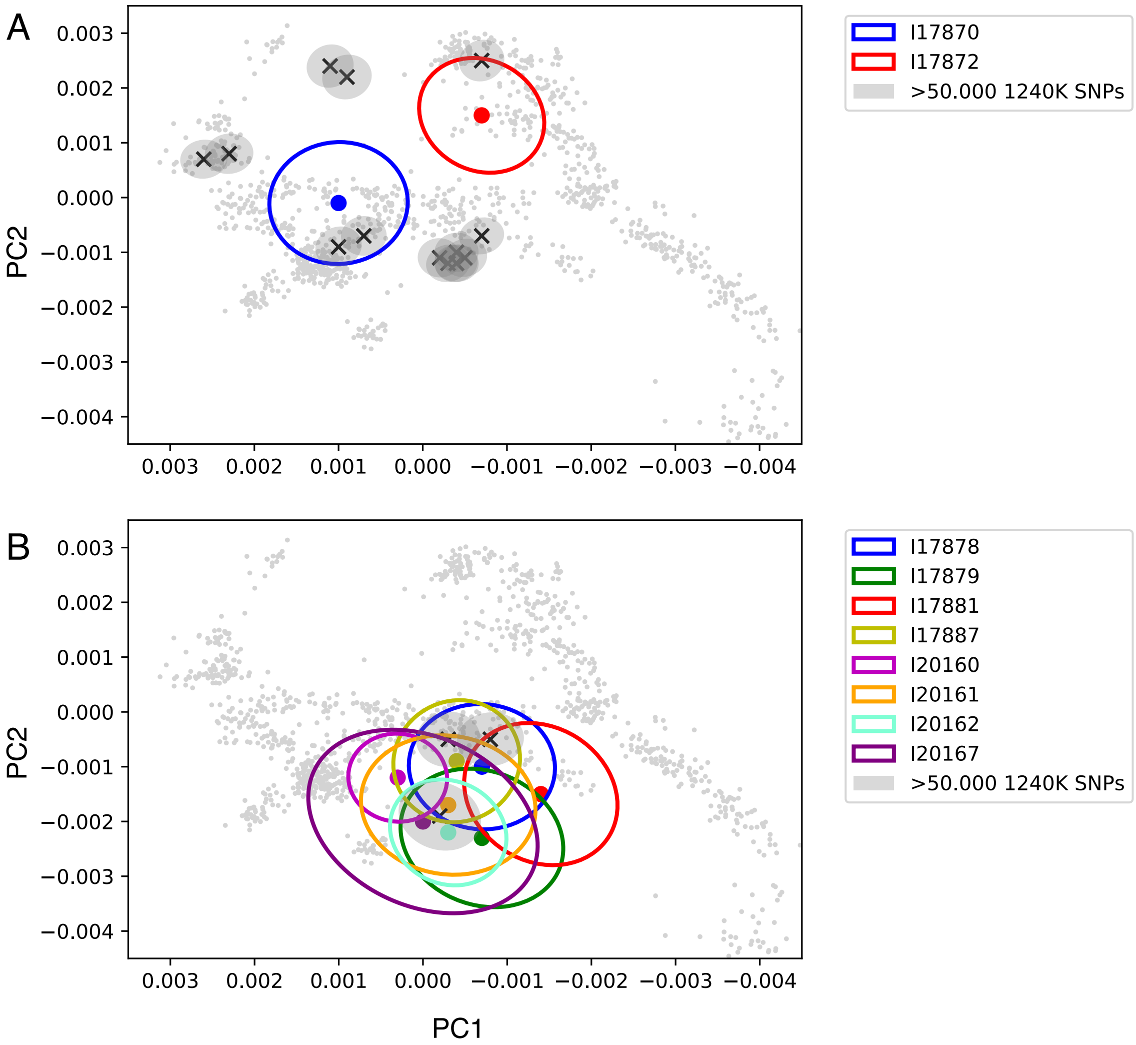

Figures S12. 95% CI ellipses for the PCA projection of low-coverage samples. PC axes computed on modern-day West Eurasian and North African individuals. Shown for 2 samples with fewer than 50,000 SNPs overlapping the 1240K SNP set and 14 samples with more than 50,000 SNPs covered associated with the 480 BCE battle (A), and for 8 civilians with fewer than 50,000 SNPs overlapping the 1240K SNP set in relation to 3 higher coverage civilians (B). Low coverage samples shown as colored outlines with shaded ellipses, high coverage samples as shaded gray ellipses.

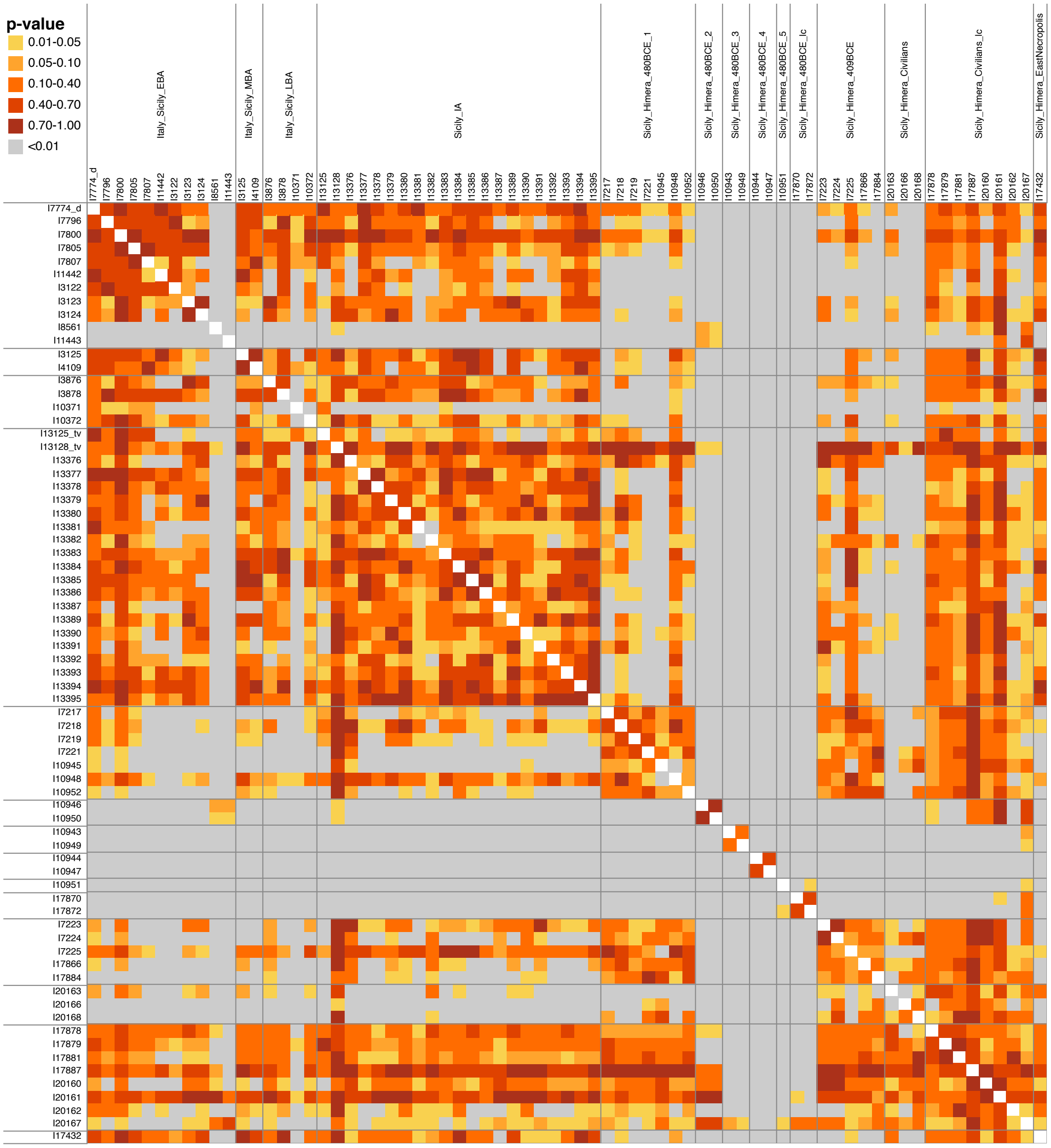

Figure S13. *qpWave* tests for pairs of individuals to assess genetic homogeneity within and between archaeological groupings. Group labels are given at the top, grey fields represent rejected models with p-values below 0.01.

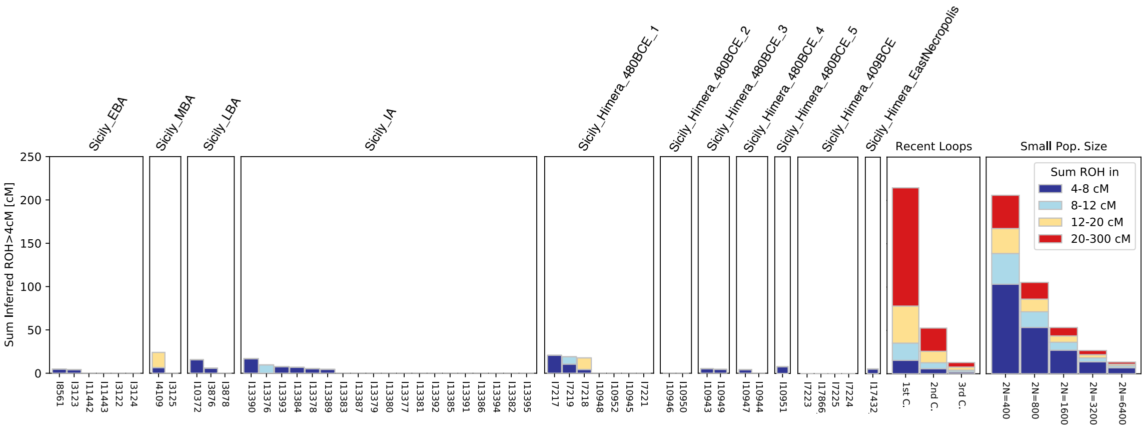

Figure S14. ROH in ancient Sicilians and Himeran soldiers. Each individual is represented by stacked vertical bars, where the length of each bar is determined by the ROH of this individual falling into four length classes (4-8, 8-12, 12-20, and >20 cM, color-coded). The two right-most panels show the expected ROH for offspring of closely related parents and small population sizes, respectively.

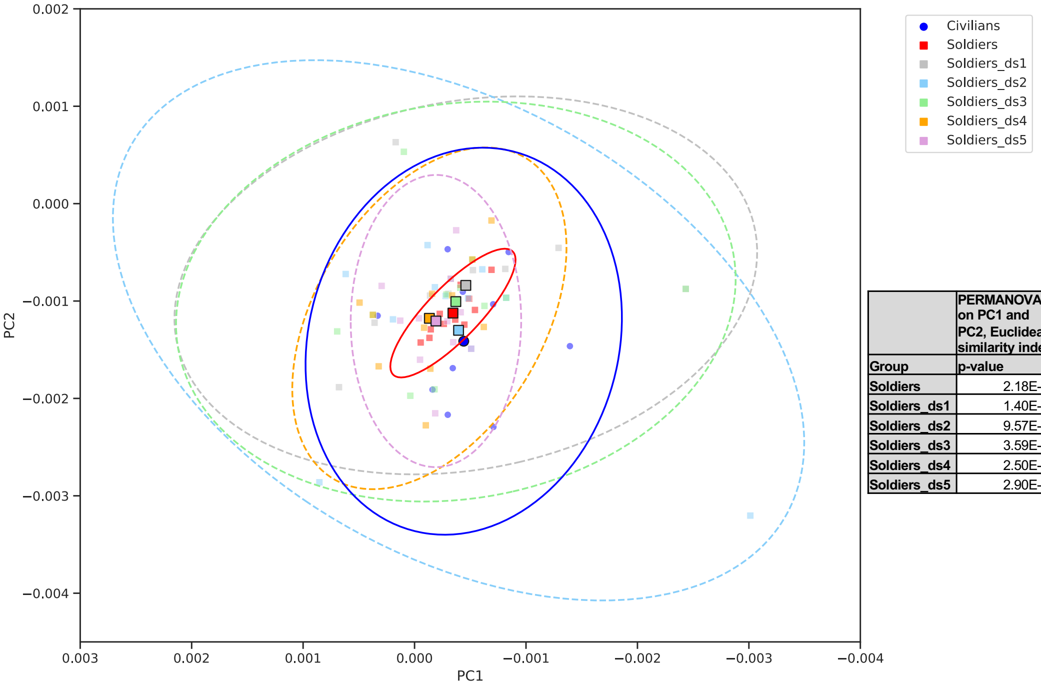

	PERMANOVA on PC1 and PC2, Euclidean similarity index	T-test on distance to centroid of group
**Group**	**p-value**	**p-value**
**Soldiers**	2.18E-01	2.51E-05
**Soldiers_ds1**	1.40E-01	7.30E-01
**Soldiers_ds2**	9.57E-01	4.79E-01
**Soldiers_ds3**	3.59E-01	6.67E-01
**Soldiers_ds4**	2.50E-01	2.09E-01
**Soldiers_ds5**	2.90E-01	2.40E-02

Figure S15. 95% CI ellipses on PC1 and PC2 of the PCA projection of Himera's civilians and Aegean-related soldiers. Coordinates shown for original coverage and 5 iterations of random downsampling on the coverage of the Aegean-related soldiers. Group centroids shown as outlined symbol, 95% CI ellipses of original coverage and downsampled group shown with solid and dashed lines, respectively. Table inset shows p-values for a PERMANOVA of PC1 and PC2 between civilian group and soldiers, and p-values for a T-test of the individual distances to the group centroid. Significant p-values of <0.05 shaded red.

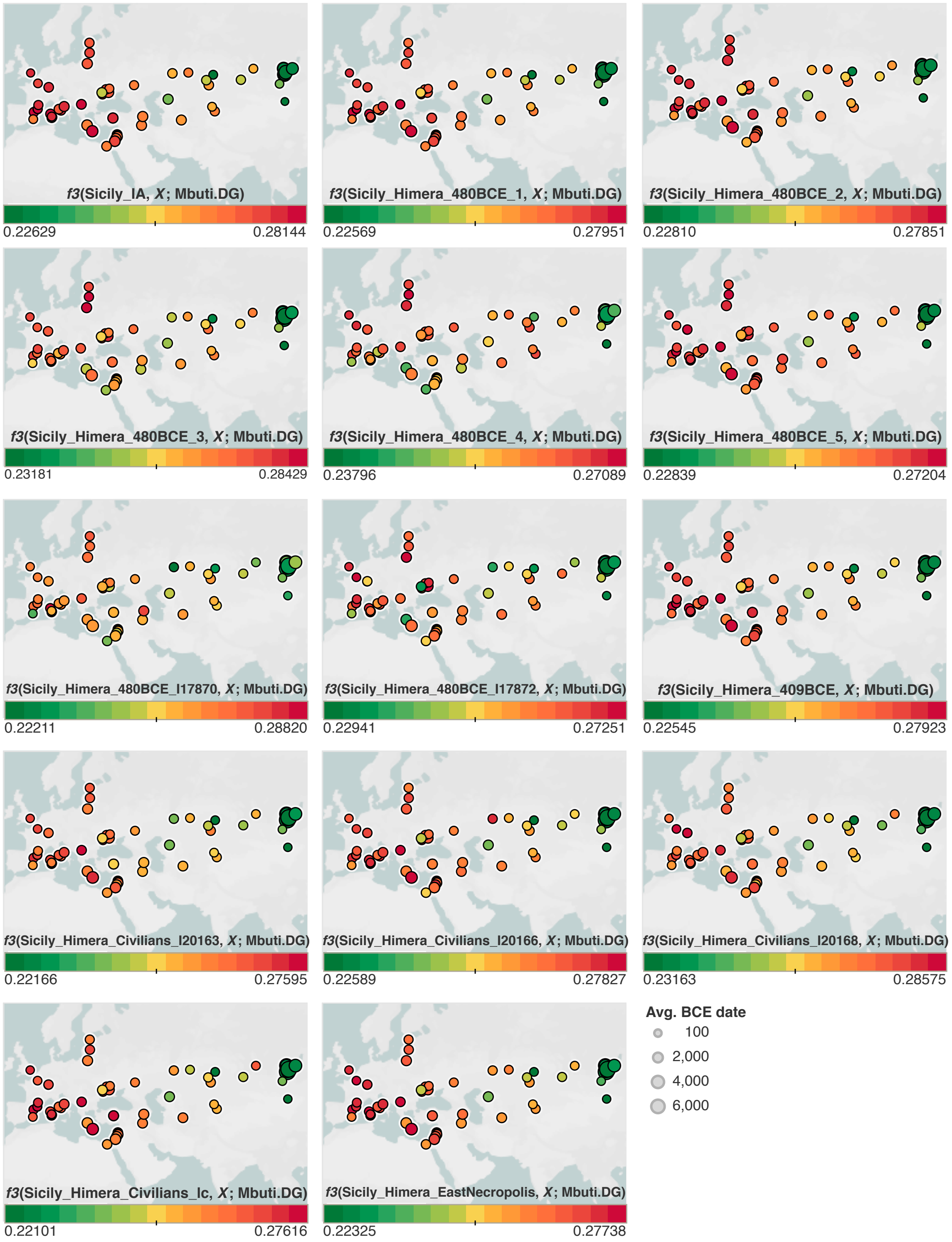

Figure S16. Shared genetic drift of Sicilian groups with Eurasian Eneolithic to IA populations measured by outgroup f_3-statistics. Warmer colors indicate more shared genetic drift.

Dataset S1 (separate file). Overview of sample processing and library statistics

Dataset S2 (separate file). Overview of samples used in analyses.

Dataset S3 (separate file). Overview of newly reported data genotyped on the HO array.

Dataset S4 (separate file). Overview of published samples used as reference in analyses.

Dataset S5 (separate file). Working qpAdm models per individuals with p≥0.01 using P11 as outgroups and as proxies for the distal ancestry sources (1) Turkey_N_Barcin, (2) WHG, (3) Iran_GanjDareh_N/CHG (marked with †), (4) Russia_Samara_EBA_Yamnaya and (5) Russia_Shamanka_Eneolithic.SG.

Dataset S6 (separate file). Y-chromosomal haplogroup assignments.

Dataset S7 (separate file). Phenotype probabilities estimated with Hirisplex-S in 10 replicates. runs.

SI References

1. F. de Angelis, G. R. Tsetskhladze, *The Archaeology of Greek colonization* (Oxford: Oxford Committee for Archaeology, Oxford, 1994).
2. J. Serrati, "Sicily from pre-Greek times to the fourth century." in Sicily from Aeneas to Augustus: New Approaches in Archaeology and History, C. J. Smith, J. Serrati, Eds. (Edinburgh University Press, Edinburgh, 2000), pp. 9-14.
3. T. Hodos, *Local responses to colonization in the Iron Age Mediterranean* (Routledge, New York, 2006).
4. G. R. Tsetskhladze, J. Hargrave, Colonisation from Antiquity to modern times: comparisons and contrasts. *AWE* **10**, 161-182 (2011).
5. T. J. Dunbabin, *The Western Greeks: The History of Sicily and South Italy from the Foundation of the Greek Colonies to 480 B.C* (Clarendon Press, 1948).
6. S. Vassallo, "Indigeni ad Himera? Il ruolo dei Sicani nelle vicende della colonia" in Segni di appartenenza e identità di comunità nel mondo indigeno, G. Greco, B. Ferrara, Eds. (Naus Editoria, 2014), pp. 355-368
7. S. Vassallo, *Himera: Citta Greca; Guida Alla Atoria e Ai Monumenti* (Soprintendenza ai Beni Culturali E Ambientali Servizio Beni Archeologici Palermo, 2005).
8. A. J. Dominguez, "Greeks in Sicily" in Greek Colonisation: An Account of Greek Colonies and Other Settlements Overseas, G. R. Tsetskhladze, Ed. (Koninklijke Brill NV, Leiden, 2006), vol. 1, pp. 253–357.
9. A. J. Dominguez, "Greek Colonisation" in Greeks in Sicily. (Brill, 2006), https://doi.org/10.1163/9789047404101_009, pp. 253-358.
10. R. E. Ray, *Land Battles in 5th Century B.C. Greece: A History and Analysis of 173 Engagements* (McFarland & Company, Inc., Jefferson, 2009).
11. N. Allegro, "Imera" in La Città Greca Antica: Instituzioni, Società e Forme Urbane, E. Greco, Ed. (Donzelli Editore, Rome, 1999), pp. 269-301.
12. N. Allegro, "I blocchi 1-4. Considerazioni generali" in Himera V. (2008), pp. 211-220.
13. B. Bechtold, S. Vassallo, *Le Anfore Puniche dalle Necropoli di Himera (Seconda Metà del VII – Fine del V Sec. a.C.)*, Babesch Annual Papers on Mediterranean Archaeology, Supplement 34 (Peeters, Leuven, 2017).
14. M. Valentino, "Appendice. Anforoni corinzi di età arcaica rinvenuti nelle necropoli di Himera" in Sicilia occidentale: Studi, rassegne, ricerche, C. Ampolo, Ed. (Scuola Normale Superiore Pisa, Pisa, 2012), pp. 59-72.
15. S. Vassallo, Le battaglie di Himera alla luce degli scavi nella necropoli occidentale e alle fortificazioni : i luoghi, i protagonisti. *Sicilia Antiqua* **VII**, 17-38 (2010).

16. F. De Angelis, *Archaic and Classical Greek Sicily: A Social and Economic History.* (Oxford University Press, Oxford, 2016).
17. M. J. Griffin (2005) The tyrannies in the Greek cities of Sicily: 505–466 BC. Ph.D. Dissertation. (University of Leeds).
18. K. Lomas, "Tyrants and the polis: Migration, identity and urban development in Sicily" in Ancient tyranny, S. Lewis, Ed. (Edinburgh University Press, Edinburgh, 2006), pp. 95–117.
19. J. W. Lee, "Warfare in the Classical Age" in A Companion to the Classical Greek World, K. H. Kinzel, Ed. (Oxford: Blackwell, Malden, Mass, 2006), pp. 480-508.
20. F. Bertolino, F. Alaimo, S. Vassallo, Battles of Himera (480 and 409 b.c.): Analysis of Biological Finds and Historical Interpretation. Experiences of Restoration in the Ruins of Himera 2008-2010. *Conservation Science in Cultural Heritage* **15**, 27-40 (2017).
21. B. Kyle, L. J. Reitsema, J. Tyler, P. F. Fabbri, S. Vassallo, Examining the osteological paradox: Skeletal stress in mass graves versus civilians at the Greek colony of Himera (Sicily). *American Journal of Physical Anthropology* **167**, 161-172 (2018).
22. S. Viva *et al.*, The mass burials from the western necropolis of the Greek colony of Himera (Sicily) related to the battles of 480 and 409 BCE. *International Journal of Osteoarchaeology* **30**, 307-317 (2020).
23. P. Fabbri, R. Schettino, S. Vassallo, "Lo scavo delle sepolture della necropoli di Himera Pestavecchia (Palermo)" in Guerra e pace in Sicilia e nel Mediterraneo antico (VIII-III sec. aC): Arte, prassi e teoria della pace e della guerra. Scuola Normale Superiore, Pisa, C. Michelini, Ed. (Edizioni della Normale, Pisa, 2006).
24. L. J. Reitsema, B. Kyle, S. Vassallo, Food traditions and colonial interactions in the ancient Mediterranean: Stable isotope evidence from the Greek Sicilian colony Himera. *Journal of Anthropological Archaeology* **57**, 101144 (2020).
25. S. Vaughn, "The identification and retrieval of the hoplite battle-dead" in Hoplites: the Classical Greek Battle Experience, V. D. Hanson, Ed. (Routledge, London, 1993), pp. 38-63.
26. N. Dimakis, Ancient Greek deathscapes. *Journal of Eastern Mediterranean Archaeology & Heritage Studies* **3**, 27-41 (2015).
27. K. L. Reinberger *et al.*, Isotopic evidence for geographic heterogeneity in Ancient Greek military forces. *PloS One* **16**, e0248803 (2021).
28. G. Shepherd, Dead men tell no tales: Ethnic diversity in Sicilian colonies and the evidence of the cemeteries. *Oxford J. Archaeology* **24**, 115–136 (2005).
29. E. De Miro, *Polizzello centro della Sikania*, Quaderni dell'Istituto di Archeologia dell'Università di Messina (1988), vol. 3.
30. S. Tusa, Ethnic dynamics during pre- and proto-history of Sicily. *J. Cult. Heritage* **1**, 17-28 (2000).
31. R. Di Salvo, A. Messina, V. Schimmenti, L. Sineo, M. A. Mannino (2012) Studio antropologico sul gruppo umano di Polizzello (Mussomeli – CL). in *Atti della XLI Riunione Scientifica dell'Istituto Italiano di Preistoria e Protostoria* (San Cipirello (PA)).
32. O. Belvedere, A. Burgio, G. Bordonaro, V. Forgia, Baucina (Pa) – Monte Falcone 2014 Indagini nella necropoli. *FOLD&R FastiOnLine Documents & Research* **380**, 1-7 (2017).
33. G. Bordonaro, *Carta Archeologica e Sistema Informativo Territoriale del Comune di Baucina, Palermo* (Palermo, 2011).
34. S. Mallick *et al.*, The Simons Genome Diversity Project: 300 genomes from 142 diverse populations. *Nature* **538**, 201-206 (2016).
35. M. Meyer *et al.*, A high-coverage genome sequence from an archaic Denisovan individual. *Science* **338**, 222-226 (2012).
36. K. Prüfer *et al.*, The Complete Genome Sequence of a Neanderthal from the Altai Mountains. *Nature* **505**, 43–49 (2014).
37. P. Skoglund *et al.*, Genetic evidence for two founding populations of the Americas. *Nature* **525**, 104-108 (2015).
38. J. M. Monroy Kuhn, M. Jakobsson, T. Günther, Estimating Genetic Kin Relationships in Prehistoric Populations. *PloS One* **13**, e0195491 (2018).
39. K. J. Galinsky *et al.*, Fast Principal-Component Analysis Reveals Convergent Evolution of ADH1B in Europe and East Asia. *Am J Hum Genet* **98**, 456-472 (2016).

40. I. Lazaridis *et al.*, Ancient human genomes suggest three ancestral populations for present-day Europeans. *Nature* **513**, 409-413 (2014).
41. D. H. Alexander, J. Novembre, K. Lange, Fast model-based estimation of ancestry in unrelated individuals. *Genome Res* **19**, 1655-1664 (2009).
42. W. Haak *et al.*, Massive migration from the steppe was a source for Indo-European languages in Europe. *Nature* **522**, 207-211 (2015).
43. D. M. Fernandes *et al.*, The spread of steppe and Iranian-related ancestry in the islands of the western Mediterranean. *Nat Ecol Evol* **4**, 334-345 (2020).
44. A. Mittnik *et al.*, The genetic prehistory of the Baltic Sea region. *Nat Commun* **9**, 442 (2018).
45. L. Saag *et al.*, The Arrival of Siberian Ancestry Connecting the Eastern Baltic to Uralic Speakers further East. *Curr Biol* **29**, 1701-1711 e1716 (2019).
46. P. de Barros Damgaard *et al.*, 137 Ancient Human Genomes from across the Eurasian Steppes. *Nature* **557** (2018).
47. M. Krzewinska *et al.*, Ancient genomes suggest the eastern Pontic-Caspian steppe as the source of western Iron Age nomads. *Sci Adv* **4**, eaat4457 (2018).
48. M. Jarve *et al.*, Shifts in the Genetic Landscape of the Western Eurasian Steppe Associated with the Beginning and End of the Scythian Dominance. *Curr Biol* **29**, 2430-2441 e2410 (2019).
49. Ø. Hammer, Harper, D.A.T., Ryan, P.D., PAST: Paleontological statistics software package for education and data analysis. *Palaeontologia Electronica* **4**, 9 (2001).
50. M. J. Anderson, A new method for non-parametric multivariate analysis of variance. *Austral. Ecology* **26**, 32–46 (2001).
51. H. Ringbauer, J. Novembre, M. Steinrücken, Human Parental Relatedness through Time - Detecting Runs of Homozygosity in Ancient DNA. *bioRxiv* 10.1101/2020.05.31.126912, 2020.2005.2031.126912 (2020).
52. L. Chaitanya *et al.*, The HIrisPlex-S system for eye, hair and skin colour prediction from DNA: Introduction and forensic developmental validation. *Forensic Science International: Genetics* **35**, 123-135 (2018).
53. S. Walsh *et al.*, Global skin colour prediction from DNA. *Human genetics* **136**, 847-863 (2017).
54. S. Walsh *et al.*, Developmental validation of the HIrisPlex system: DNA-based eye and hair colour prediction for forensic and anthropological usage. *Forensic Sci Int Genet* **9**, 150-161 (2014).
55. I. Lazaridis *et al.*, Genetic origins of the Minoans and Mycenaeans. *Nature* **548**, 214-218 (2017).
56. N. S. Enattah *et al.*, Identification of a variant associated with adult-type hypolactasia. *Nat Genet* **30**, 233-237 (2002).
57. T. Bersaglieri *et al.*, Genetic signatures of strong recent positive selection at the lactase gene. *Am J Hum Genet* **74**, 1111-1120 (2004).
58. M. E. Allentoft *et al.*, Population genomics of Bronze Age Eurasia. *Nature* **522**, 167-172 (2015).
59. I. Mathieson *et al.*, Genome-wide patterns of selection in 230 ancient Eurasians. *Nature* **528**, 499-503 (2015).
60. J. Burger *et al.*, Low Prevalence of Lactase Persistence in Bronze Age Europe Indicates Ongoing Strong Selection over the Last 3,000 Years. *Curr Biol* **30**, 4307-4315 e4313 (2020).
61. A. Fujimoto *et al.*, A scan for genetic determinants of human hair morphology: EDAR is associated with Asian hair thickness. *Hum Mol Genet* **17**, 835-843 (2008).
62. Y. G. Kamberov *et al.*, Modeling recent human evolution in mice by expression of a selected EDAR variant. *Cell* **152**, 691-702 (2013).
63. R. Kimura *et al.*, A common variation in EDAR is a genetic determinant of shovel-shaped incisors. *Am J Hum Genet* **85**, 528-535 (2009).
